# Circadian gene CLOCK-mediated LLPS of NBR1 and p62/SQSTM1 inhibits proliferation and migration of nasopharyngeal carcinoma

**DOI:** 10.1038/s41419-026-08957-x

**Published:** 2026-06-10

**Authors:** Nasot Rashed, Ya Zhu, Jing Quan, Zhengjiang Li, Pai Zeng, Hongde Li, Wenbin Liu, Xi Zeng, Xiangjian Luo

**Affiliations:** 1 Hunan Key Laboratory of Oncotarget Gene, Hunan Cancer Hospital and the Affiliated Cancer Hospital of Xiangya School of Medicine, Central South University, Changsha, Hunan 410013 PR China; 2 Key Laboratory of Carcinogenesis and Invasion, Chinese Ministry of Education, Cancer Research Institute, Xiangya School of Basic Medical Science, Central South University, Changsha, Hunan 410078 PR China; 3 Department of Infectious Diseases, Nanfang Hospital, Southern Medical University, Guangzhou, PR China; 4 The Hunan Provincial College of Clinical Laboratory, Changsha Medical University, Changsha, Hunan 410219 China; 5 Hunan Provincial Key Laboratory of the Traditional Chinese Medicine Agricultural Biogenomics, Changsha Medical University, Changsha, Hunan 410219 China; 6 Department of Pathology, Hunan Cancer Hospital and The Affiliated Cancer Hospital of Xiangya School of Medicine, Central South University, Changsha, Hunan 410013 PR China; 7 Hunan Province Key Laboratory of Tumor Cellular & Molecular Pathology, Cancer Research Institute, Hengyang Medical School, University of South China, Hengyang, Hunan 421001 China; 8 Key Laboratory of Biological Nanotechnology of National Health Commission, Central South University, Changsha, Hunan 410078 China

**Keywords:** Cancer, Cell biology, Molecular biology

## Abstract

Circadian Locomotor Output Cycles Kaput (CLOCK) is a core circadian gene encoding a transcription factor essential for maintaining physiological rhythms, and has more recently been implicated in cancer biology. In this study, we uncover an unrecognized tumor-suppressive role of CLOCK in nasopharyngeal carcinoma (NPC). Mechanistically, CLOCK directly activates NBR1 transcription, leading to the stabilization of p62/SQSTM1 and the co-assembly of NBR1-p62 condensates via liquid-liquid phase separation (LLPS). These condensates serve as scaffolds for the recruitment of the E3 ubiquitin ligase TRIM38, which mediates the ubiquitination and proteasomal degradation of TAK1-binding protein 2 (TAB2), thereby attenuating NF-κB signaling. Functionally, this axis suppresses NPC cell proliferation and migration. Our findings reveal a novel CLOCK–NBR1/p62–TRIM38–TAB2 pathway that links circadian gene to LLPS-driven tumor suppression in NPC, providing new insights into the role of circadian genes in cancer biology and pointing to a potential therapeutic target for this malignancy.

**Figure:**
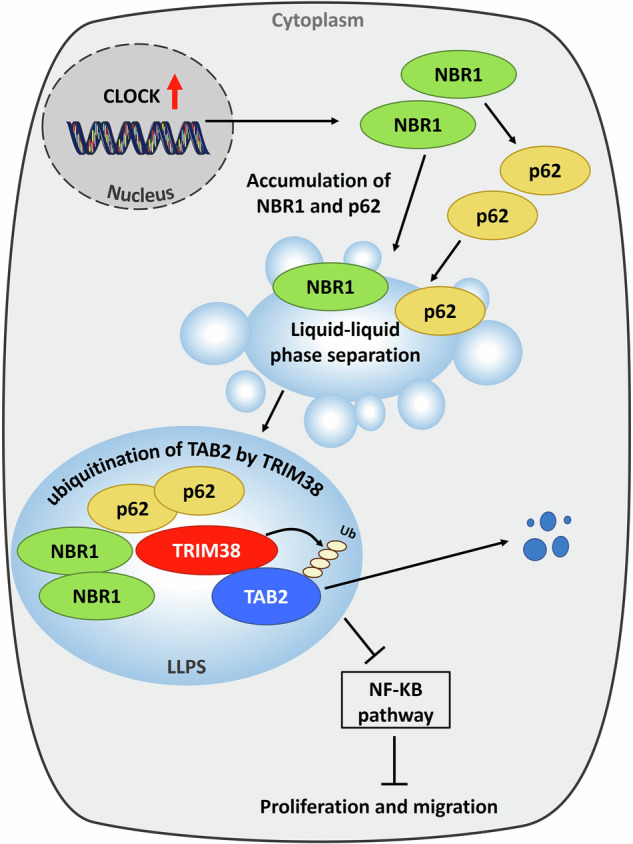

## Introduction

NPC is an epithelial malignancy with a strikingly uneven geographic distribution, exhibiting high incidence across Southeast Asia and southern China [1, 2]. Despite advances in radiotherapy and combined-modality treatments, a substantial subset of patients experiences disease progression driven by pro-survival inflammatory signaling, metastatic dissemination, and therapy resistance [1, 3]. These features underscore the need to delineate tumor-intrinsic pathways that can be therapeutically leveraged to restrain NPC growth and dissemination.

Circadian clock genes orchestrate daily physiological rhythms and cellular homeostasis, and their dysregulation has been implicated in tumorigenesis across diverse malignancies. Among these, CLOCK represents a core transcription factor, canonically partnering with BMAL1 to regulate clock-controlled genes [4, 5]. While initially characterized as the first circadian gene in 1994, CLOCK has since been shown to extend its influence beyond circadian regulation to broader roles in cellular physiology and disease [6, 7]. In cancer, CLOCK overexpression has been reported in several malignancies and is frequently associated with tumor-promoting features, including enhanced proliferation, migration, and therapy resistance [8–15]. However, its role in NPC has not yet been investigated, representing an important gap in current knowledge.

Recent studies have highlighted the biological significance of biomolecular condensates formed through LLPS in regulating oncogenic signaling [16, 17]. These membraneless compartments assemble stress-response and signaling molecules into dynamic hubs, thereby controlling proteostasis and cellular adaptation. Among the best-characterized scaffolds are the selective autophagy receptors NBR1 and p62/SQSTM1, which interact through their Phox and Bem1p (PB1) domains to form LLPS condensates [18–20]. These structures mediate clustering of ubiquitinated proteins, facilitate autophagic degradation, and regulate key stress and signaling pathways. Notably, LLPS mediated by NBR1 and p62 intersects with critical signaling pathways in cancer, yet the upstream transcriptional mechanisms governing condensate formation remain poorly defined.

TAB2 is a crucial adaptor protein that mediates NF-κB activation by linking upstream kinases, such as TAK1, to the IKK complex [21, 22]. Through this scaffolding role, TAB2 facilitates IκB phosphorylation followed by its degradation, thereby allowing NF-κB subunits to translocate into the nucleus and initiate transcriptional programs that support cell survival, proliferation, inflammation, and tumor progression [23, 24]. Compared to other regulatory components of the NF-κB pathway, TAB2 functions as a central signaling hub, such that disturbances in its stability or activity can profoundly disrupt NF-κB signaling, underscoring its importance as a key regulatory node. Notably, TAB2 has also been reported to exert tumor-suppressive functions in specific contexts by negatively regulating NF-κB activity, highlighting its context-dependent role in cancer biology. Collectively, these mechanisms converge on the NF-κB pathway, a master regulator of cellular adaptation and malignant progression [24–26].

In this study, we demonstrate that CLOCK functions as a tumor suppressor in NPC, in contrast to its reported oncogenic roles in other cancers types. Mechanistically, we show that CLOCK transcriptionally activates NBR1, which cooperates with p62/SQSTM1 to form LLPS condensates. These condensates serve as a platform for the recruitment of TRIM38, which ubiquitinates TAB2 and attenuates NF-κB signaling. Through this pathway, CLOCK suppresses NPC cell proliferation and migration. Our findings uncover a previously unrecognized CLOCK–NBR1/p62–TRIM38–TAB2 axis that mechanistically links circadian transcriptional regulation to LLPS-mediated tumor suppression in NPC.

## Materials and methods

### Cell culture

NPC cell lines (HONE1, C666-1, CNE1, and HNE2) were cultured in RPMI-1640 medium (Hyclone, Logan, UT, USA) supplemented with 10% fetal bovine serum and 1% penicillin-streptomycin; HEK293T cells were cultured in DMEM (Hyclone, Logan, UT, USA) with the same supplements; and NP69 nasopharyngeal epithelial cells were cultured in keratinocyte serum-free medium (Invitrogen, Carlsbad, CA, USA). All cells were maintained at 37 °C in a humidified atmosphere containing 5% CO₂. Cells were routinely screened for mycoplasma contamination using the MycoBlue Mycoplasma Detection Kit (Vazyme). Authentication of all cell lines was verified by short tandem repeat (STR) profiling, which was performed by Shanghai Biowing Applied Biotechnology (Shanghai, China).

### Quantitative RT-PCR

Total RNA was isolated using TRIzol® reagent (Ambion®, Life Technologies, USA). Reverse transcription was performed using the RevertAid First Strand cDNA Synthesis Kit (catalog number K1622, Invitrogen, Carlsbad, CA, USA). Real-time PCR analysis was carried out in triplicate using ChamQ Blue Universal SYBR qPCR Master Mix (catalog number Q312-02, Vazyme, Nanjing, China) on an ABI 7500 Real-Time System (Applied Biosystems). All primers used in this study are listed in Supplementary Table S1.

### Plasmids and lentiviral transduction

Lentiviral constructs for CLOCK overexpression (subcloned into pLVX-EF1α-IRES-Puro) and shRNA-mediated silencing of CLOCK, NBR1, SQSTM1/p62, and TRIM38 were generated and packaged in HEK293T cells according to standard protocols. NPC cells were transduced with viral supernatants in the presence of 8 μg/mL polybrene and selected with puromycin to establish stable cell lines. For plasmid-based assays, coding sequences of NBR1, p62, CLOCK, and TRIM38 were amplified by PCR from cellular cDNA and cloned into the pCMV-Flag-N, pEGFP-C3, and pCMV-Myc-N vectors. The NBR1 promoter was amplified from genomic DNA and inserted into the pGL6-TA reporter vector. Mutant promoter constructs were generated by site-directed mutagenesis of the predicted CLOCK-binding sites (JASPAR database), and sequence-verified clones were used in subsequent functional assays. All transfections were performed using Lipomax transfection reagent according to the instructions provided by the manufacturer. Primer sequences used for cloning and mutagenesis are listed in Supplementary Table S2.

### CCK-8 cell viability assay

NPC cells were seeded into 96-well plates and cultured under standard conditions. Cell viability was assessed using the Cell Counting Kit-8 (CCK-8, Dojindo, Japan) according to the manufacturer’s instructions. A mixture of culture medium and CCK-8 reagent (9:1) was prepared, and 100 μL was added to each well. Following incubation at 37 °C with 5% CO₂ for 30–60 min, absorbance at 450 nm was measured using a microplate reader (BioTek EL×800, USA).

### Colony formation assay

NPC cells were seeded into 6-well plates at a density of 1000 cells per well and maintained at 37 °C in a humidified incubator with 5% CO₂. After 15–20 days of culture, colonies were gently washed with PBS, fixed in methanol for 10 min, and stained with crystal violet for 15 min at room temperature. Plates were then rinsed, air-dried, and imaged using a digital camera. Colonies containing more than 50 cells were quantified using ImageJ software, and survival fractions were calculated. Survival curves were generated with GraphPad Prism version 5 (GraphPad Software, La Jolla, CA, USA).

### Wound healing assay

NPC cells in the logarithmic growth phase were digested with 0.25% trypsin and seeded into 6-well plates at a density of 5 × 10⁵ cells per well, then incubated overnight at 37 °C in a humidified atmosphere containing 5% CO₂. A linear scratch was created in the confluent monolayer using a 200 μL pipette tip, and floating cells were removed by washing twice with PBS before adding serum-free medium. Cells were maintained under identical culture conditions for an additional 48 h. Wound closure was then monitored using an inverted microscope, and representative images were captured. The migratory capacity of NPC cells was quantified by calculating the healing rate (HR = [(scratch area at 0 h − scratch area at the indicated time)/scratch area at 0 h] × 100%) using ImageJ software. All experiments were performed in triplicate to ensure reproducibility.

### Transwell migration assay

Cell migration was assessed using Transwell chambers (8 μm pore size, polycarbonate membrane). Briefly, NPC cells were harvested with 0.25% trypsin, resuspended in serum-free RPMI-1640 medium, and seeded into the upper chamber at a density of 1 × 10⁴ cells (1 × 10⁵ cells/mL). The lower chamber was filled with 600 μL RPMI-1640 supplemented with 10% FBS to serve as a chemoattractant. After 24 h of incubation at 37 °C in a humidified atmosphere containing 5% CO₂, non-migrated cells on the upper surface of the membrane were gently removed using a cotton swab. Migrated cells adherent to the lower surface were fixed in methanol for 15 min, stained with 0.1% crystal violet for 15 min at room temperature, and washed thoroughly. Images were captured using an inverted microscope, and migrated cells were quantified by counting five randomly selected fields per membrane. All experiments were independently repeated three times.

### Dual luciferase reporter assay

HONE1, and C666-1 cells were seeded into 60 mm culture dishes and grown to approximately 70–90% confluence. Transfections were performed using Lipomax reagent according to the manufacturer’s instructions. For experimental conditions, 1 μg of the promoter fragment reporter plasmid was co-transfected with 100 ng of the pRL-TK plasmid, which served as an internal control. Control cells were transfected with an equivalent amount of the empty pGL6-TA vector. After 48 h, cells were harvested, and firefly and Renilla luciferase activities were quantified using a dual-luciferase reporter assay system.

### Co-immunoprecipitation (Co-IP)

HEK293T, HONE1, and C666-1 cells were seeded in 10 cm culture dishes and cultured for approximately 12 h until reaching 70–90% confluence. Plasmid transfections were performed using Lipomax transfection reagent according to the manufacturer’s instructions. Six hours post-transfection, the medium was replaced with 3% serum-containing medium, and cells were further incubated for 48 h. Cells were then lysed at 4 °C for 2 h using immunoprecipitation lysis buffer supplemented with protease inhibitor cocktail, and lysates were cleared by centrifugation at 12,000 rpm for 5 min at 4 °C. The supernatants were pre-cleared with protein A/G magnetic beads for 1 h at 4 °C to remove nonspecific binding proteins. Subsequently, 2 μg of the indicated antibody was added to the pre-cleared lysates and incubated overnight (12–15 h) at 4°C to form immune complexes. Thirty microliters of protein A/G beads were then added and incubated for 2–3 h at 4 °C to capture antibody-protein complexes. Beads were washed five times with ice-cold PBS, and bound proteins were eluted by boiling in 5× SDS loading buffer for 10 min. Immunoprecipitated proteins were analyzed by Western blotting using the indicated primary antibodies.

### Indirect immunofluorescence assay (IFA)

HONE1 cells were seeded in 35 mm glass-bottomed dishes and grown for approximately 12 h until reaching 50–60% confluence, and then transfected using Lipomax transfection reagent. Forty-eight hours post-transfection, cells were fixed with 4% paraformaldehyde for 20 min at room temperature. After discarding the fixative, cells were permeabilized with 0.2% Triton X-100 in skimmed milk for 30 min at 37 °C and subsequently washed five times with PBS. Primary antibodies, diluted in 5% skimmed milk, were then added and incubated at 37 °C for 4 h. Following five washes with autoclaved PBS, cells were incubated with the corresponding fluorophore-conjugated secondary antibodies diluted in 5% skimmed milk at 37 °C for 2 h. Cells were washed five times with PBS, counterstained with DAPI for 10 min at room temperature, and fluorescence signals were detected and imaged using a Leica laser scanning confocal microscope.

### Animal studies

Four-week-old BALB/c nude mice (*n* = 7 per group) were subcutaneously injected with either C666-1 control cells (C666-1-NC) or CLOCK-overexpressing C666-1 cells (C666-1-CLOCK). The sample size for animal experiments was determined based on the study design and experimental objectives. Animals were randomly allocated to the indicated groups, and investigators were blinded to group assignments throughout the experimental procedures. Tumor growth was monitored daily, and tumor volume was calculated using the formula $V=\mathrm{length}\times {\mathrm{width}}^{2}/2$. After two weeks, mice were sacrificed, and tumors were excised, weighed, and subjected to Western blot and immunohistochemical analyses. All animal experiments were performed in accordance with institutional guidelines for the care and use of laboratory animals and were approved by the Animal Care and Use Committee of the Cancer Research Institute, Central South University, Changsha, PR China.

### Statistical analysis

Data are presented as mean ± standard deviation (SD). Statistical significance was determined using Student’s *t* test or ANOVA, as appropriate, with GraphPad Prism 10. A *p* value < 0.05 was considered statistically significant. Statistical significance is indicated in the figures as follows: *p < 0.05, * *p < 0.01, * * *p < 0.001, and ns, not significant.

## Results

### Bioinformatic identification of CLOCK as a prognostic protective gene in head and neck squamous cell carcinoma (HNSCC)

**Fig. 1:**
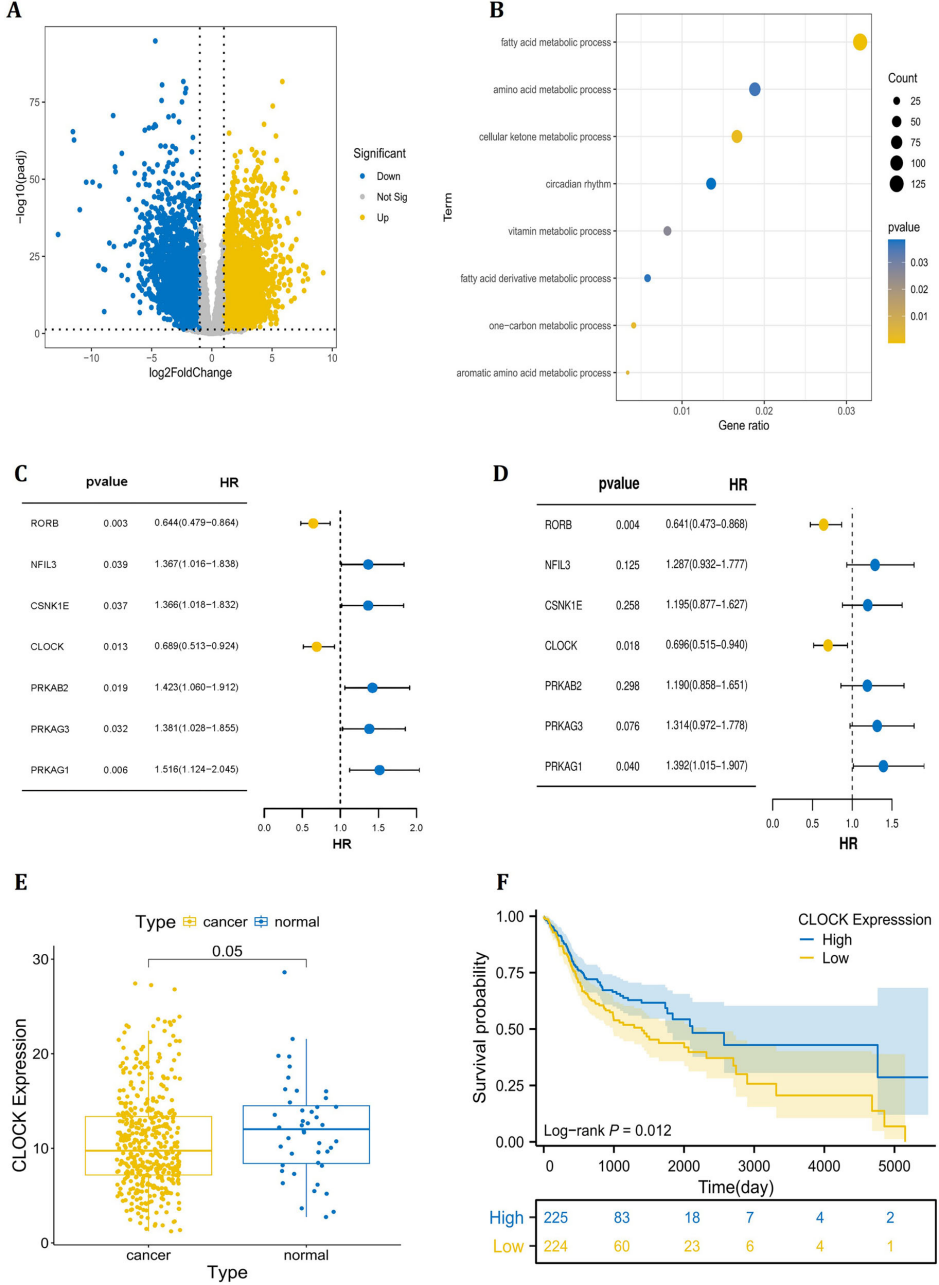
Differential expression and prognostic analysis of CLOCK in HNSCC. **A** Volcano plot of DEGs between tumor and normal samples in the TCGA-HNSC cohort. **B** GO enrichment analysis showing significant enrichment of DEGs in the circadian rhythm pathway. **C** Univariate and **D** multivariate Cox regression analyses of circadian rhythm-related genes, identifying CLOCK as a protective factor in HNSCC. **E** Box plot showing significantly lower CLOCK expression in tumor tissues compared with normal tissues. **F** Kaplan-Meier survival curves indicating that high CLOCK expression is associated with better overall survival. DEGs differentially expressed genes.

To comprehensively characterize the transcriptional alterations in HNSCC, we conducted bioinformatic analysis of the TCGA-HNSC transcriptomic dataset. Differential expression profiling between primary tumor tissues and matched adjacent normal counterparts revealed a distinct set of dysregulated genes (DEGs) (Fig. 1A). Gene Ontology (GO) enrichment analysis indicated that these DEGs were markedly enriched in circadian rhythm associated pathways (Fig. 1B), highlighting the potential involvement of circadian regulators in tumorigenesis. To explore the prognostic relevance of circadian rhythm-related genes, we retrieved candidate genes from the KEGG pathway database and subjected them to univariate Cox proportional hazards regression analysis. Among the identified genes, seven, including RORB and CLOCK, showed significant associations with overall survival, with CLOCK emerging as a statistically significant protective factor (Fig. 1C). Multivariate Cox regression analysis further substantiated this association, confirming CLOCK as an independent prognostic indicator (Fig. 1D). Expression profiling revealed a pronounced downregulation of CLOCK in tumor tissues compared with adjacent normal tissues (Fig. 1E). Kaplan-Meier survival analysis demonstrated that patients with high CLOCK expression exhibited significantly better overall survival than those with low expression (Fig. 1F).

NPC is a distinct subtype of HNSCC, providing the rationale to investigate whether these circadian alterations extend to NPC. Given the observed downregulation and prognostic value of CLOCK in HNSCC, it is reasonable to hypothesize that CLOCK functions as a tumor suppressor in NPC. Collectively, these bioinformatic results nominate CLOCK as a putative tumor suppressor, where its reduced expression in tumors and association with improved clinical outcomes highlight its potential protective role. This evidence establishes a strong foundation for subsequent functional validation of CLOCK’s anti-tumorigenic role in NPC.

### CLOCK inhibits the proliferation and migration of NPC cells

**Fig. 2:**
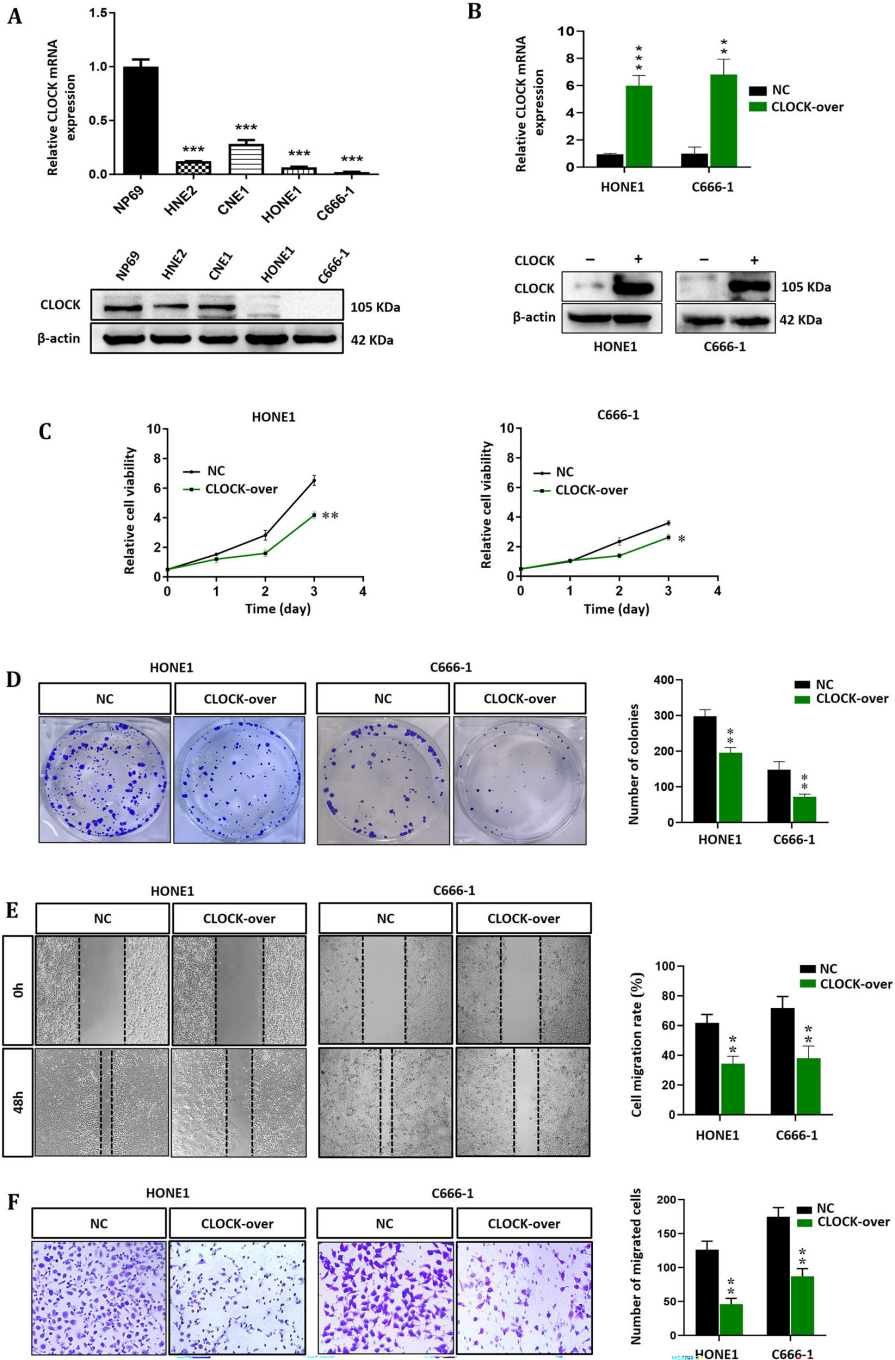
CLOCK inhibits NPC Cell proliferation and migration. qRT-PCR and Western blot analyses were performed to determine CLOCK mRNA and protein expression levels in NPC cell lines (HNE2, CNE1, HONE1, and C666-1) and the normal nasopharyngeal epithelial cell line NP69 (**A**), and to confirm CLOCK overexpression efficiency in stably transfected HONE1 and C666-1 cells (**B**). **C** The CCK-8 assay was performed to assess cell viability in HONE1-NC, HONE1-CLOCK, C666-1-NC, and C666-1-CLOCK cells at days 0, 1, 2, and 3. **D** Colony formation assays were conducted to evaluate clonogenic capacity, with colonies fixed, stained, and counted after 15–20 days of culture. **E**, **F** Wound healing and transwell migration assays were performed to assess cell migration following CLOCK overexpression. Wound closure was evaluated at 0 and 48 h (**E**). For the transwell assay, cells were fixed and stained with crystal violet at 24 h (**F**). Data are presented as mean ± SD from three independent experiments. *, **, and *** indicate *p* < 0.05, *p* < 0.01, and *p* < 0.001, respectively.

To investigate the functional role of CLOCK in NPC, we first quantified its mRNA and protein expression levels in four NPC cell lines (HNE2, CNE1, HONE1, and C666-1) and compared them with those in the normal nasopharyngeal epithelial cell line NP69. Both qRT-PCR and Western blot analyses revealed a marked reduction in CLOCK expression across all NPC cell lines. Notably, CLOCK expression was almost undetectable in HONE1 and C666-1 cells, whereas NP69 cells exhibited relatively high expression levels (Fig. 2A). To functionally assess the impact of CLOCK loss, we generated HONE1 and C666-1 cell lines stably overexpressing CLOCK via lentiviral transduction. Successful transgene integration and robust CLOCK expression were confirmed by qRT-PCR and Western blot analyses (Fig. 2B). Functional assays demonstrated that CLOCK overexpression significantly suppressed NPC cell proliferation, as assessed by CCK-8 assays (Fig. 2C), and markedly impaired clonogenic capacity, as evidenced by a pronounced reduction in colony formation (Fig. 2D). Furthermore, wound healing and Transwell migration assays revealed that CLOCK-overexpressing cells displayed delayed wound closure at 48 h and a substantial decrease in the number of migrated cells compared with negative control cells (Fig. 2E, F).

Our data reveal that CLOCK expression is markedly reduced in NPC cells, with the most pronounced downregulation observed in the HONE1 and C666-1 cell lines. Reconstitution of CLOCK expression potently suppressed cell proliferation and migration, indicating that CLOCK loss contributes to NPC aggressiveness and supporting its function as a tumor suppressor.

### CLOCK knockdown enhances proliferation and migration in NPC cells

**Fig. 3:**
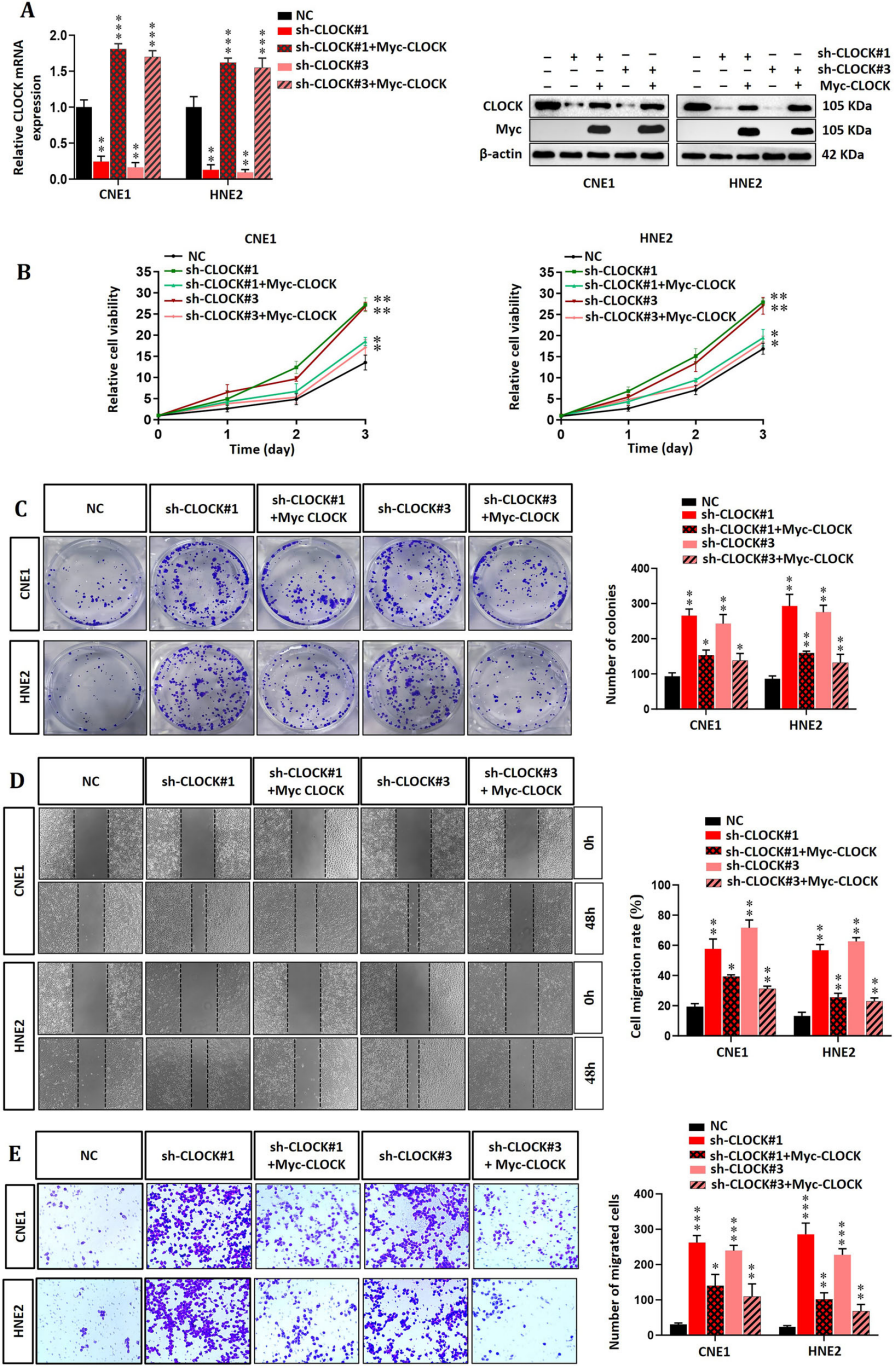
CLOCK regulates NPC cell proliferation and migration. **A** qRT-PCR and Western blot analyses were performed to confirm efficient CLOCK knockdown and subsequent rescue in CNE1 and HNE2 cells. Cells were transduced with negative control shRNA (NC), sh-CLOCK#1, or sh-CLOCK#3, with rescue achieved by ectopic expression of Myc-CLOCK where indicated. **B** Cell viability was assessed using the CCK-8 assay at days 0, 1, 2, and 3. **C** Colony formation assays were conducted to evaluate clonogenic capacity, with colonies fixed, stained, and counted after 15–20 days of culture. **D**, **E** Wound healing and transwell migration assays were performed to assess cell migration. Data are presented as mean ± SD from three independent experiments. *, **, and *** indicate *p* < 0.05, *p* < 0.01, and *p* < 0.001, respectively.

Given that CLOCK overexpression suppresses proliferation and migration in NPC cells lacking endogenous expression, we next investigated whether depletion of CLOCK would conversely enhance oncogenic phenotypes in NPC cells retaining moderate endogenous CLOCK levels. CNE1 and HNE2 cells were therefore selected for loss-of-function analyses. CLOCK expression was stably silenced using two independent shRNAs (sh-CLOCK#1 and sh-CLOCK#3), and rescue experiments were subsequently performed by ectopic expression of Myc-tagged CLOCK in the corresponding knockdown backgrounds. Accordingly, cells were divided into five experimental groups: negative control, sh-CLOCK#1, sh-CLOCK#1 + Myc-CLOCK, sh-CLOCK#3, and sh-CLOCK#3 + Myc-CLOCK. Efficient knockdown and rescue of CLOCK expression were confirmed by qRT-PCR and Western blot analyses (Fig. 3A). Functional assays revealed that CLOCK silencing significantly enhanced NPC cell proliferation, as determined by CCK-8 assays, whereas reintroduction of Myc-CLOCK markedly attenuated this proliferative advantage (Fig. 3B). Consistently, colony formation assays demonstrated a pronounced increase in clonogenic capacity upon CLOCK knockdown, which was largely reversed following CLOCK restoration in both shRNA backgrounds (Fig. 3C). Moreover, wound healing assays showed accelerated wound closure in CLOCK-depleted cells, indicative of enhanced migratory capacity, while Myc-CLOCK re-expression substantially delayed wound closure, restoring migration rates toward those observed in control cells (Fig. 3D). In line with these findings, Transwell migration assays further confirmed that CLOCK knockdown significantly increased the number of migrated cells in both CNE1 and HNE2 cell lines, an effect that was effectively reversed upon CLOCK rescue (Fig. 3E).

Functional depletion of endogenous CLOCK significantly enhanced NPC cell proliferation and migratory capacity, underscoring its intrinsic inhibitory role in tumor aggressiveness. Importantly, re-expression of CLOCK fully reversed these phenotypes, confirming the specificity of CLOCK-dependent regulation and further supporting its tumor-suppressive function in NPC.

### Identification of NBR1 as a direct transcriptional target of CLOCK in NPC

**Fig. 4:**
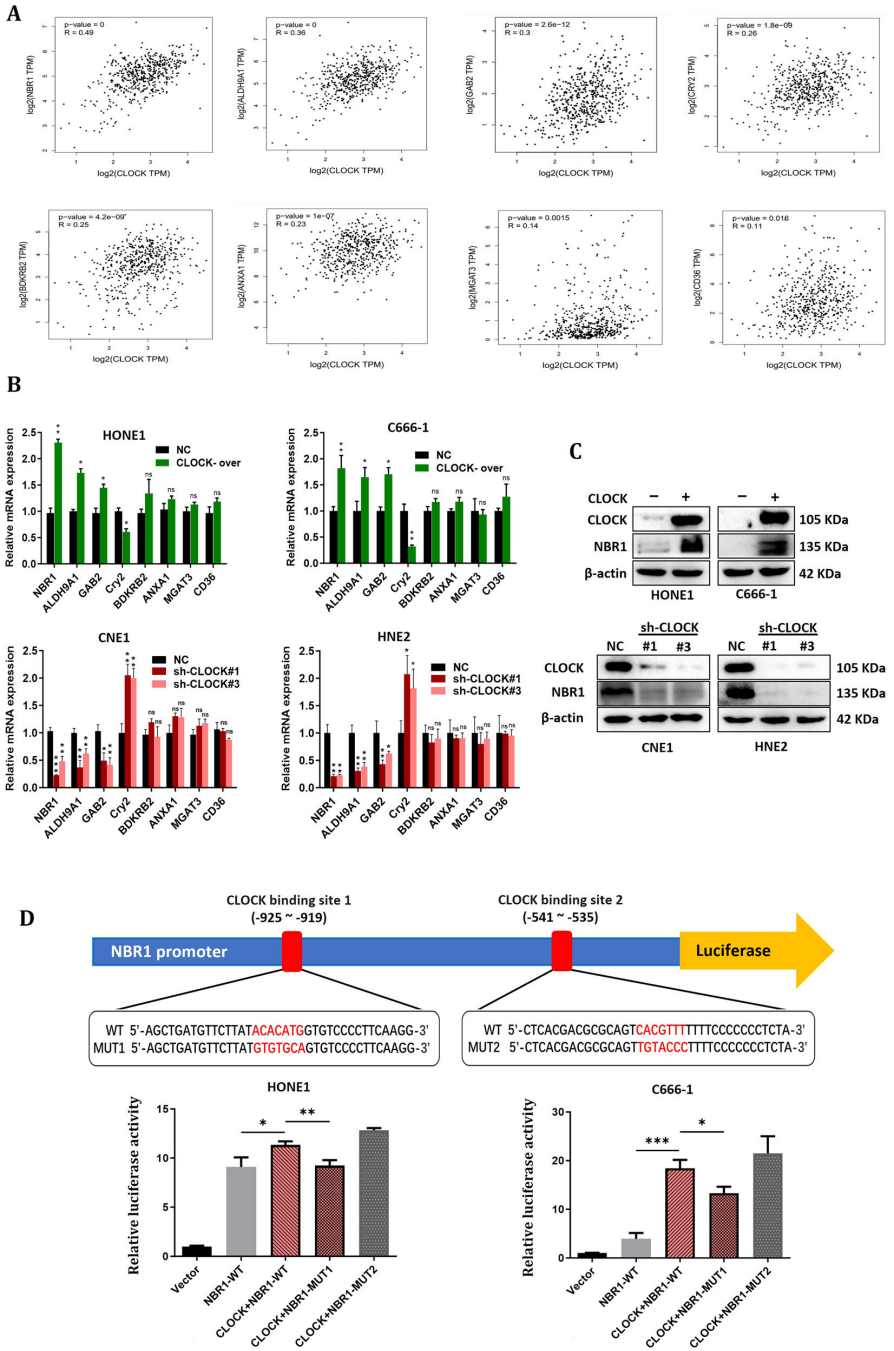
CLOCK transcriptionally upregulates NBR1 expression in NPC. **A** Correlation analysis between CLOCK expression and selected target genes (NBR1, ALDH9A1, GAB2, CRY2, BDKRB2, ANXA1, MGAT3, and CD36) was performed using the GEPIA web tool based on TPM values from the TCGA-HNSC dataset (*n* = 500). **B** RT-qPCR was performed to measure the mRNA expression levels of CLOCK target genes in HONE1 and C666-1 cells following CLOCK overexpression, and in CNE1 and HNE2 cells following CLOCK knockdown. **C** NBR1 protein expression was examined by Western blot in the same cells under CLOCK overexpression or knockdown conditions. **D** Luciferase reporter assays were performed in HONE1 and C666-1 cells to assess the transcriptional regulation of the NBR1 promoter by CLOCK. Data are presented as mean ± SD from three independent experiments. *, **, and *** indicate *p* < 0.05, *p* < 0.01, and *p* < 0.001, respectively; ns indicates no significant difference. TPM transcript per million.

Based on previous studies identifying NBR1, ALDH9A1, GAB2, CRY2, BDKRB2, ANXA1, MGAT3, and CD36 as potential CLOCK-regulated genes [27–30], we sought to determine which of these candidates mediates the tumor-suppressive function of CLOCK in NPC. To this end, we first performed correlation analyses using transcriptomic data from the TCGA-HNSC dataset (*n* = 500), accessed via the GEPIA web server, based on log₂-transformed transcripts per million (TPM) values. Pearson correlation analysis revealed that CLOCK expression was positively correlated with seven of the eight candidate genes (all *p* < 0.001), with NBR1 showing the strongest association (R = 0.49) (Fig. 4A). ALDH9A1 (R = 0.36) and GAB2 (R = 0.30) exhibited moderate correlations, whereas CD36 displayed a weak association (R = 0.11, *p* = 0.016). To determine whether these correlations reflect functional regulation, we next examined the impact of CLOCK modulation on gene expression in NPC cell lines. In HONE1 and C666-1 cells, which exhibit low endogenous CLOCK expression, enforced overexpression of CLOCK significantly upregulated the mRNA levels of NBR1, ALDH9A1, and GAB2. Conversely, in CNE1 and HNE2 cells, CLOCK knockdown resulted in a marked reduction in the expression of these genes (Fig. 4B). Notably, the magnitude of NBR1 regulation were more pronounced compared to the other two candidates. To further validate these observations in clinical samples, we analyzed an independent NPC cohort from the GSE102349 dataset (*n* = 113). Spearman correlation analysis confirmed a statistically significant although weak positive correlation between CLOCK and NBR1 (ρ = 0.186, *P* = 0.0485) (Supplementary Fig. S1A), suggesting that NBR1 may be a potential CLOCK-regulated gene in NPC, although the effect size is modest. In contrast, ALDH9A1 (ρ = 0.16, *P* = 0.079) and GAB2 (ρ = 0.03, *P* = 0.75) showed no statistically significant correlation with CLOCK (Supplementary Fig. S1B, C). This discrepancy further supports the consistency of the CLOCK–NBR1 relationship. Moreover, the transcriptional changes between CLOCK and NBR1 were mirrored at the protein level, as confirmed by Western blot analysis (Fig. 4C), further demonstrating that CLOCK positively regulates NBR1 expression in NPC cells.

To assess the underlying mechanism how CLOCK regulates NBR1 at the transcriptional level, we performed dual-luciferase reporter assays using constructs containing the wild-type or mutated NBR1 promoter. Bioinformatic analysis using the JASPAR database identified two potential CLOCK-binding sites within the NBR1 promoter. Site-directed mutagenesis was used to generate two mutant constructs, NBR1-MUT1 and NBR1-MUT2, in which each predicted binding site was individually disrupted. Co-transfection of CLOCK with the NBR1-WT construct significantly increased luciferase activity in both HONE1 and C666-1 cells. Notably, mutation of site 1 (NBR1-MUT1) markedly attenuated CLOCK-induced transcriptional activation, whereas mutation of site 2 (NBR1-MUT2) had no effect (Fig. 4D). These results indicate that CLOCK-mediated activation of NBR1 transcription primarily depends on a specific functional binding site within the NBR1 promoter.

Mechanistically, NBR1 was identified as a transcriptional target of CLOCK in NPC cells. CLOCK positively regulated NBR1 expression at both mRNA and protein levels via the functional binding sites in the NBR1 promoter, establishing a transcriptional regulatory relationship between CLOCK and NBR1.

### NBR1 mediates the tumor-suppressive effects of CLOCK on NPC cell proliferation and migration

**Fig. 5:**
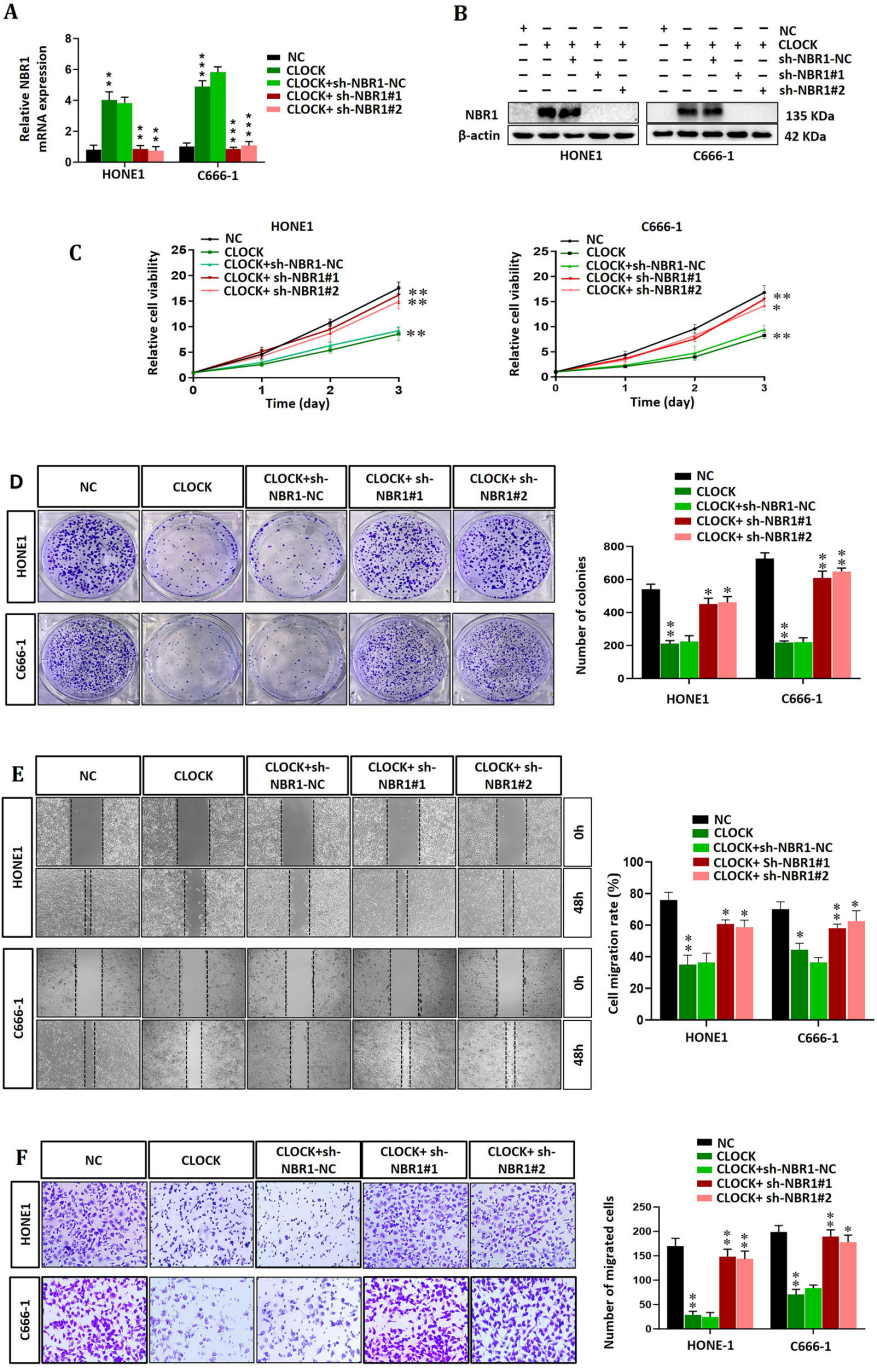
NBR1 mediates CLOCK to inhibit NPC cell proliferation and migration. **A**, **B** Efficient knockdown of NBR1 was confirmed by qRT-PCR and Western blot analyses using two independent shRNAs (sh-NBR1#1 and sh-NBR1#2) in CLOCK-overexpressing HONE1 and C666-1 cells. **C** The CCK-8 assay was performed to assess cell viability in CLOCK-overexpressing HONE1 and C666-1 cells following NBR1 knockdown at days 0, 1, 2, and 3. **D** Colony formation assays were conducted to evaluate clonogenic capacity of CLOCK-overexpressing cells after NBR1 knockdown, with colonies fixed, stained, and counted after 15–20 days of culture. **E**, **F** Wound healing and transwell migration assays were performed to assess migratory ability of CLOCK-overexpressing HONE1 and C666-1 cells after NBR1 knockdown. Data are presented as mean ± SD from three independent experiments. *, **, and *** indicate *p* < 0.05, *p* < 0.01, and *p* < 0.001, respectively.

To determine whether NBR1 mediates the anti-tumor activity of CLOCK, we utilized HONE1 and C666-1 cells stably overexpressing CLOCK, and further transduced them with control shRNA or two independent shRNAs targeting NBR1 (sh-NBR1#1 and sh-NBR1#2), generating five experimental groups (NC, CLOCK, CLOCK+sh-NBR1-NC, CLOCK+sh-NBR1#1, CLOCK+sh-NBR1#2). Efficient knockdown of NBR1 in CLOCK-overexpressing cells was confirmed by qRT-PCR and Western blot analyses (Fig. 5A, B). Functionally, CLOCK overexpression significantly suppressed cell viability and clonogenic growth in both HONE1 and C666-1 cells, as assessed by CCK-8 and colony formation assays (Fig. 5C, D). Notably, NBR1 knockdown in CLOCK-overexpressing cells significantly rescued these phenotypes, restoring proliferation and clonogenic growth to levels comparable to those of control cells. Furthermore, wound healing and Transwell migration assays revealed that the inhibitory effect of CLOCK on NPC cell motility was reversed upon NBR1 silencing, resulting in enhanced wound closure and increased migratory cell numbers (Fig. 5E, F).

To further investigate the role of NBR1 under physiological conditions, we repeated the NBR1 knockdown experiments in CNE1 cells, which have high endogenous CLOCK expression. This approach allowed us to assess whether the CLOCK–NBR1 relationship holds in cells with physiological levels of CLOCK, complementing the results obtained in CLOCK-overexpressing HONE1 and C666-1 cells. Efficient knockdown of NBR1 in CNE1 cells was confirmed by qRT-PCR and Western blot (Supplementary Fig. S2A). Similar to the results observed in HONE1 and C666-1 cells, NBR1 knockdown in CNE1 cells promoted cell proliferation and migration, as assessed by CCK-8, colony formation assays, and wound healing and Transwell migration assays (Supplementary Fig. S2B–E).

Loss-of-function analyses revealed that NBR1 is indispensable for CLOCK-mediated suppression of NPC cell proliferation and migration, positioning NBR1 as a key downstream effector. These results underscore the functional importance of the CLOCK–NBR1 axis in NPC tumor suppression.

### CLOCK promotes NBR1-mediated p62 accumulation and LLPS formation in NPC cells

To identify NBR1-interacting partners potentially involved in CLOCK-mediated tumor suppression, we performed a protein-protein interaction (PPI) network analysis by integrating data from four independent databases: BioGRID, GeneMANIA, STRING, and the Human Protein Atlas. Cross-comparison of these datasets revealed six common candidate proteins (Supplementary Fig. S3A, B), among which p62/SQSTM1 was identified. Given the well-established interaction between p62 and NBR1 across multiple cellular contexts [18, 31, 32], p62 was selected for further investigation as a representative candidate to explore the functional consequences of the CLOCK-NBR1 axis in NPC cells.

**Fig. 6:**
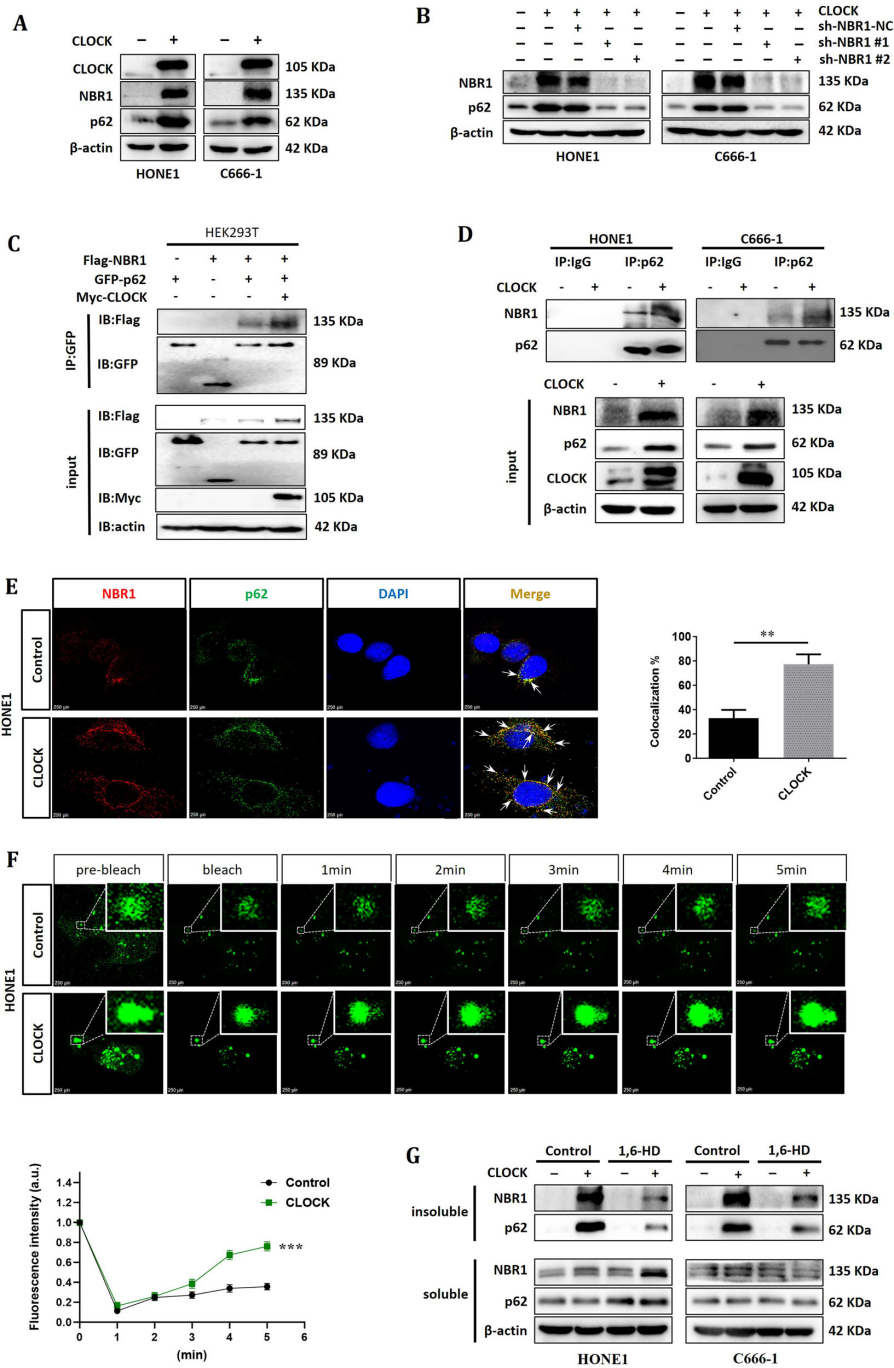
CLOCK promotes NBR1/p62 LLPS formation. Western blot analyses were performed to examine p62 protein expression in HONE1 and C666-1 cells with or without CLOCK overexpression (**A**) and following NBR1 knockdown using two independent shRNAs (sh-NBR1#1 and sh-NBR1#2) (**B**). Co-IP assays were conducted to assess the interaction between NBR1 and p62 in HEK293T cells (**C**) and in NPC cells (HONE1 and C666-1) (**D**). **E** Immunofluorescence staining of NBR1 and p62, together with DAPI nuclear counterstaining, was performed to visualize puncta formation in NPC cells. **F** HONE1 cells were co-transfected with either plvx-CLOCK or the plvx empty vector together with Flag-NBR1 and GFP-p62. Live-cell FRAP analysis was performed 48 h post-transfection using a Leica TCS SP8 confocal microscope. GFP fluorescence was photobleached with a 488-nm laser, and fluorescence recovery was monitored for 300 s. Representative recovery curves and quantification of the half-time of fluorescence recovery (t½) are shown. **G** Detergent-soluble and detergent-insoluble protein fractions were prepared from HONE1 and C666-1 cells under four conditions: NC, CLOCK overexpression, NC treated with 5% 1,6-HD, and CLOCK overexpression treated with 5% 1,6-HD for 5 min. Western blot analysis was performed to detect NBR1 and p62. Data are presented as mean ± SD from three independent experiments. **, and *** indicate *p* < 0.01, and *p* < 0.001, respectively. Co-IP Co-immunoprecipitation, FRAP fluorescence recovery after photobleaching, 1,6-HD 1,6-hexanediol.

We first examined whether CLOCK regulates p62 expression. qRT-PCR analysis revealed no significant changes in p62 mRNA levels upon CLOCK overexpression (Supplementary Fig. S3C), indicating that CLOCK does not transcriptionally regulate p62. In contrast, Western blot analysis demonstrated that CLOCK overexpression markedly increased p62 protein abundance in parallel with elevated NBR1 levels in both HONE1 and C666-1 cells (Fig. 6A). Based on previous reports showing that NBR1 stabilizes p62 by preventing its selective autophagic degradation [32], we hypothesized that CLOCK-induced p62 accumulation is mediated by NBR1. Consistently, knockdown of NBR1 using two independent shRNAs (sh-NBR1#1 and sh-NBR1#2) completely abolished the CLOCK-induced increase in p62 protein levels (Fig. 6B), supporting a model in which CLOCK transcriptionally upregulates NBR1, thereby stabilizing p62.

To validate the interaction between NBR1 and p62, Co-IP assays were performed. In HEK293T cells co-transfected with FLAG-NBR1, GFP-p62, and MYC-CLOCK, the presence of CLOCK markedly enhanced the interaction between NBR1 and p62 (Fig. 6C). Endogenous Co-IP assays in both HONE1 and C666-1 cells further confirmed this interaction (Fig. 6D), reinforcing the functional relevance of the CLOCK-NBR1-p62 axis in NPC cells.

Previous studies have reported that NBR1 and p62 can undergo LLPS to form cytoplasmic puncta [18, 32, 33]. To explore whether the CLOCK-NBR1-p62 interaction similarly influences LLPS, we next investigated this phenomenon. Immunofluorescence analysis revealed co-localization of NBR1 and p62 within cytoplasmic puncta in cells co-expressing NBR1 and p62, which was significantly enhanced upon CLOCK overexpression (Fig. 6E). Live-cell fluorescence recovery after photobleaching (FRAP) analysis in HONE1 cells co-transfected with plvx-CLOCK or empty vector together with FLAG-NBR1 and GFP-p62 demonstrated significantly accelerated fluorescence recovery in CLOCK-overexpressing cells following photobleaching with a 488-nm laser, fluorescence recovery was monitored for 300 s (Fig. 6F). Quantitative analysis revealed a significant reduction in the half-time of fluorescence recovery (t½) and increased molecular mobility within NBR1-p62 puncta, whereas control cells exhibited slower and incomplete recovery. In addition, CLOCK overexpression increased both the number and fluorescence intensity of NBR1-p62 puncta, indicating enhanced LLPS formation. To further substantiate these observations, biochemical fractionation was performed to separate detergent-soluble and detergent-insoluble (LLPS-enriched) fractions from HONE1 and C666-1 cells under four experimental conditions: negative control, CLOCK overexpression, negative control treated with 5% 1,6-Hexanediol (1,6-HD), and CLOCK overexpression treated with 5% 1,6-HD for 5 min. Western blot analysis revealed a marked enrichment of NBR1 and p62 in the detergent-insoluble fraction upon CLOCK overexpression. Notably, treatment with 1,6-HD substantially reduced the accumulation of NBR1 and p62 in the insoluble fraction of CLOCK-overexpressing cells, while their levels in the soluble fraction remained largely unchanged (Fig. 6G). These findings indicate that the condensates induced by CLOCK overexpression are LLPS-dependent. Importantly, consistent results were observed in both NPC cell lines, underscoring the robustness of CLOCK-mediated LLPS regulation.

At the mechanistic level, transcriptional activation of NBR1 by CLOCK led to its cytoplasmic accumulation, which stabilized p62 by preventing its selective autophagic degradation. The simultaneous accumulation of NBR1 and p62 promoted the formation of NBR1-p62 complexes and facilitated LLPS condensate assembly, revealing a phase separation-dependent mechanism that underlies CLOCK-mediated tumor suppression in NPC cells.

### CLOCK-NBR1/p62-TRIM38 axis mediates TAB2 degradation via LLPS-dependent ubiquitination

**Fig. 7:**
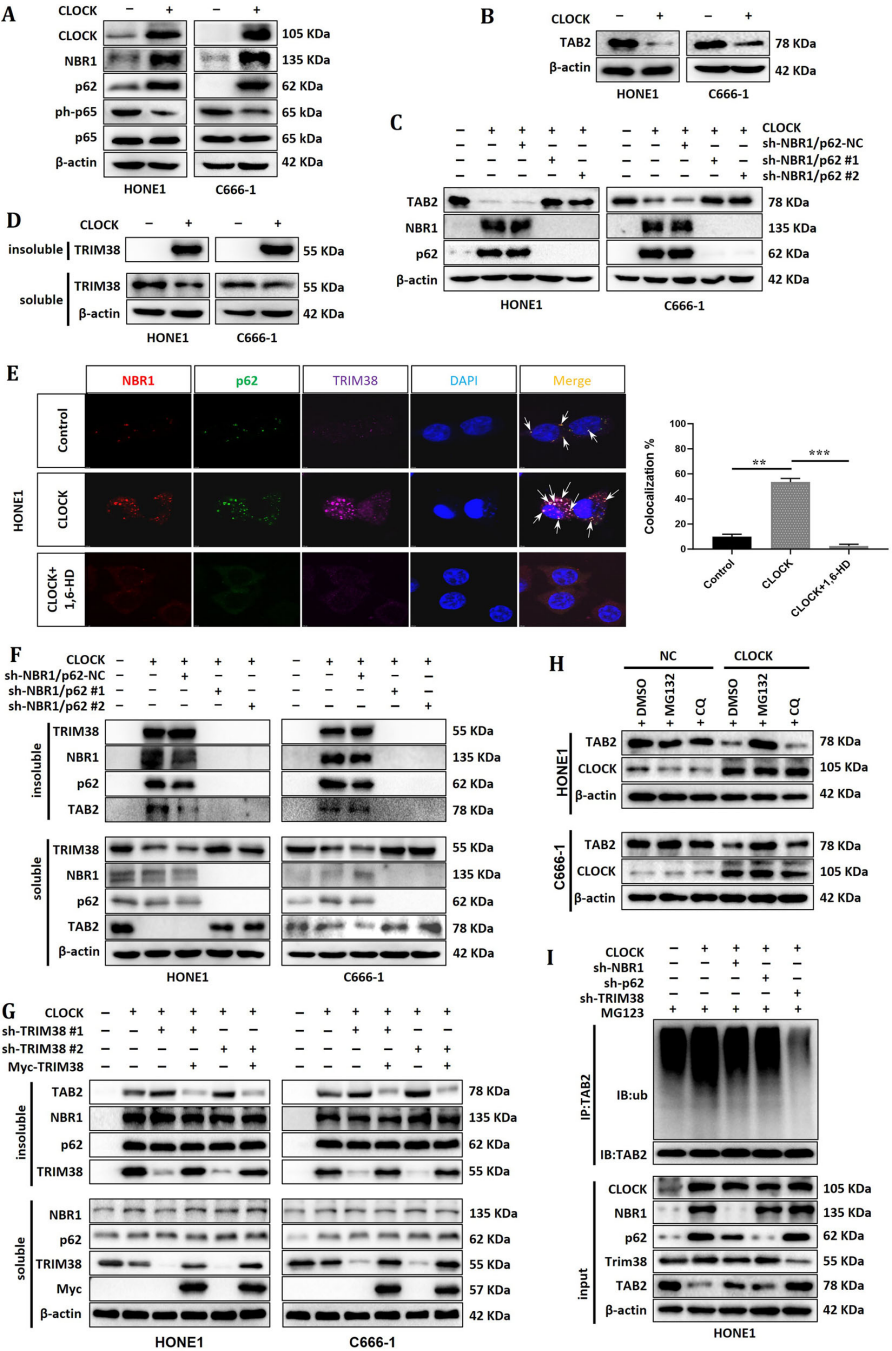
TRIM38 is involved in NBR1/p62 LLPs to mediate TAB2 degradation. **A–C** Western blot analyses were performed to assess phosphorylated p65 and total p65 (**A**) and TAB2 protein levels (**B**) in HONE1 and C666-1 cells with or without CLOCK overexpression. TAB2 expression was further examined following NBR1 and p62 knockdown using two independent shRNAs (sh-NBR1/p62#1 and sh-NBR1/p62#2) in the same cell lines (**C**)**. D** Detergent-soluble and detergent-insoluble protein fractions were analyzed by Western blot to examine TRIM38 levels in HONE1 and C666-1 cells with or without CLOCK overexpression. **E** Immunofluorescence staining was performed to visualize NBR1, p62, and TRIM38 puncta formation, with DAPI used for nuclear counterstaining. HONE1 cells were co-transfected with Flag-NBR1, GFP-p62, and Myc-TRIM38 together with plvx empty vector or plvx-CLOCK. To disrupt phase separation, a subset of CLOCK-overexpressing cells was treated with 5% 1,6-HD for 5 min. Representative images illustrate puncta formation under each condition. **F**, **G** Detergent-soluble and detergent-insoluble protein fractions were analyzed by Western blot. **F** TRIM38, NBR1, p62, and TAB2 protein levels were examined following NBR1/p62 knockdown. **G** TAB2, TRIM38, NBR1, and p62 protein levels were assessed following TRIM38 knockdown using two independent shRNAs (sh-TRIM38#1 and sh-TRIM38#2), with subsequent rescue by ectopic expression of Myc-TRIM38. **H** TAB2 protein levels were analyzed by Western blot in HONE1 and C666-1 cells transfected with CLOCK or empty vector and treated with 20 μM MG132 or 30 μM CQ prior to harvesting. **I** HONE1 cells were transfected with the indicated plasmids and treated with MG132 (20 μM) for 8 h prior to harvesting. Cell lysates were immunoprecipitated with anti-TAB2 antibody, and ubiquitinated proteins were captured using protein A/G magnetic beads. Immunoprecipitated proteins were analyzed by Western blotting with the indicated antibodies. Data are presented as mean ± SD from three independent experiments. **, and *** indicate *p* < 0.01, and *p* < 0.001, respectively. 1,6-HD 1,6-Hexanediol, CQ chloroquine.

Building on our finding that CLOCK promotes NBR1-p62-dependent LLPS assembly, we next investigated the downstream signaling consequences of this condensate formation. Given the well-established role of NBR1 and p62 in regulating inflammatory responses, we focused on the NF-κB pathway, specifically assessing the canonical subunit p65 and its phosphorylated, activated form (ph-p65). In both HONE1 and C666-1 cells, total p65 levels remained unchanged between CLOCK-overexpressing and control groups; however, ph-p65 levels were markedly reduced upon CLOCK overexpression **(**Fig. 7A), indicating that CLOCK suppresses NF-κB signaling activity. To identify upstream mediators of this suppression, we examined TAB2, a critical adaptor involved in TGF-β–induced NF-κB activation. Western blot analysis revealed that TAB2 expression was significantly reduced in CLOCK-overexpressing cells compared with controls in both NPC cell lines (Fig. 7B). To determine whether this regulation was mediated via NBR1 and p62, we performed simultaneous knockdown of NBR1 and p62 using two independent shRNA constructs in the context of CLOCK overexpression (Fig. 7C). While CLOCK overexpression alone suppressed TAB2 expression, concurrent depletion of NBR1 and p62 restored TAB2 levels to those of control cells, confirming that CLOCK-mediated TAB2 downregulation is dependent on NBR1 and p62. Additional supporting data, including the expression of NBR1, p62, and TAB2 across NPC cell lines as well as the normal nasopharyngeal epithelial NP69 cells, are presented in Supplementary Fig. S4.

To identify potential effectors linking NBR1/p62 condensates to TAB2 degradation, we interrogated the STRING database for protein-protein interaction (PPI) networks of NBR1 and p62 and identified overlapping interactors (Supplementary Fig. S5A). This analysis revealed 136 proteins common to both interactomes (25.8% overlap), including two E3 ubiquitin ligases, KEAP1 and STUB1, both known to mediate ubiquitin-dependent protein degradation. Western blotting of soluble and insoluble protein fractions (Supplementary Fig. S5B) showed no significant changes in KEAP1 or STUB1 expression upon CLOCK overexpression, suggesting that these ligases are not responsible for CLOCK-induced TAB2 degradation. Given these findings, we considered TRIM family E3 ligases, which are known to localize to LLPS condensates. BioGRID analysis of p62 interactors revealed several TRIM proteins, with TRIM21 displaying the most robust interaction network (Supplementary Fig. S6A). However, Western blotting of soluble and insoluble fractions showed no alteration in TRIM21 levels upon CLOCK induction (Supplementary Fig. S6B), ruling out its involvement.

We then focused on TRIM38, an E3 ligase previously reported to target TAB2 for ubiquitin-mediated degradation and suppress NF-κB signaling in both cancer and non-cancer systems [34–36]. Strikingly, CLOCK overexpression markedly increased TRIM38 abundance in the insoluble fraction of both NPC cell lines, whereas TRIM38 was barely detectable in control cells (Fig. 7D). This pattern suggested that CLOCK promotes TRIM38 sequestration into NBR1/p62 condensates. To visualize the formation of NBR1, p62, and TRIM38 puncta, immunofluorescence staining was performed in HONE1 cells co-transfected with Flag-NBR1, GFP-p62, and Myc-TRIM38, together with plvx-empty vector or plvx-CLOCK. In the control group (plvx-empty vector), NBR1, p62, and TRIM38 puncta were observed at minimal intensity. In contrast, CLOCK-overexpressing control (plvx-CLOCK) exhibited prominent, large puncta of NBR1, p62, and TRIM38, indicative of strong phase separation. Notably, treatment with 5% 1,6-HD for 5 min to disrupt phase separation almost completely abolished puncta formation, demonstrating that CLOCK-induced LLPS is dependent on phase separation dynamics and can be disrupted by 1,6-HD treatment (Fig. 7E).

To assess whether TRIM38 recruitment depends on NBR1 and p62, fractionation assays were performed following double knockdown of NBR1 and p62. CLOCK overexpression alone drove NBR1, p62, and TRIM38 into the insoluble fraction in both HONE1 and C666-1 cells; however, simultaneous depletion of NBR1 and p62 abolished TRIM38 insoluble localization without significantly affecting its soluble pool (Fig. 7F). These findings indicate that TRIM38 recruitment into condensates is specifically dependent on NBR1/p62 scaffolding. Consistent with this notion, analysis of the same fractionated samples further revealed that CLOCK overexpression markedly decreased TAB2 levels in the soluble fraction, while promoting its redistribution to the insoluble fraction. Importantly, knockdown of NBR1/p62 restored TAB2 abundance in the soluble fraction and reduced its insoluble pool, indicating that TAB2 degradation is dependent on NBR1/p62-driven condensate formation.

To determine whether TRIM38 is required for CLOCK-induced TAB2 degradation, we silenced TRIM38 using two independent shRNAs (Supplementary Fig. S7) and examined TAB2 levels in the insoluble fraction under CLOCK overexpression. CLOCK overexpression reduced insoluble TAB2, consistent with enhanced sequestration and potential degradation via TRIM38. Knockdown of TRIM38 substantially restored TAB2 levels, whereas re-expression of Myc-TRIM38 reinstated TAB2 reduction. Notably, NBR1 and p62 levels remained unchanged across all conditions, indicating that TRIM38 functions downstream of the CLOCK–NBR1/p62 LLPS platform (Fig. 7G). To determine the degradation pathway responsible for CLOCK-dependent TAB2 loss, HONE1 and C666-1 cells were treated with the proteasome inhibitor MG132 or the lysosomal inhibitor chloroquine (CQ) in the presence or absence of CLOCK overexpression. MG132 markedly restored TAB2 protein abundance in CLOCK-overexpressing cells, whereas CQ failed to rescue TAB2 levels compared with DMSO controls. CLOCK expression itself remained unaffected by either inhibitor, indicating that TAB2 degradation occurs predominantly via the ubiquitin–proteasome pathway rather than the lysosome (Fig. 7H).

Building on this, we next asked whether TRIM38 mediates TAB2 ubiquitination downstream of CLOCK-induced NBR1/p62 LLPS. An in vivo ubiquitination assay was performed in HONE1 cells transfected with the indicated plasmids and treated with MG132 (20 μM) for 8 h to prevent degradation of ubiquitinated proteins. Lysates were immunoprecipitated with an anti-TAB2 antibody, followed by Western blot analysis to detect ubiquitinated TAB2. In basal control cells lacking CLOCK overexpression, low levels of TAB2 ubiquitination were observed. CLOCK overexpression alone markedly increased TAB2 ubiquitination, whereas depletion of NBR1 or p62 in CLOCK-overexpressing cells substantially reduced ubiquitination, indicating that both NBR1 and p62 are required for this process. Strikingly, knockdown of TRIM38 almost completely abolished TAB2 ubiquitination despite sustained CLOCK overexpression, demonstrating that TRIM38 is indispensable for CLOCK-induced TAB2 ubiquitination (Fig. 7I).

Building on this LLPS-dependent framework, CLOCK suppresses NF-κB signaling through a defined molecular cascade. CLOCK-induced NBR1, together with stabilized p62, promoted condensate formation that recruited TRIM38, facilitating proteasome-mediated degradation of TAB2. This cascade attenuated NF-κB activation, thereby reducing NPC cell proliferation and migration and establishing a CLOCK–NBR1/p62–TRIM38–TAB2 tumor-suppressive axis.

### CLOCK inhibits NPC tumor growth in vivo

**Fig. 8:**
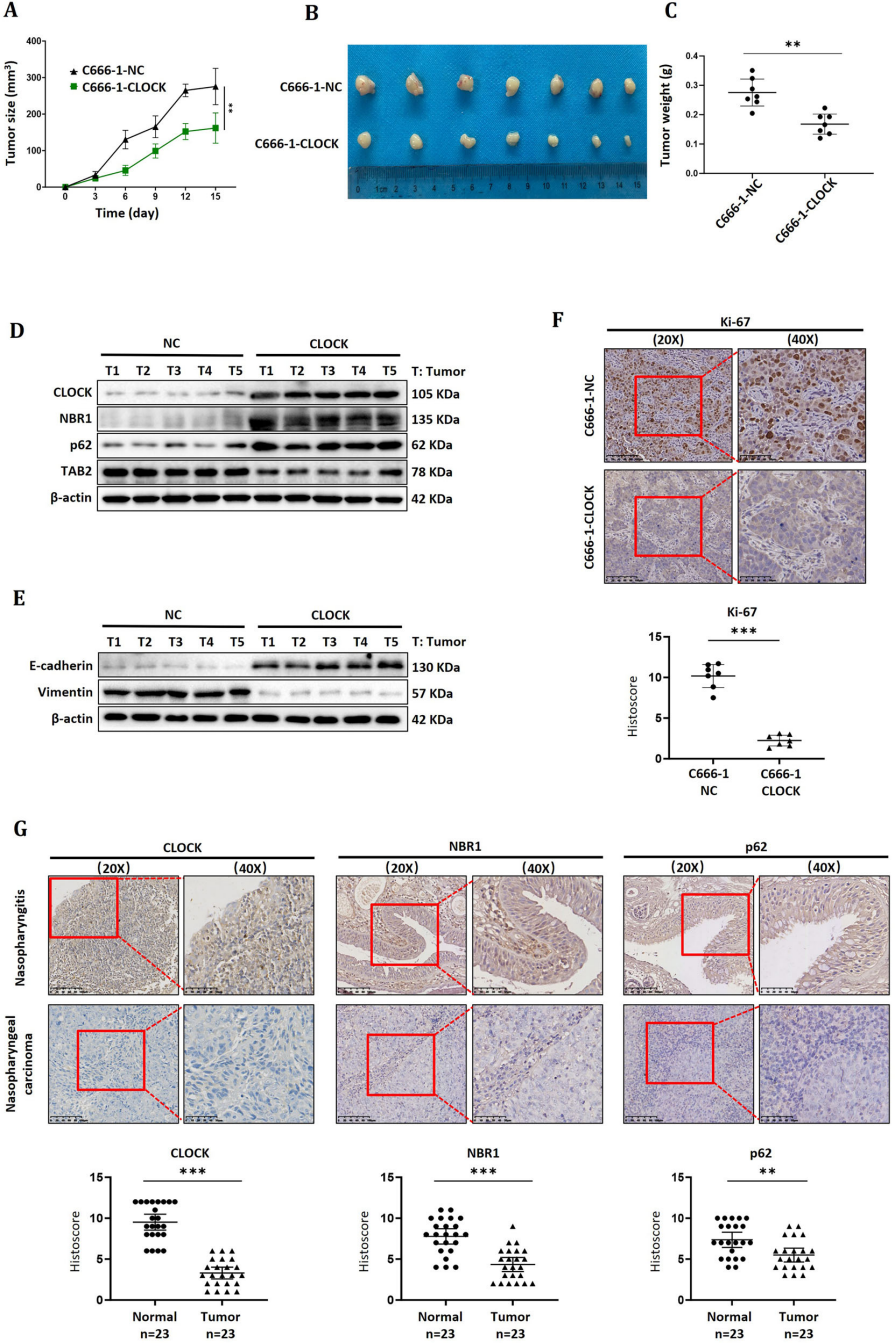
CLOCK inhibits NPC growth in vivo. **A** Tumor volume growth curves were generated for nude mice subcutaneously injected with C666-1-NC or C666-1-CLOCK cells (*n* = 7 per group). **B** Representative images of subcutaneous xenograft tumors. **C** Tumor weights measured at the experimental endpoint from xenografts derived from C666-1-NC and C666-1-CLOCK cells (*n* = 7 per group). Western blot analysis was performed to evaluate the expression of CLOCK, NBR1, p62, and TAB2 (**D**), as well as the EMT markers E-cadherin and Vimentin (**E**), in xenograft tumor tissues. **F** Representative immunohistochemical (IHC) staining of Ki-67 in murine xenograft tumor sections. **G** Representative IHC staining of CLOCK, NBR1, and p62 in human nasopharyngeal epithelial tissues (*n* = 23) and nasopharyngeal carcinoma tissues (*n* = 23). Data are presented as mean ± SD from three independent experiments. ** and *** indicate *p* < 0.01 and *p* < 0.001, respectively.

To validate our in vitro findings in an in vivo context, we employed a subcutaneous xenograft model in immunodeficient mice. C666-1 cells stably transfected with either an empty vector (negative control, NC) or a CLOCK-overexpression construct were injected subcutaneously into the flanks of mice (*n* = 7 per group). Tumor growth was monitored daily, and tumor volumes were calculated throughout the experimental period. At day 15, mice were sacrificed, and tumors were excised, photographed, and weighed. CLOCK-overexpressing tumors exhibited significantly slower growth rates, reduced tumor volumes, and lower tumor weights compared with NC tumors (Fig. 8A–C). To investigate molecular alterations associated with CLOCK overexpression in vivo, Western blot analysis was performed on xenograft tumor lysates (*n* = 5 per group). CLOCK overexpression markedly increased NBR1 and p62 protein levels, while concomitantly reducing TAB2 abundance relative to NC tumors (Fig. 8D). Consistent with the in vitro anti-migratory effects, CLOCK-overexpressing tumors displayed increased E-cadherin and decreased vimentin expression, indicating suppression of epithelial-mesenchymal transition (EMT) in vivo (Fig. 8E). Functional relevance was further evaluated in xenograft models (*n* = 7 per group) using Ki-67 immunohistochemical (IHC) staining. CLOCK-overexpressing tumors exhibited a significantly reduced proliferative index compared with NC tumors, confirming the growth-inhibitory effect of CLOCK in vivo (Fig. 8F). To assess clinical relevance, IHC analysis was performed on human nasopharyngeal tissue specimens, including 23 NPC tissues (Supplementary Table S3) and 23 normal nasopharyngeal tissues (Supplementary Table S4). Histoscore analysis revealed that CLOCK expression was significantly lower in NPC tissues than in normal nasopharyngeal epithelium. Consistent with the proposed CLOCK–NBR1/p62 axis, the expression levels of both NBR1 and p62 were also markedly reduced in NPC specimens compared with normal tissues (Fig. 8G), supporting the clinical relevance of CLOCK-mediated regulation of the NBR1/p62 pathway in NPC.

Consistent with in vitro observations, in vivo analyses confirmed the tumor-suppressive role of CLOCK in NPC. Overexpression of CLOCK restrained tumor growth, suppressed EMT, and reduced proliferative activity, whereas human NPC tissues exhibited concomitant reductions in CLOCK, NBR1, and p62 levels, further highlighting the clinical relevance of this CLOCK-centered regulatory network.

## Discussion

This study provides the first comprehensive evidence that CLOCK functions as a tumor suppressor in NPC, challenging the prevailing view from other cancers in which CLOCK acts as an oncogenic driver. In various malignancies, including glioblastoma, hepatocellular carcinoma, and colorectal cancer, CLOCK overexpression has been associated with enhanced proliferation, migration, and therapy resistance [6, 8–15]. This context-dependent behavior likely reflects the role of CLOCK as a transcription factor operating within cell type-specific chromatin landscapes and in concert with distinct cofactors. In NPC, CLOCK preferentially activates tumor-suppressive pathways, including NBR1-dependent LLPS formation and inhibition of NF-κB signaling, whereas in other cancer types it drives oncogenic transcriptional programs that promote proliferation, migration, and therapy resistance [6]. This functional duality appears to be shaped by tissue-specific transcriptional networks and microenvironmental cues, such as inflammation, viral infection, and immune cell composition, which collectively modulate downstream effects of CLOCK. Such context-dependent behavior aligns with observations for other transcription factors, many of which function as tumor suppressors in certain cancers but act as oncogenes in others depending on chromatin accessibility, cofactor availability, and signaling context [37, 38].

To date, only two isolated studies, one in ALDH-positive 4T1 mouse breast cancer cells and another in LS180 colon cancer cells, have reported CLOCK downregulation in specific contexts [28, 39]. However, these studies were limited by their use of murine or single-cell-line models, lacked mechanistic analyses, and provided no direct evidence linking CLOCK to tumor suppression in human cancers. In contrast, our study employs human NPC cell lines and an integrative approach, spanning phenotypic characterization to mechanistic interrogation, to demonstrate the tumor-suppressive role of CLOCK. Overexpression of CLOCK markedly impaired NPC cell proliferation and migration, whereas silencing endogenous CLOCK enhanced these oncogenic phenotypes. Importantly, re-expression of CLOCK in knockdown cells reversed these effects, confirming the specificity of CLOCK-mediated regulation. Collectively, these bidirectional genetic experiments provide strong evidence that CLOCK directly restrains NPC cell growth and motility. Notably, this study provides the first mechanistic insight into how CLOCK acts as a tumor suppressor in NPC, revealing a pathway by which a core circadian regulator modulates LLPS-mediated signaling to dynamically control cancer cell behavior.

**Fig. 9:**
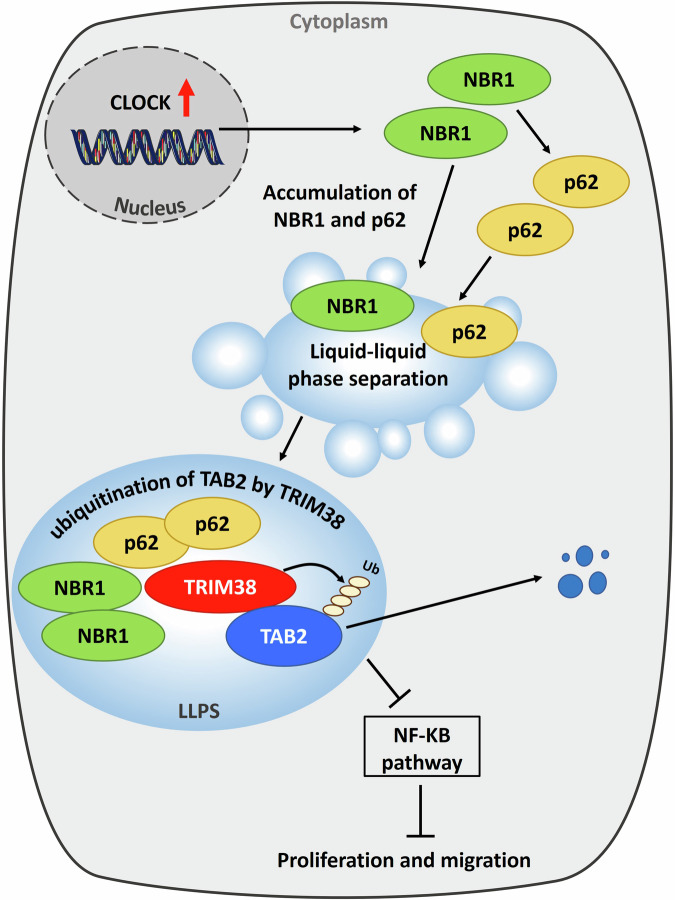
Schematic model of CLOCK-mediated LLPS signaling axis in NPC. CLOCK transcriptionally upregulates NBR1, leading to cytoplasmic accumulation of NBR1 and stabilization of p62/SQSTM1. The cooperative interaction between NBR1 and p62 drives the formation of LLPS condensates, which serve as scaffolds for the recruitment of TRIM38. TRIM38 catalyzes ubiquitination of TAB2, promoting its proteasomal degradation. TAB2 degradation attenuates NF-κB pathway activation, resulting in inhibition of NPC cell proliferation and migration.

Mechanistically, we identified NBR1 as a transcriptional target of CLOCK. Overexpression of CLOCK increased cytoplasmic NBR1 protein abundance, which in turn stabilized p62/SQSTM1 and promoted their co-assembly into LLPS condensates. These NBR1-p62 droplets form specialized signaling platforms that recruit the E3 ubiquitin ligase TRIM38, facilitating ubiquitination and subsequent proteasomal degradation of TAB2. Given TAB2’s role as a key scaffold protein in NF-κB signaling, its degradation suppresses pathway activation. A schematic working model is presented in Fig. 9, illustrating how CLOCK transcriptionally activates NBR1, stabilizes p62, and promotes condensate formation, ultimately leading to TRIM38-mediated TAB2 degradation and NF-κB suppression. Because NF-κB is a master regulator of inflammatory signaling, cell survival, and oncogenic progression, its suppression provides a mechanistic explanation for the observed inhibition of NPC cell proliferation and migration.

The identification of LLPS as a pivotal intermediate in CLOCK-mediated tumor suppression is particularly noteworthy. LLPS enables the spatiotemporal compartmentalization of signaling molecules, allowing dynamic regulation of diverse cellular processes [40]. Among the best-characterized LLPS drivers are NBR1 and its binding partner p62/SQSTM1, both functioning as selective autophagy receptors. Biochemical and cell-based evidence supports a role for NBR1 in facilitating ubiquitin-positive phase-separated droplet formation by p62 through high-affinity ubiquitin-binding domains, including UBA and PB1, that are indispensable for LLPS assembly [18, 33]. Notably, elevated cytoplasmic NBR1 has been reported to stabilize p62 and prevent its degradation, thereby sustaining the availability of both proteins for droplet formation [32]. In cancer, Noguchi et al. demonstrated that lysosomal stress–induced p62/NBR1 condensates recruit degradation machinery, limit Rac1-driven motility, and suppress metastatic potential [41]. Building on these foundations, our study shows that CLOCK-driven cytoplasmic NBR1 elevation stabilizes p62, promoting LLPS condensate formation with potent tumor-suppressive properties. This expands the functional scope of CLOCK beyond its canonical circadian role and positions it as a key regulator of phase-separation–dependent signaling in cancer.

Members of the TRIM (tripartite motif-containing) protein family are increasingly recognized for their ability to partition into and, in some cases, initiate LLPS structures through intrinsically disordered regions, coiled-coil domains, and PRY/SPRY motifs, thereby spatially concentrating E3 ubiquitin ligase activity [42, 43]. For instance, TRIM32, TRIM5α, and TRIM63 have been shown to self-organize into cytoplasmic bodies through LLPS in a ubiquitination-dependent manner, providing a mechanistic precedent for LLPS-driven compartmentalization within the TRIM family [42]. Our data identify TRIM38 as a critical effector incorporated into pre-assembled NBR1-p62 condensates, without being required for their initiation. Within these condensates, the high local density of ubiquitin-binding domains facilitates efficient enzymatic function. This recruitment-driven activity parallels previous observations for TRIM21, which localizes to stress granule LLPS assemblies formed by G3BP1, catalyzing K63-linked ubiquitination to regulate granule dynamics and autophagy-dependent clearance [44]. Analogously, our data demonstrate that TRIM38 is incorporated into NBR1-p62 condensates, where it mediates ubiquitination and promotes the proteasomal degradation of TAB2, consistent with mechanisms previously reported in inflammatory and cancer-related contexts [34–36]. Collectively, these findings establish a mechanistic basis by which TRIM38 recruitment into NBR1-p62 LLPS droplets sustains TAB2 turnover and suppresses NF-κB signaling in NPC, thereby integrating LLPS biology with ubiquitin-mediated proteolysis and highlighting the broader potential of targeting LLPS-resident E3 ligases in cancer therapy.

Beyond CLOCK in NPC, these findings suggest a broader conceptual framework in which circadian regulators intersect with LLPS biology to shape cancer signaling networks. Circadian factors, traditionally associated with systemic metabolic control and transcriptional regulation, can directly modulate protein stability and ubiquitin-dependent signaling via biomolecular condensates. This duality may explain CLOCK’s divergent roles across malignancies, where oncogenic or tumor-suppressive outcomes may emerge from differential engagement of LLPS-mediated pathways.

From a translational perspective, these insights offer new avenues for therapeutic intervention. Approaches that stabilize tumor-suppressive condensates or selectively promote recruitment of E3 ligases such as TRIM38 may represent novel strategies to attenuate pro-tumorigenic NF-κB signaling. Thus, the mechanistic link uncovered between CLOCK, LLPS, and ubiquitin-mediated proteolysis not only advances our understanding of circadian gene contributions to cancer biology but also establishes a conceptual bridge toward condensate-centered therapeutic strategies in NPC and potentially other malignancies.

## Conclusion

Our study establishes, for the first time, that CLOCK functions as a tumor suppressor in NPC by transcriptionally upregulating NBR1. Elevated cytoplasmic NBR1 stabilizes p62 and prevents its degradation, thereby maintaining both proteins for cooperative assembly. NBR1 and p62 together form LLPS condensates that recruit TRIM38, which mediates ubiquitination and proteasomal degradation of TAB2, ultimately suppressing NF-κB signaling and restraining NPC cell proliferation and migration. This CLOCK–NBR1/p62–TRIM38–TAB2–NF-κB axis represents a novel mechanism of CLOCK-mediated tumor suppression, integrating transcriptional control with phase separation dynamics and proteasomal targeting. These findings not only redefine the biological role of CLOCK in cancer but also suggest potential therapeutic avenues that exploit CLOCK-dependent phase separation to restrain oncogenic inflammation.

## Supplementary information

WB original data PDF.pdf

Supplementary materials and methods.docx

Supplementary Figure S1.jpg

Supplementary Figure S2.jpg

Supplementary Figure S3.jpg

Supplementary Figure S4.jpg

Supplementary Figure S5.jpg

Supplementary Figure S6.jpg

Supplementary Figure S7.jpg

Supplementary Tables 1-4.xlsx

## Data Availability

The datasets generated and/or analyzed during the current study are included in this published article and its supplementary information files.

## References

[1] Liu Q, Wang H, Chen Z, Xiong J, Huang Y, Zhang S, et al. Global, regional, and national epidemiology of nasopharyngeal carcinoma in middle-aged and elderly patients from 1990 to 2021. Ageing Res Rev. 2025;104:102613. 39626854 10.1016/j.arr.2024.102613

[2] Chen YP, Chan ATC, Le QT, Blanchard P, Sun Y, Ma J. Nasopharyngeal carcinoma. Lancet. 2019;394:64–80. 31178151 10.1016/S0140-6736(19)30956-0

[3] Tang LL, Guo R, Zhang N, Deng B, Chen L, Cheng ZB, et al. Effect of radiotherapy alone vs radiotherapy with concurrent chemoradiotherapy on survival without disease relapse in patients with low-risk nasopharyngeal carcinoma: a randomized clinical trial. JAMA. 2022;328:728–36. 35997729 10.1001/jama.2022.13997 PMC9399866

[4] Janoski JR, Aiello I, Lundberg CW, Finkielstein CV. Circadian clock gene polymorphisms implicated in human pathologies. Trends Genet. 2024;40:834–52. 38871615 10.1016/j.tig.2024.05.006

[5] Fan XL, Song Y, Qin DX, Lin PY. Regulatory effects of clock and Bmal1 on circadian rhythmic TLR expression. Int Rev Immunol. 2023;42:101–12. 34544330 10.1080/08830185.2021.1931170

[6] Rashed N, Liu W, Zhou X, Bode AM, Luo X. The role of circadian gene CLOCK in cancer. Biochim Biophys Acta Mol Cell Res. 2024;1871:119782. 38871225 10.1016/j.bbamcr.2024.119782

[7] Takahashi JS, Shimomura K, Kumar V. Searching for genes underlying behavior: lessons from circadian rhythms. Science. 2008;322:909–12. 18988844 10.1126/science.1158822 PMC3744585

[8] Liu T, Wang Z, Ye L, Duan Y, Jiang H, He H, et al. Nucleus-exported CLOCK acetylates PRPS to promote de novo nucleotide synthesis and liver tumour growth. Nat Cell Biol. 2023;25:273–84. 36646788 10.1038/s41556-022-01061-0

[9] Qu M, Zhang G, Qu H, Vu A, Wu R, Tsukamoto H, et al. Circadian regulator BMAL1::CLOCK promotes cell proliferation in hepatocellular carcinoma by controlling apoptosis and cell cycle. Proc Natl Acad Sci USA. 2023;120:e2214829120. 36595671 10.1073/pnas.2214829120 PMC9926257

[10] Dong Z, Zhang G, Qu M, Gimple RC, Wu Q, Qiu Z, et al. Targeting glioblastoma stem cells through disruption of the circadian clock. Cancer Discov. 2019;9:1556–73. 31455674 10.1158/2159-8290.CD-19-0215 PMC6983300

[11] Chen P, Hsu WH, Chang A, Tan Z, Lan Z, Zhou A, et al. Circadian regulator CLOCK recruits immune-suppressive microglia into the GBM tumor microenvironment. Cancer Discov. 2020;10:371–81. 31919052 10.1158/2159-8290.CD-19-0400 PMC7058515

[12] Xuan W, Hsu WH, Khan F, Dunterman M, Pang L, Wainwright DA, et al. Circadian regulator CLOCK drives immunosuppression in glioblastoma. Cancer Immunol Res. 2022;10:770–84. 35413115 10.1158/2326-6066.CIR-21-0559 PMC9177794

[13] Pang L, Dunterman M, Xuan W, Gonzalez A, Lin Y, Hsu WH, et al. Circadian regulator CLOCK promotes tumor angiogenesis in glioblastoma. Cell Rep. 2023;42:112127. 36795563 10.1016/j.celrep.2023.112127 PMC10423747

[14] He A, Huang Z, Zhang R, Lu H, Wang J, Cao J, et al. Circadian clock genes are correlated with prognosis and immune cell infiltration in colon adenocarcinoma. Comput Math Methods Med. 2022;2022:1709918. 35116071 10.1155/2022/1709918 PMC8807038

[15] Jiang P, Xu C, Zhang P, Ren J, Mageed F, Wu X, et al. Epigallocatechin‑3‑gallate inhibits self‑renewal ability of lung cancer stem‑like cells through inhibition of CLOCK. Int J Mol Med. 2020;46:2216–24. 33125096 10.3892/ijmm.2020.4758 PMC7595654

[16] Banani SF, Lee HO, Hyman AA, Rosen MK. Biomolecular condensates: organizers of cellular biochemistry. Nat Rev Mol Cell Biol. 2017;18:285–98. 28225081 10.1038/nrm.2017.7 PMC7434221

[17] Shin Y, Brangwynne CP. Liquid phase condensation in cell physiology and disease. Science. 2017;357:eaaf4382. 28935776 10.1126/science.aaf4382

[18] Turco E, Savova A, Gere F, Ferrari L, Romanov J, Schuschnig M, et al. Reconstitution defines the roles of p62, NBR1 and TAX1BP1 in ubiquitin condensate formation and autophagy initiation. Nat Commun. 2021;12:5212. 34471133 10.1038/s41467-021-25572-w PMC8410870

[19] Yan H, Qi A, Lu Z, You Z, Wang Z, Tang H, et al. Dual roles of AtNBR1 in regulating selective autophagy via liquid-liquid phase separation and recognition of non-ubiquitinated substrates in Arabidopsis. Autophagy. 2024;20:2804–15. 39162855 10.1080/15548627.2024.2391725 PMC11587852

[20] Sun D, Wu R, Zheng J, Li P, Yu L. Polyubiquitin chain-induced p62 phase separation drives autophagic cargo segregation. Cell Res. 2018;28:405–15. 29507397 10.1038/s41422-018-0017-7 PMC5939046

[21] Takaesu G, Kishida S, Hiyama A, Yamaguchi K, Shibuya H, Irie K, et al. TAB2, a novel adaptor protein, mediates activation of TAK1 MAPKKK by linking TAK1 to TRAF6 in the IL-1 signal transduction pathway. Mol Cell. 2000;5:649–58. 10882101 10.1016/s1097-2765(00)80244-0

[22] McKenzie M, Lian GY, Pennel KAF, Quinn JA, Jamieson NB, Edwards J. NFκB signalling in colorectal cancer: examining the central dogma of IKKα and IKKβ signalling. Heliyon. 2024;10:e32904. 38975078 10.1016/j.heliyon.2024.e32904 PMC11226910

[23] Hinz M, Scheidereit C. The IκB kinase complex in NF-κB regulation and beyond. EMBO Rep. 2014;15:46–61. 24375677 10.1002/embr.201337983 PMC4303448

[24] Mao H, Zhao X, Sun SC. NF-κB in inflammation and cancer. Cell Mol Immunol. 2025;22:811–39. 40562870 10.1038/s41423-025-01310-w PMC12310982

[25] Perkins ND. The diverse and complex roles of NF-κB subunits in cancer. Nat Rev Cancer. 2012;12:121–32. 22257950 10.1038/nrc3204

[26] Gu Z, Chen X, Yang W, Qi Y, Yu H, Wang X, et al. The SUMOylation of TAB2 mediated by TRIM60 inhibits MAPK/NF-κB activation and the innate immune response. Cell Mol Immunol. 2021;18:1981–94. 33184450 10.1038/s41423-020-00564-w PMC8322076

[27] Alhopuro P, Björklund M, Sammalkorpi H, Turunen M, Tuupanen S, Biström M, et al. Mutations in the circadian gene CLOCK in colorectal cancer. Mol Cancer Res. 2010;8:952–60. 20551151 10.1158/1541-7786.MCR-10-0086

[28] Ogino T, Matsunaga N, Tanaka T, Tanihara T, Terajima H, Yoshitane H, et al. Post-transcriptional repression of circadian component CLOCK regulates cancer-stemness in murine breast cancer cells. Elife. 2021;10:e66155. 33890571 10.7554/eLife.66155 PMC8102063

[29] Hoffman AE, Yi CH, Zheng T, Stevens RG, Leaderer D, Zhang Y, et al. CLOCK in breast tumorigenesis: genetic, epigenetic, and transcriptional profiling analyses. Cancer Res. 2010;70:1459–68. 20124474 10.1158/0008-5472.CAN-09-3798 PMC3188957

[30] Lesicka M, Jabłońska E, Wieczorek E, Seroczyńska B, Kalinowski L, Skokowski J, et al. A different methylation profile of circadian genes promoter in breast cancer patients according to clinicopathological features. Chronobiol Int. 2019;36:1103–14. 31179760 10.1080/07420528.2019.1617732

[31] Rasmussen NL, Kournoutis A, Lamark T, Johansen T. NBR1: the archetypal selective autophagy receptor. J Cell Biol. 2022;221:e202208092. 36255390 10.1083/jcb.202208092 PMC9582228

[32] Sánchez-Martín P, Sou YS, Kageyama S, Koike M, Waguri S, Komatsu M. NBR1-mediated p62-liquid droplets enhance the Keap1-Nrf2 system. EMBO Rep. 2020;21:e48902. 31916398 10.15252/embr.201948902 PMC7054683

[33] Gallagher ER, Holzbaur ELF. SQSTM1/P62 promotes lysophagy via formation of liquid-like condensates maintained by HSP27. Autophagy. 2023;19:3029–30. 37194327 10.1080/15548627.2023.2210943 PMC10548893

[34] Kim K, Kim JH, Kim I, Seong S, Kim N. TRIM38 regulates NF-κB activation through TAB2 degradation in osteoclast and osteoblast differentiation. Bone. 2018;113:17–28. 29753717 10.1016/j.bone.2018.05.009

[35] Hu S, Li Y, Wang B, Peng K. TRIM38 protects chondrocytes from IL-1β-induced apoptosis and degeneration via negatively modulating nuclear factor (NF)-κB signaling. Int Immunopharmacol. 2021;99:108048. 34426118 10.1016/j.intimp.2021.108048

[36] Zhang K, Lin G, Nie Z, Jin S, Bing X, Li Z, et al. TRIM38 suppresses migration, invasion, metastasis, and proliferation in non-small cell lung cancer (NSCLC) via regulating the AMPK/NF-κB/NLRP3 pathway. Mol Cell Biochem. 2024;479:2069–79. 37566200 10.1007/s11010-023-04823-y

[37] Vickridge E, Faraco CCF, Lo F, Rahimian H, Liu ZY, Tehrani PS, et al. The function of BCL11B in base excision repair contributes to its dual role as an oncogene and a haplo-insufficient tumor suppressor gene. Nucleic Acids Res. 2024;52:223–42. 37956270 10.1093/nar/gkad1037 PMC10783527

[38] Kanduri M, Subhash S, Putino R, Mahale S, Kanduri C. IER3: exploring its dual function as an oncogene and tumor suppressor. Cancer Gene Ther. 2025;32:450–63. 40090972 10.1038/s41417-025-00891-y PMC11976266

[39] Stokes K, Nunes M, Trombley C, Flôres D, Wu G, Taleb Z, et al. The Circadian Clock Gene, Bmal1, Regulates Intestinal Stem Cell Signaling and Represses Tumor Initiation. Cell Mol Gastroenterol Hepatol. 2021;12:1847–72.e0. 34534703 10.1016/j.jcmgh.2021.08.001 PMC8591196

[40] Huang Z, Liu Z, Chen L, Liu Y, Yan G, Ni Y, et al. Liquid-liquid phase separation in cell physiology and cancer biology: recent advances and therapeutic implications. Front Oncol. 2025;15:1540427. 40231263 10.3389/fonc.2025.1540427 PMC11994588

[41] Noguchi T, Sekiguchi Y, Shimada T, Suzuki W, Yokosawa T, Itoh T, et al. LLPS of SQSTM1/p62 and NBR1 as outcomes of lysosomal stress response limits cancer cell metastasis. Proc Natl Acad Sci USA. 2023;120:e2311282120. 37847732 10.1073/pnas.2311282120 PMC10614216

[42] Tozawa T, Matsunaga K, Izumi T, Shigehisa N, Uekita T, Taoka M, et al. Ubiquitination-coupled liquid phase separation regulates the accumulation of the TRIM family of ubiquitin ligases into cytoplasmic bodies. PLoS One. 2022;17:e0272700. 35930602 10.1371/journal.pone.0272700 PMC9355226

[43] Chen Q, Yang P. Coiled-coil-domain-mediated TRIM E3 protein condensation modulates enzymatic activity and functional specificity. Cell Rep. 2025;44:115766. 40450692 10.1016/j.celrep.2025.115766

[44] Yang C, Wang Z, Kang Y, Yi Q, Wang T, Bai Y, et al. Stress granule homeostasis is modulated by TRIM21-mediated ubiquitination of G3BP1 and autophagy-dependent elimination of stress granules. Autophagy. 2023;19:1934–51. 36692217 10.1080/15548627.2022.2164427 PMC10283440

