# Supplementary material for: Circadian gene CLOCK-mediated LLPS of NBR1 and p62/SQSTM1 inhibits proliferation and migration of nasopharyngeal carcinoma
Source: Cell Death Dis. 2026 Jun 10;17(1):734. doi: 10.1038/s41419-026-08957-x (PMC13478613; doi:10.1038/s41419-026-08957-x)

Figure. 2A

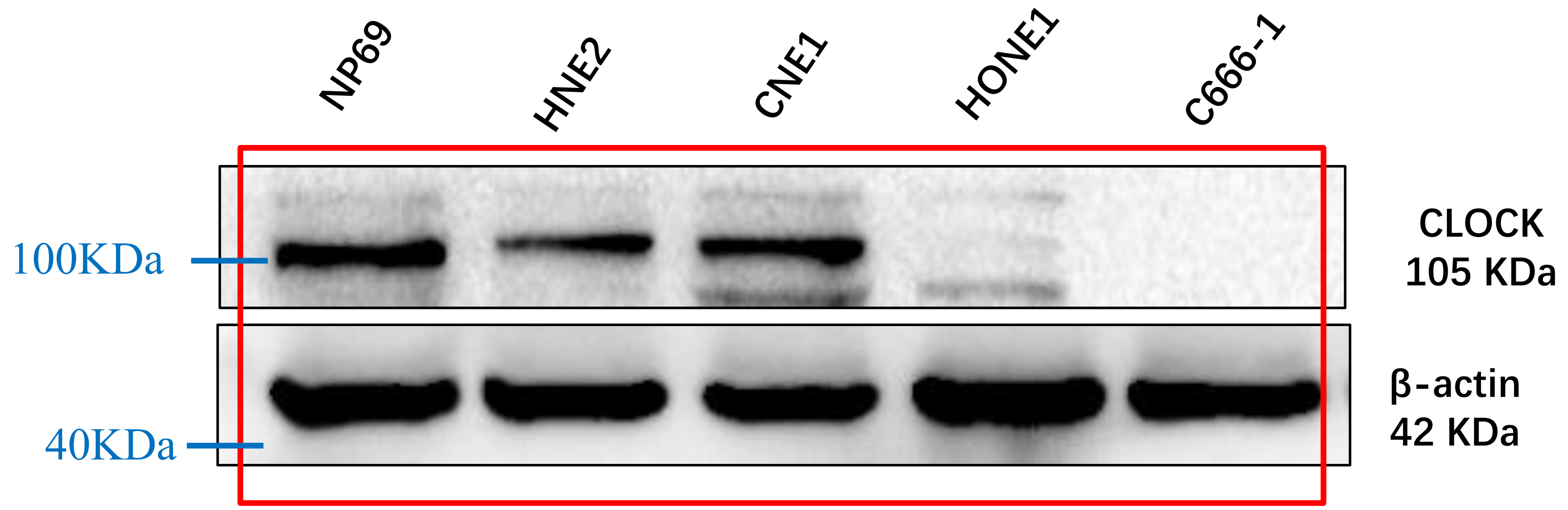

NP69
HNE2
CNE1
HONE1
C666-1
100KDa
40KDa
CLOCK
105 KDa
β-actin
42 KDa

Figure. 2B

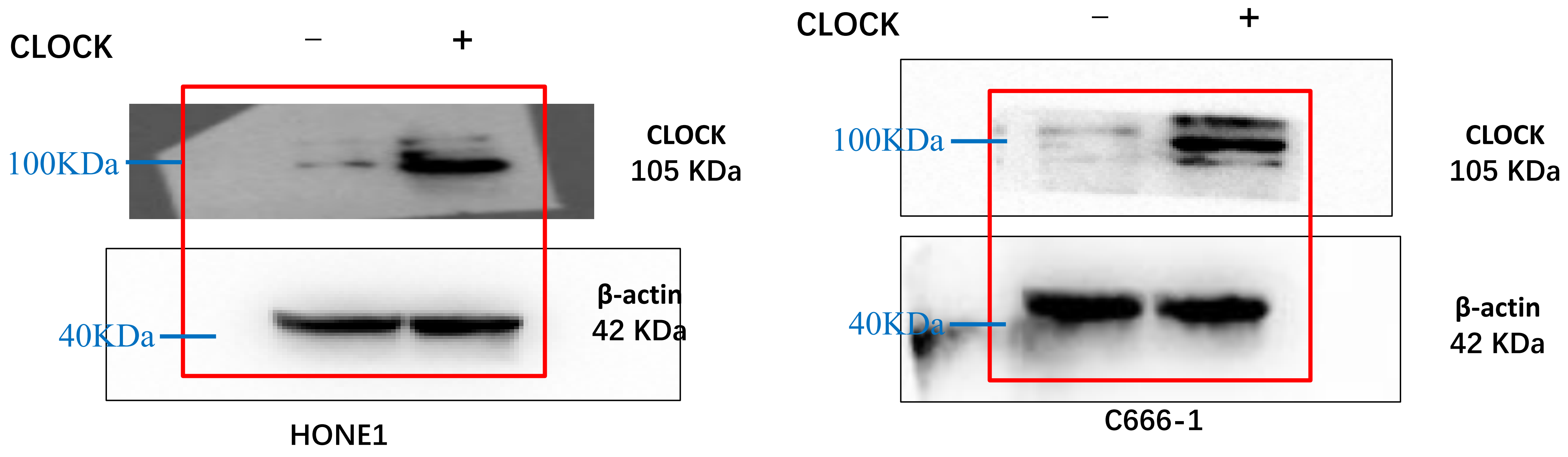

CLOCK
–
+
100KDa
CLOCK
105 KDa
40KDa
β-actin
42 KDa
HONE1
CLOCK
–
+
100KDa
CLOCK
105 KDa
40KDa
β-actin
42 KDa
C666-1

Figure. 3A

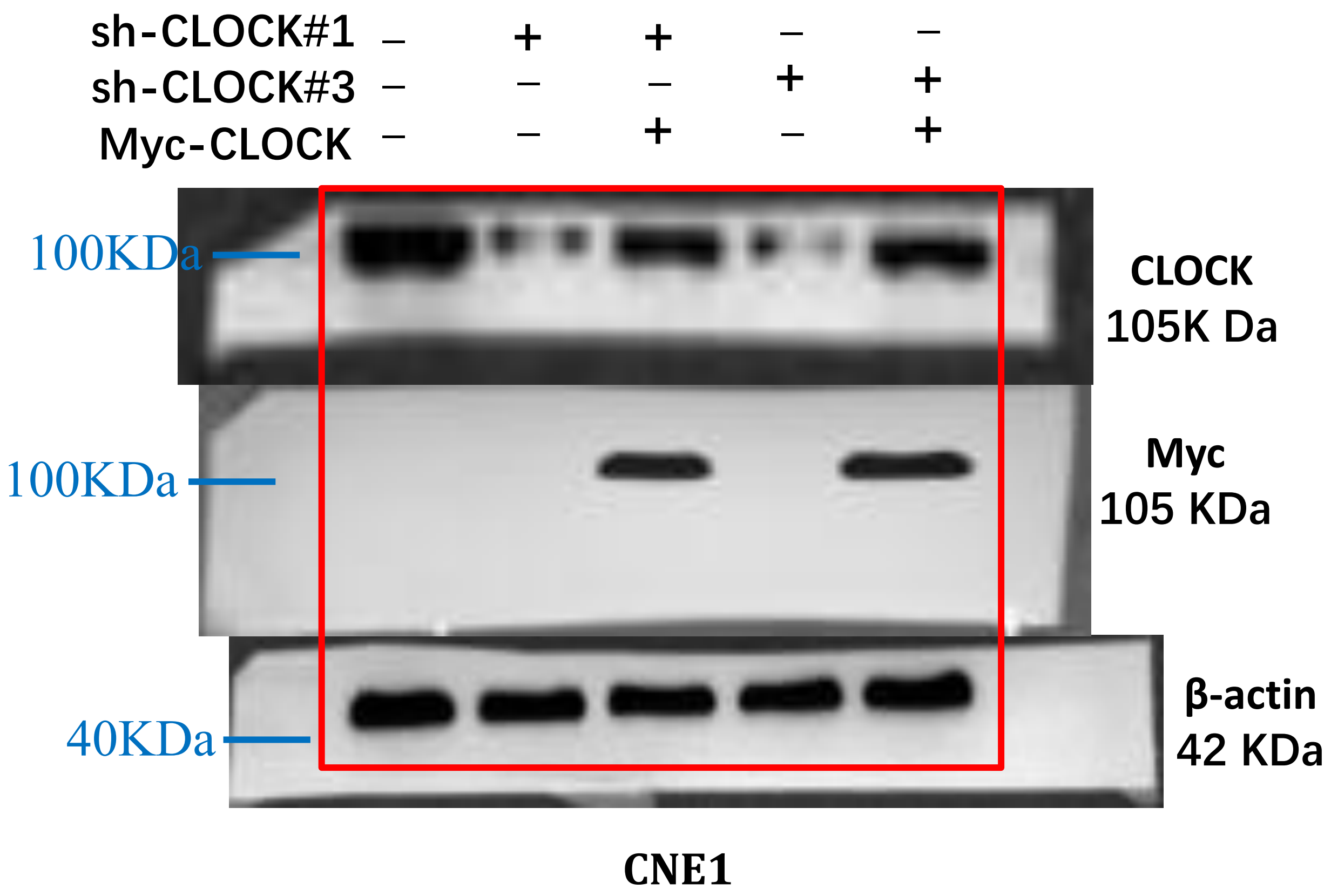

sh-CLOCK#1 – + + – –
sh-CLOCK#3 – – – + +
Myc-CLOCK – – + – +
100KDa
CLOCK
105K Da
100KDa
Myc
105 KDa
40KDa
β-actin
42 KDa
CNE1

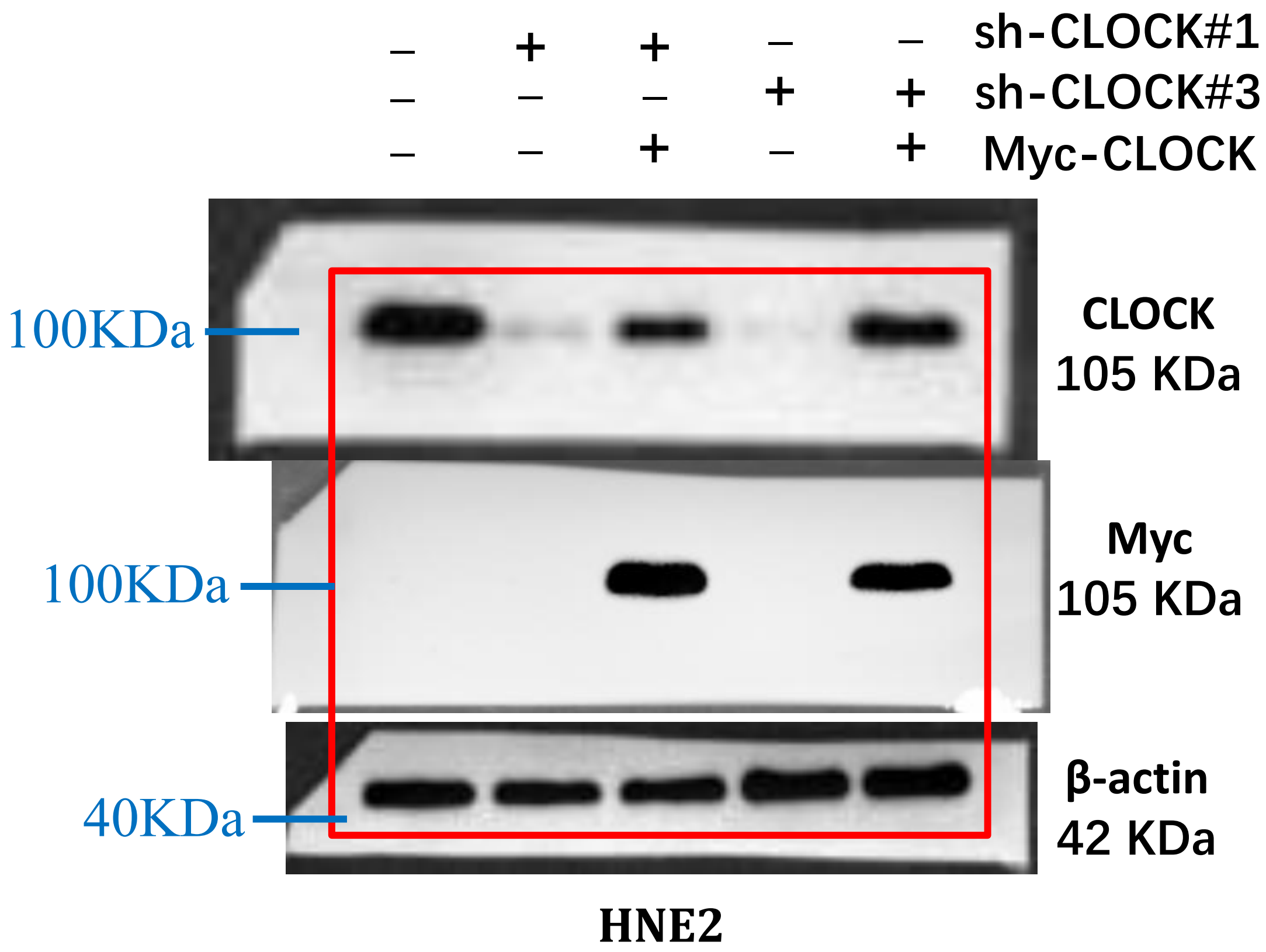

– + + – – sh-CLOCK#1
– – – + + sh-CLOCK#3
– – + – + Myc-CLOCK
100KDa
CLOCK
105 KDa
100KDa
Myc
105 KDa
40KDa
β-actin
42 KDa
HNE2

Figure. 4C

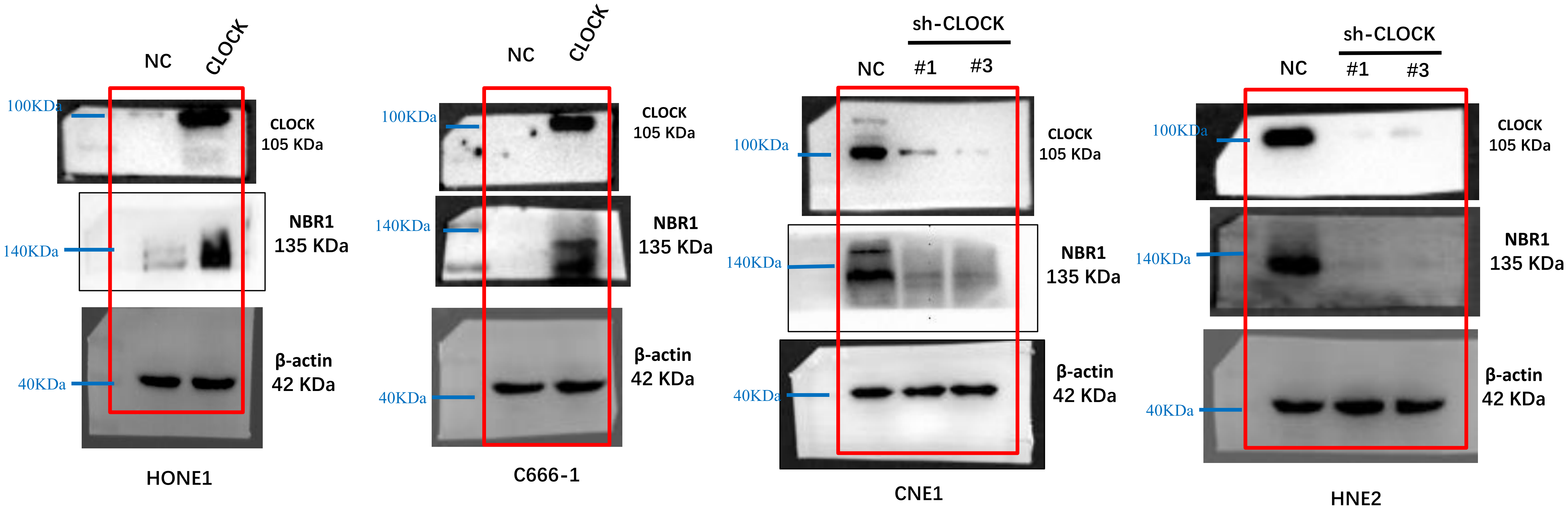
NC
CLOCK
100KDa
CLOCK
105 KDa
140KDa
NBR1
135 KDa
40KDa
β-actin
42 KDa
HONE1
NC
CLOCK
100KDa
CLOCK
105 KDa
140KDa
NBR1
135 KDa
40KDa
β-actin
42 KDa
C666-1
sh-CLOCK
NC
#1
#3
100KDa
CLOCK
105 KDa
140KDa
NBR1
135 KDa
40KDa
β-actin
42 KDa
CNE1
sh-CLOCK
NC
#1
#3
100KDa
CLOCK
105 KDa
140KDa
NBR1
135 KDa
40KDa
β-actin
42 KDa
HNE2

Figure. 5B

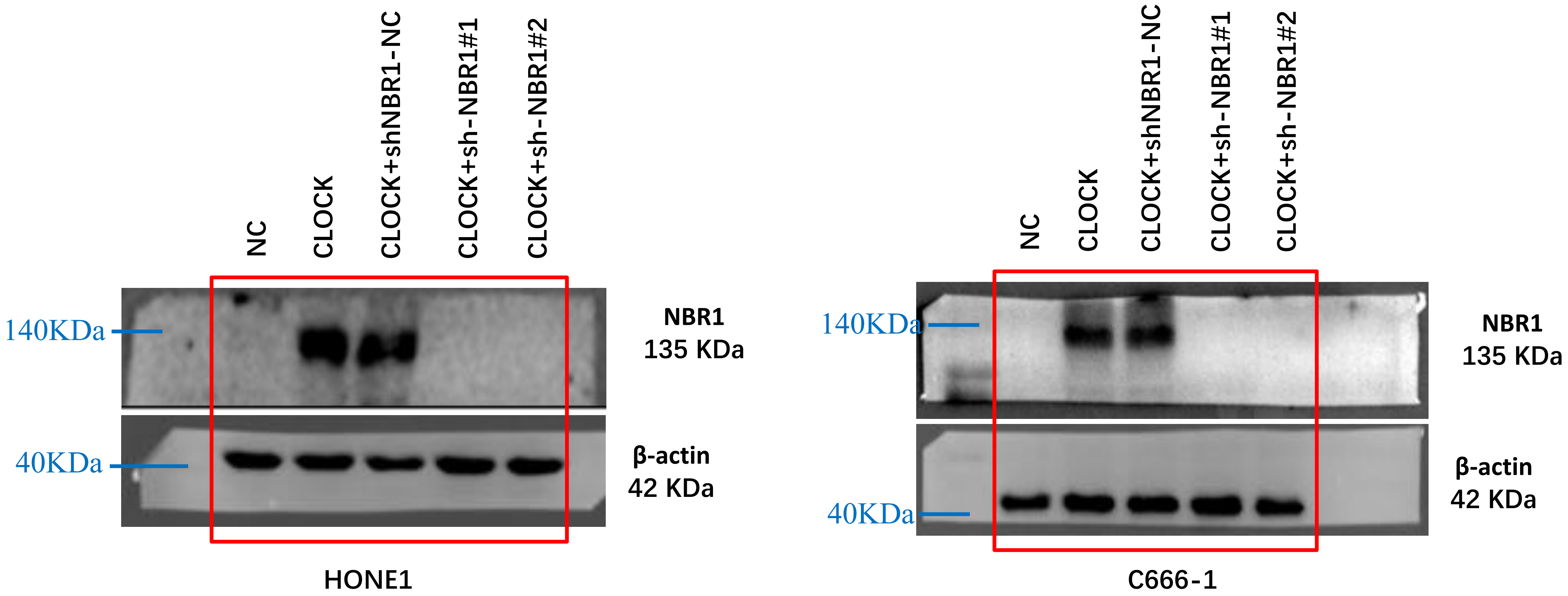
NC
CLOCK
CLOCK+shNBR1-NC
CLOCK+sh-NBR1#1
CLOCK+sh-NBR1#2
140KDa
40KDa
NBR1
135 KDa
β-actin
42 KDa
HONE1
NC
CLOCK
CLOCK+shNBR1-NC
CLOCK+sh-NBR1#1
CLOCK+sh-NBR1#2
140KDa
40KDa
NBR1
135 KDa
β-actin
42 KDa
C666-1

Figure. 6A

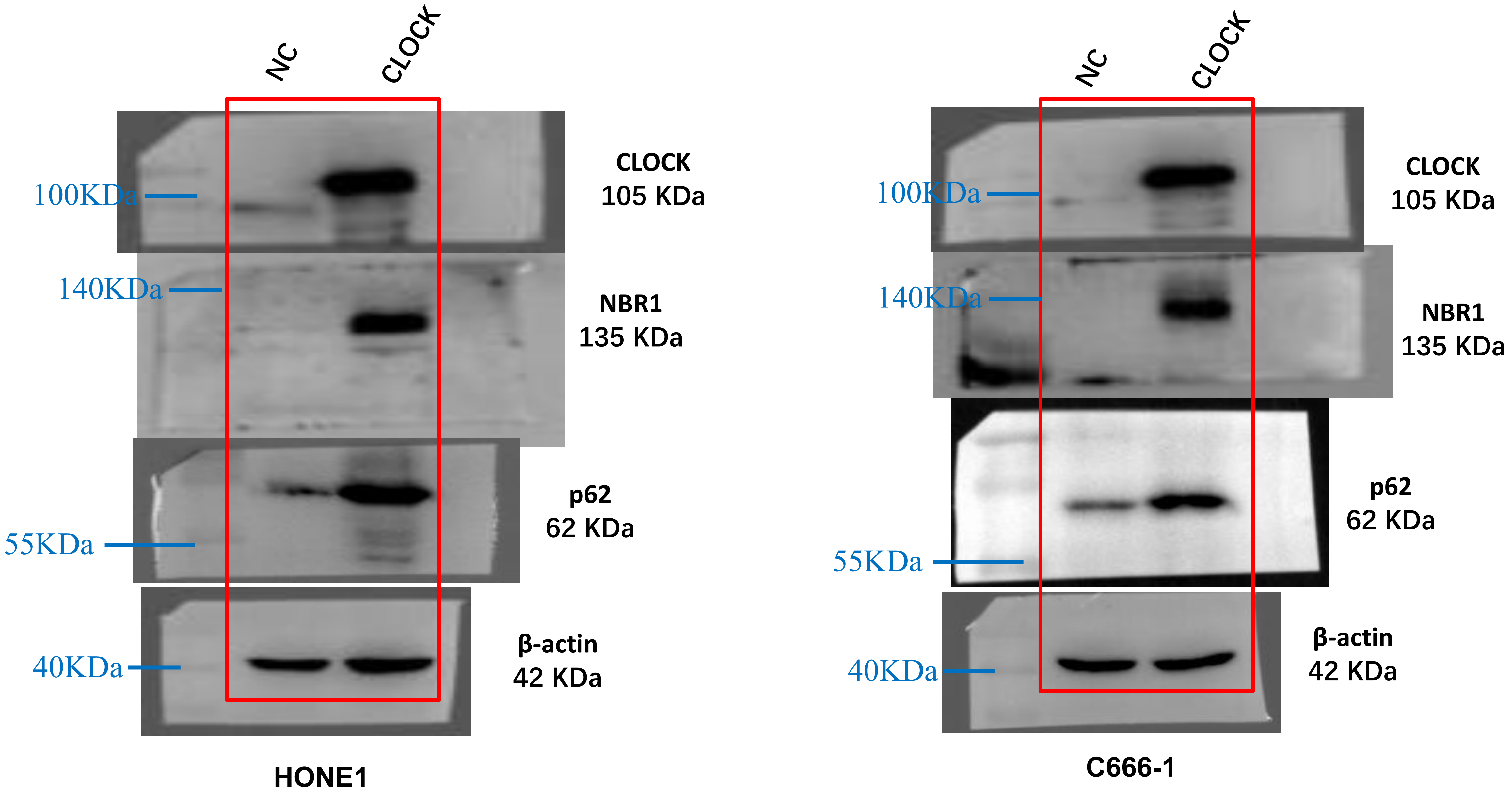
NC
CLOCK
100KDa
140KDa
55KDa
40KDa
CLOCK
105 KDa
NBR1
135 KDa
p62
62 KDa
β-actin
42 KDa
HONE1
NC
CLOCK
100KDa
140KDa
55KDa
40KDa
CLOCK
105 KDa
NBR1
135 KDa
p62
62 KDa
β-actin
42 KDa
C666-1

Figure. 6B

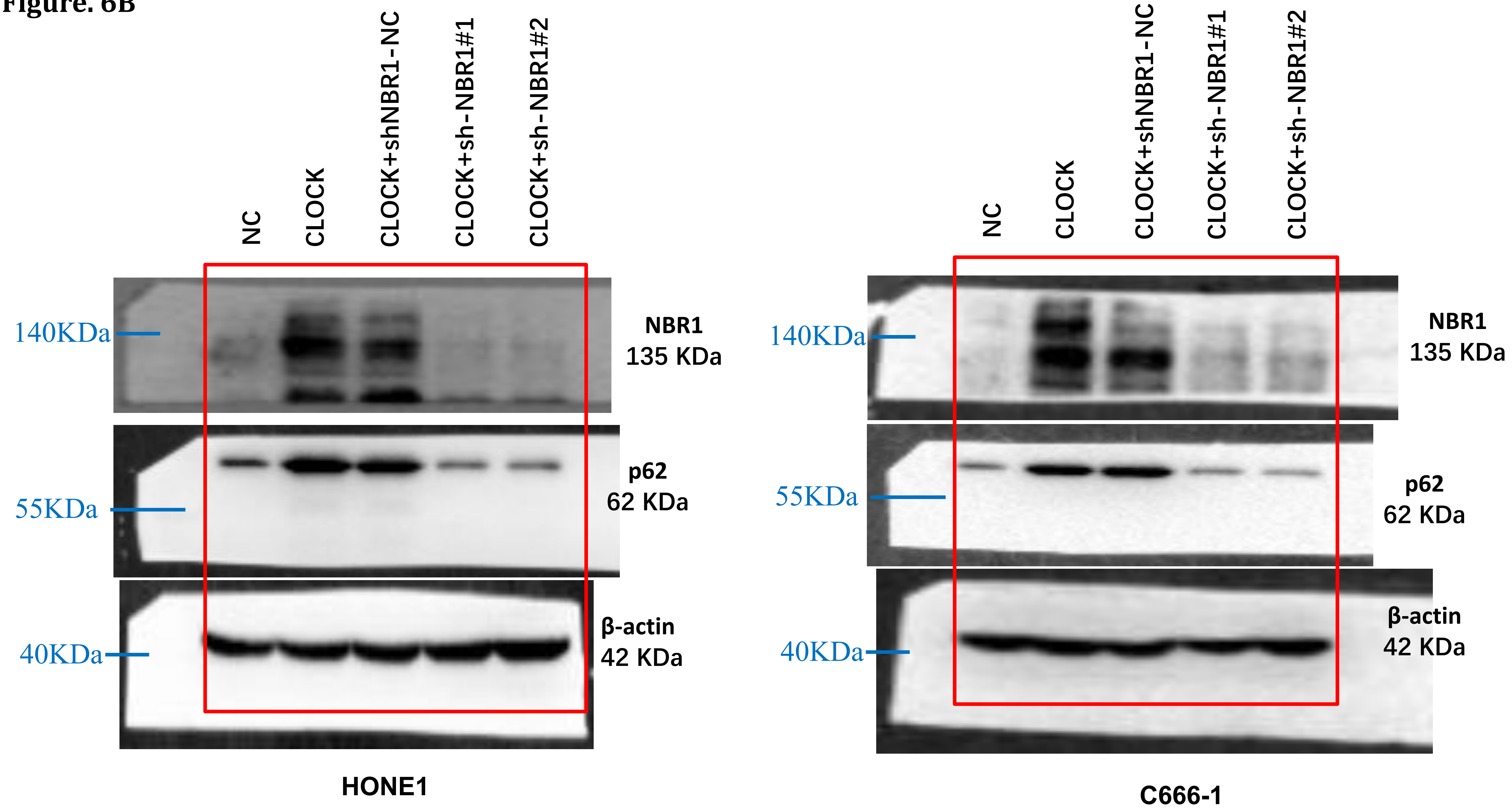
NC
CLOCK
CLOCK+shNBR1-NC
CLOCK+sh-NBR1#1
CLOCK+sh-NBR1#2
140KDa
55KDa
40KDa
NBR1
135 KDa
p62
62 KDa
β-actin
42 KDa
HONE1
NC
CLOCK
CLOCK+shNBR1-NC
CLOCK+sh-NBR1#1
CLOCK+sh-NBR1#2
140KDa
55KDa
40KDa
NBR1
135 KDa
p62
62 KDa
β-actin
42 KDa
C666-1

Figure. 6C

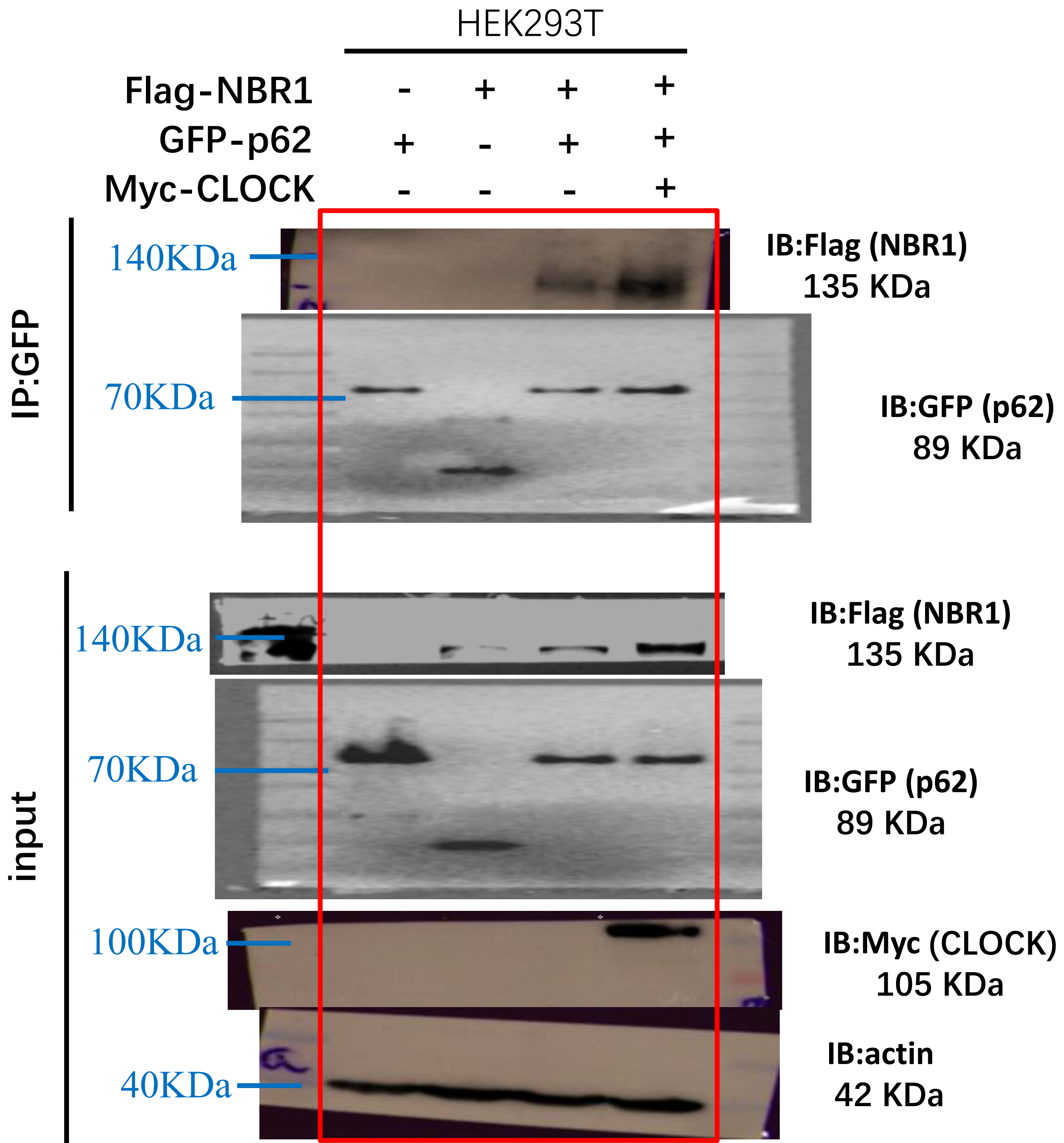
HEK293T
Flag-NBR1 - + + +
GFP-p62 + - + +
Myc-CLOCK - - - +
IP:GFP
140KDa
IB:Flag (NBR1)
135 KDa
70KDa
IB:GFP (p62)
89 KDa
input
140KDa
IB:Flag (NBR1)
135 KDa
70KDa
IB:GFP (p62)
89 KDa
100KDa
IB:Myc (CLOCK)
105 KDa
40KDa
IB:actin
42 KDa

Figure. 6D

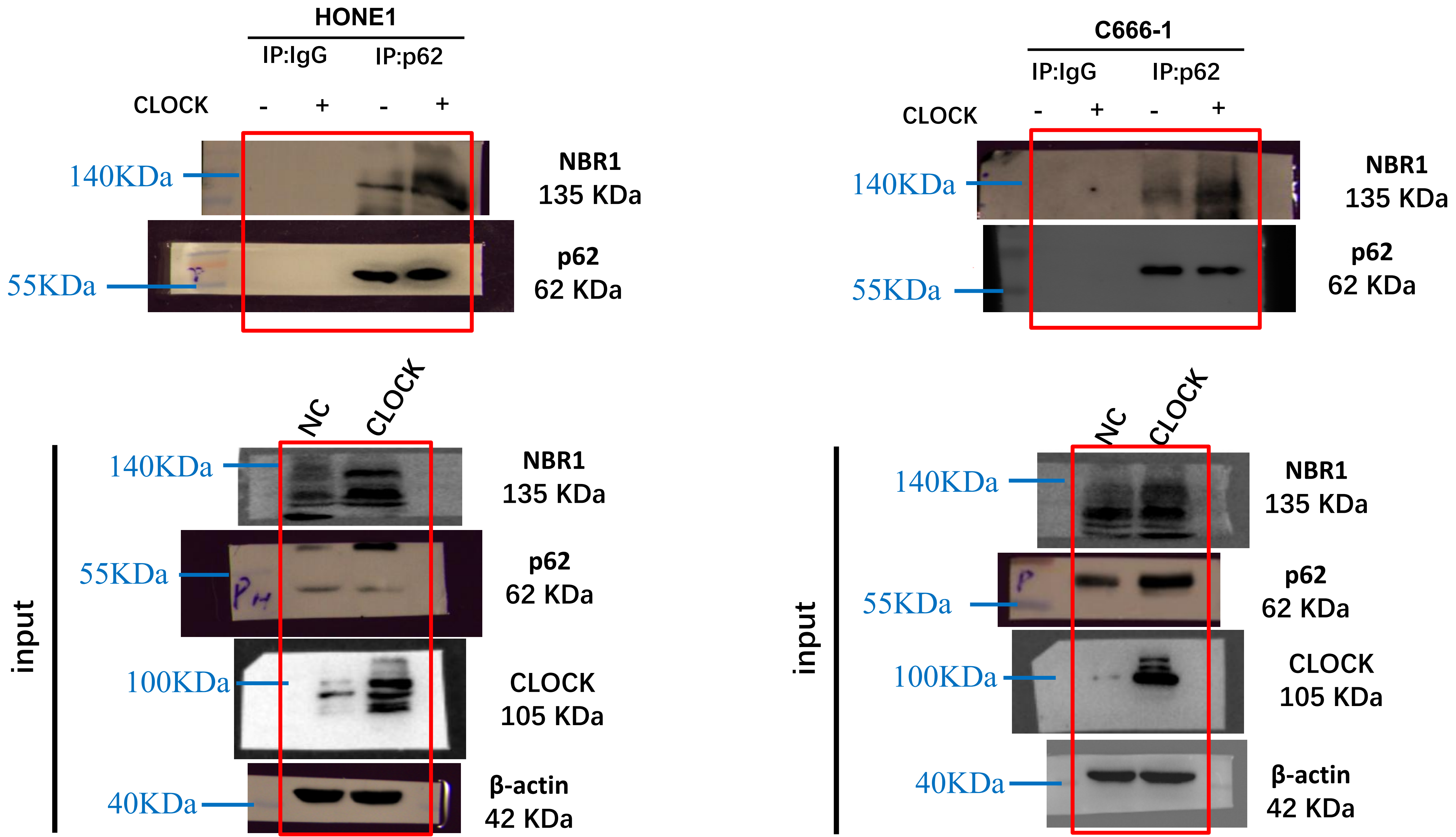
HONE1
IP:IgG
IP:p62
CLOCK
- + - +
140KDa
55KDa
NBR1
135 KDa
p62
62 KDa
NC
CLOCK
input
140KDa
55KDa
100KDa
40KDa
NBR1
135 KDa
p62
62 KDa
CLOCK
105 KDa
β-actin
42 KDa
C666-1
IP:IgG
IP:p62
CLOCK
- + - +
140KDa
55KDa
NBR1
135 KDa
p62
62 KDa
NC
CLOCK
input
140KDa
55KDa
100KDa
40KDa
NBR1
135 KDa
p62
62 KDa
CLOCK
105 KDa
β-actin
42 KDa

Figure. 6G

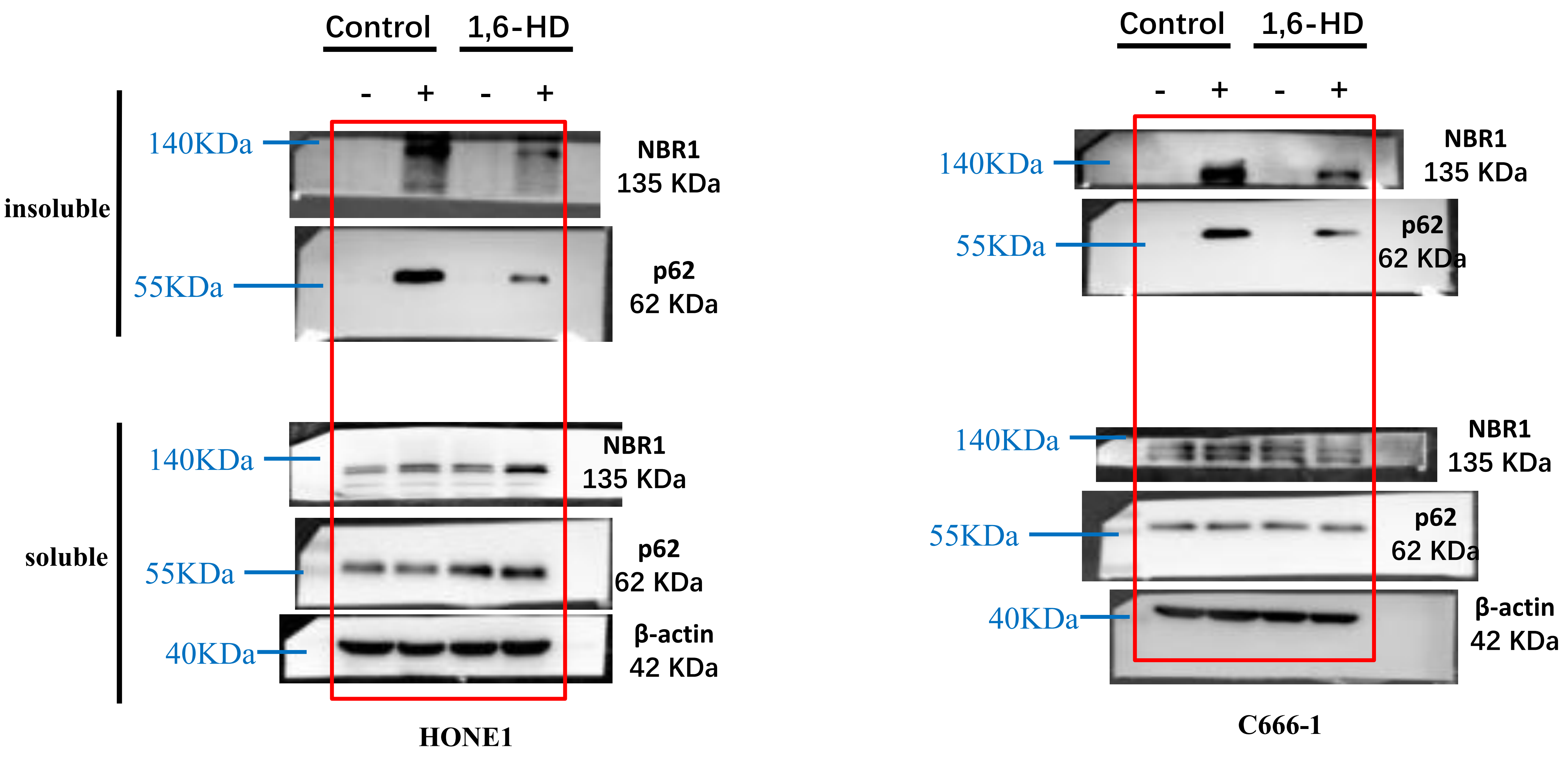

Control
1,6-HD
- + - +
insoluble
140KDa
NBR1
135 KDa
55KDa
p62
62 KDa
soluble
140KDa
NBR1
135 KDa
55KDa
p62
62 KDa
40KDa
β-actin
42 KDa
HONE1
Control
1,6-HD
- + - +
140KDa
NBR1
135 KDa
55KDa
p62
62 KDa
140KDa
NBR1
135 KDa
55KDa
p62
62 KDa
40KDa
β-actin
42 KDa
C666-1

Figure. 7A

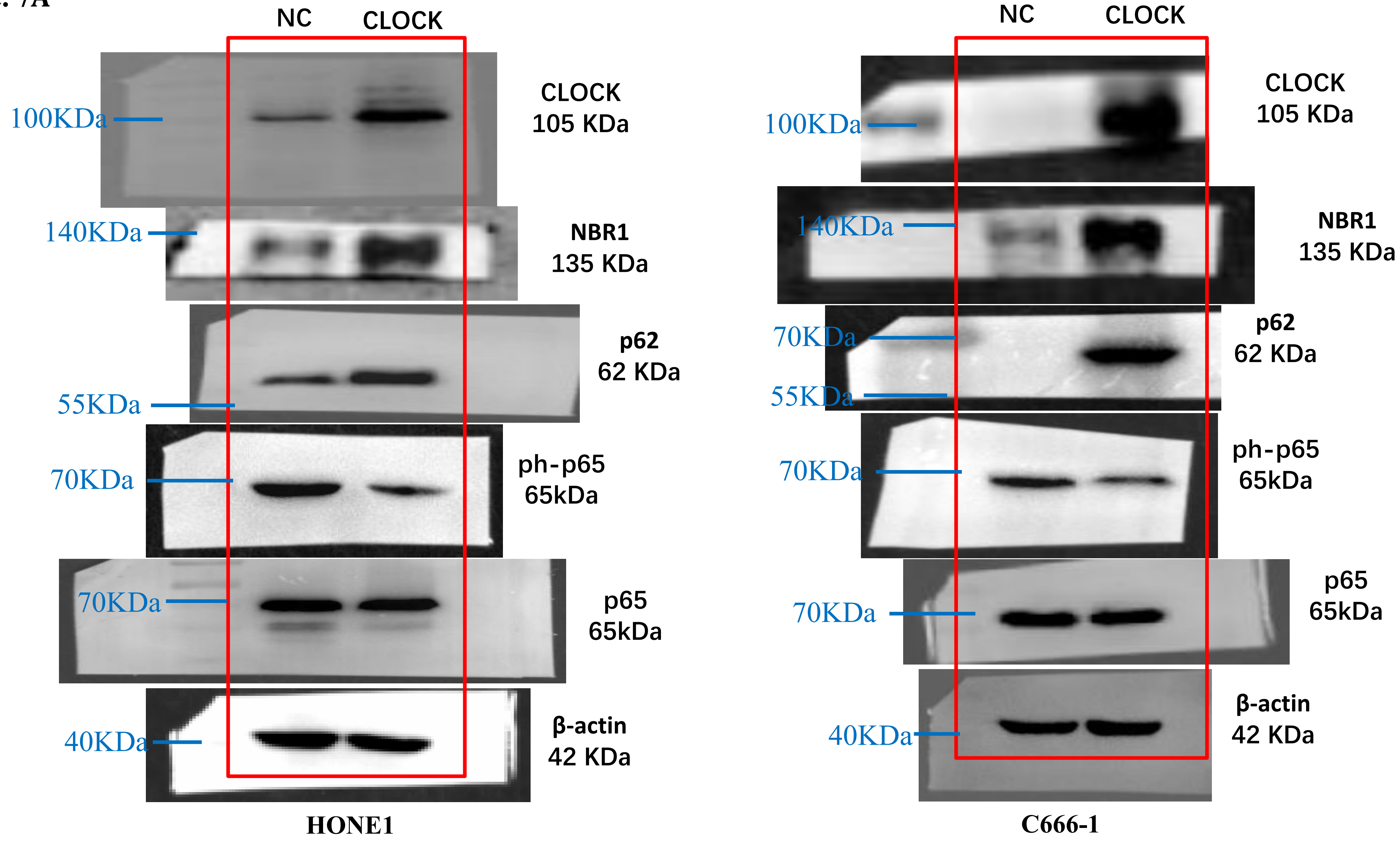

NC
CLOCK
100KDa
CLOCK
105 KDa
140KDa
NBR1
135 KDa
p62
62 KDa
55KDa
70KDa
ph-p65
65kDa
70KDa
p65
65kDa
40KDa
β-actin
42 KDa
HONE1
NC
CLOCK
100KDa
CLOCK
105 KDa
140KDa
NBR1
135 KDa
70KDa
p62
62 KDa
55KDa
70KDa
ph-p65
65kDa
70KDa
p65
65kDa
40KDa
β-actin
42 KDa
C666-1

Figure. 7B

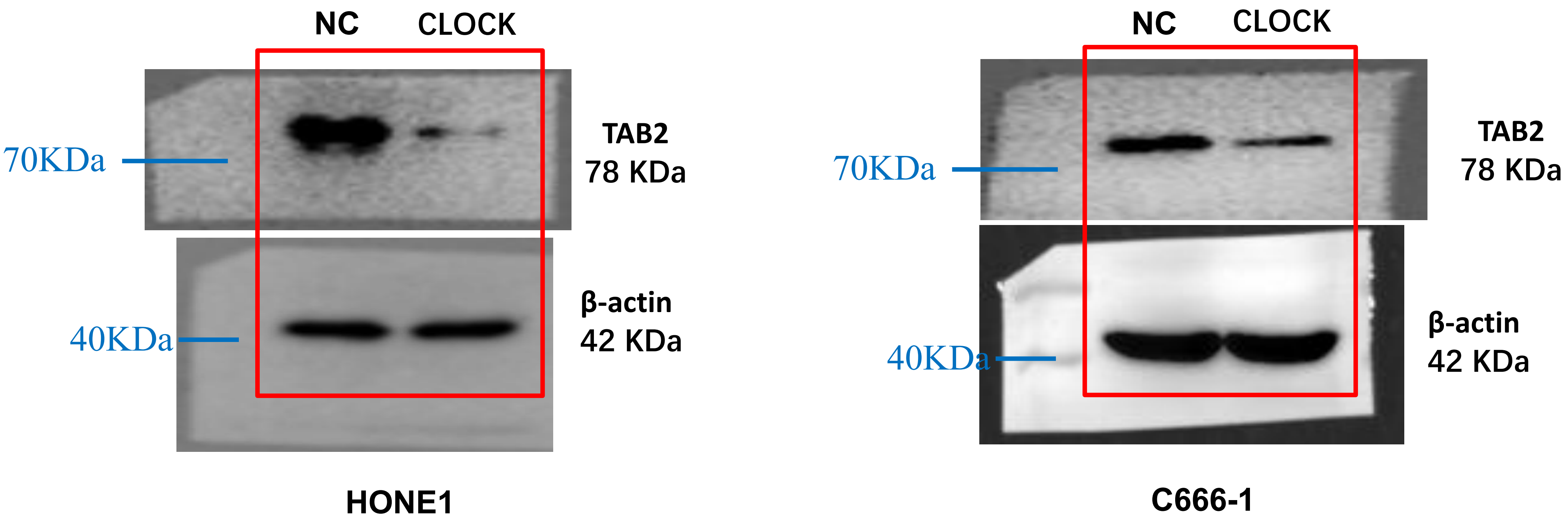
NC
CLOCK
70KDa
TAB2
78 KDa
40KDa
β-actin
42 KDa
HONE1
NC
CLOCK
70KDa
TAB2
78 KDa
40KDa
β-actin
42 KDa
C666-1

Figure. 7C

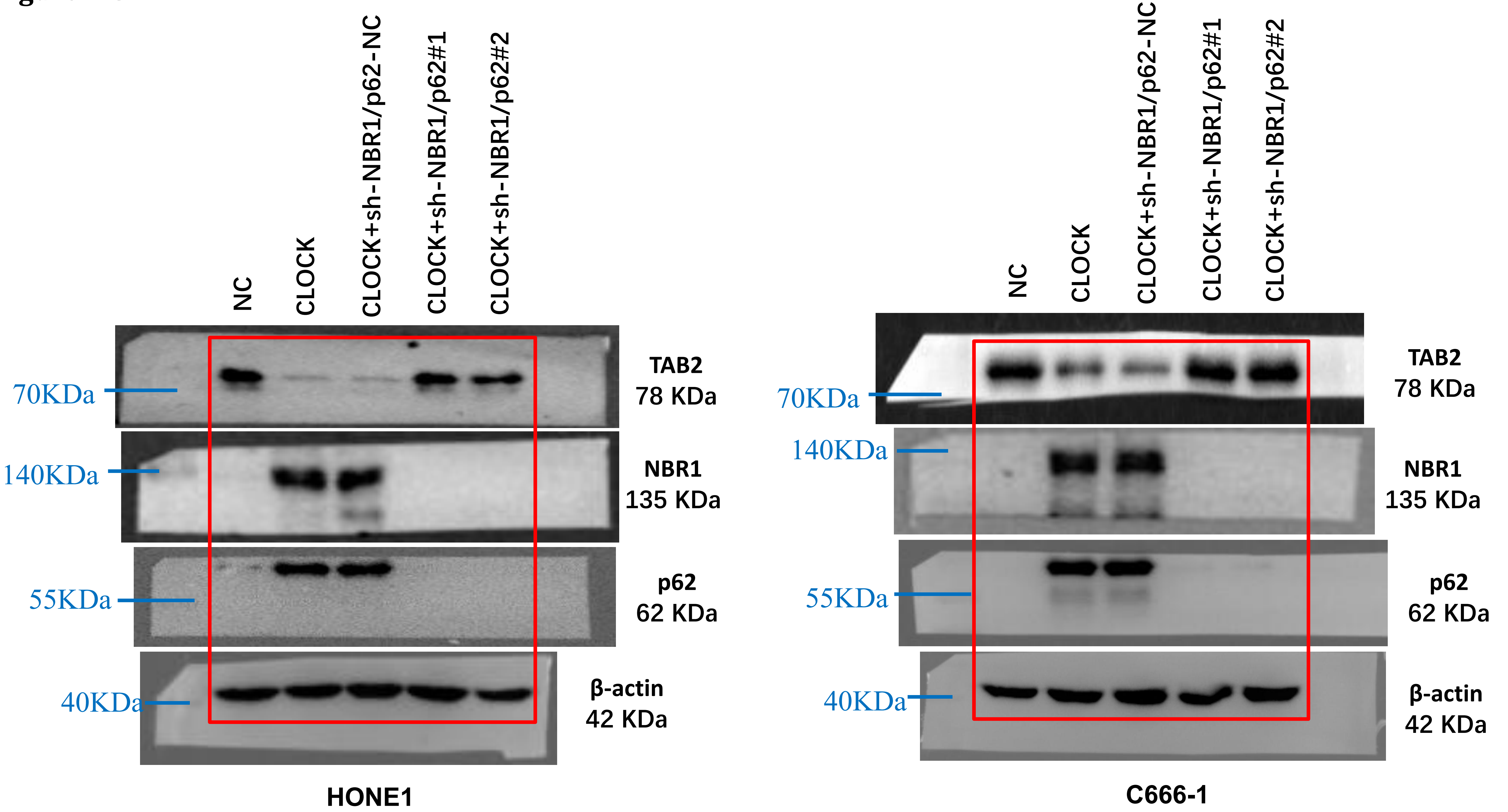

NC
CLOCK
CLOCK+sh-NBR1/p62-NC
CLOCK+sh-NBR1/p62#1
CLOCK+sh-NBR1/p62#2
70KDa
TAB2
78 KDa
140KDa
NBR1
135 KDa
55KDa
p62
62 KDa
40KDa
β-actin
42 KDa
HONE1
C666-1

Figure. 7D

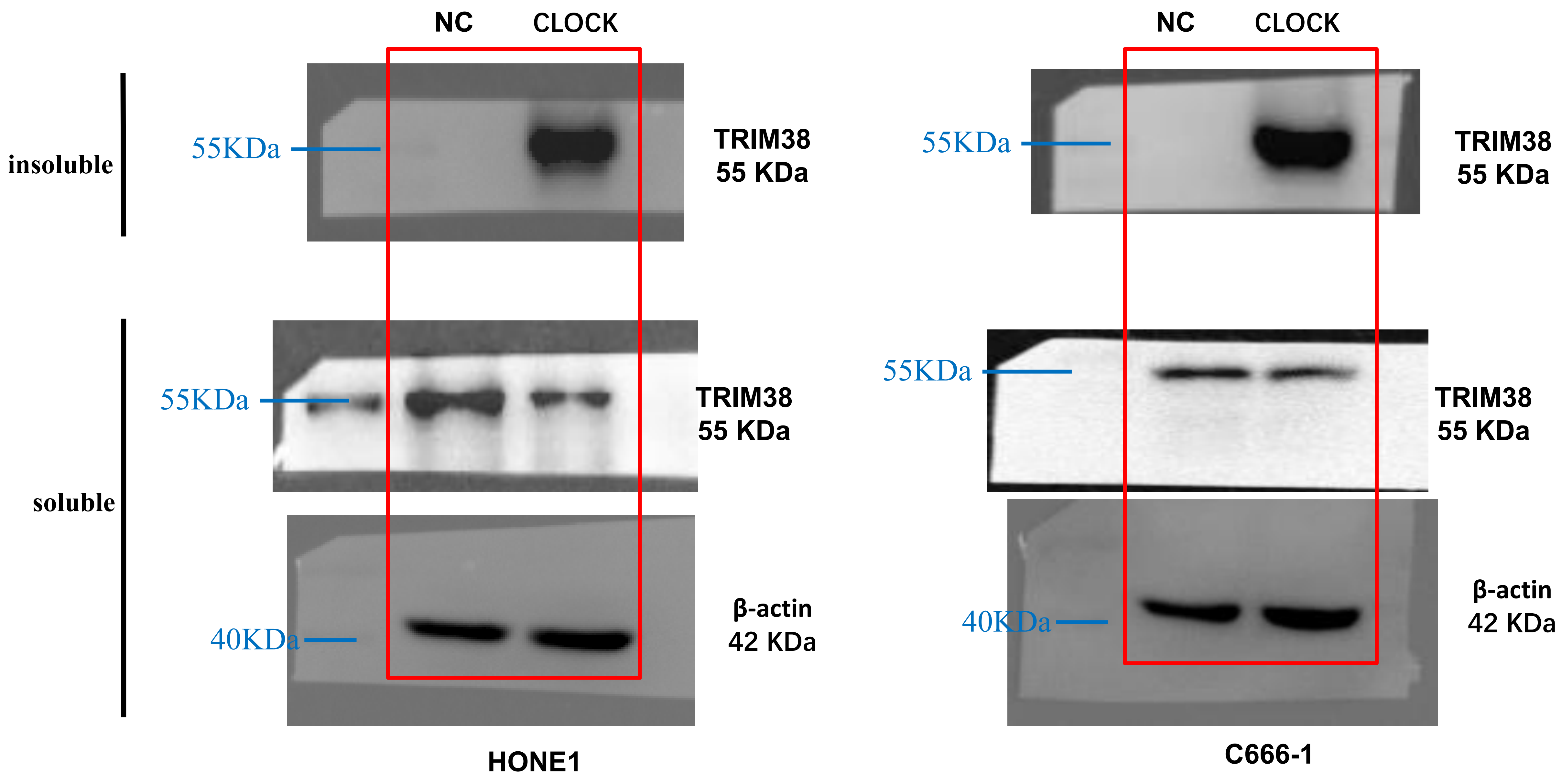
NC
CLOCK
insoluble
55KDa
TRIM38
55 KDa
soluble
55KDa
TRIM38
55 KDa
40KDa
β-actin
42 KDa
HONE1
NC
CLOCK
55KDa
TRIM38
55 KDa
55KDa
TRIM38
55 KDa
40KDa
β-actin
42 KDa
C666-1

Figure. 7F

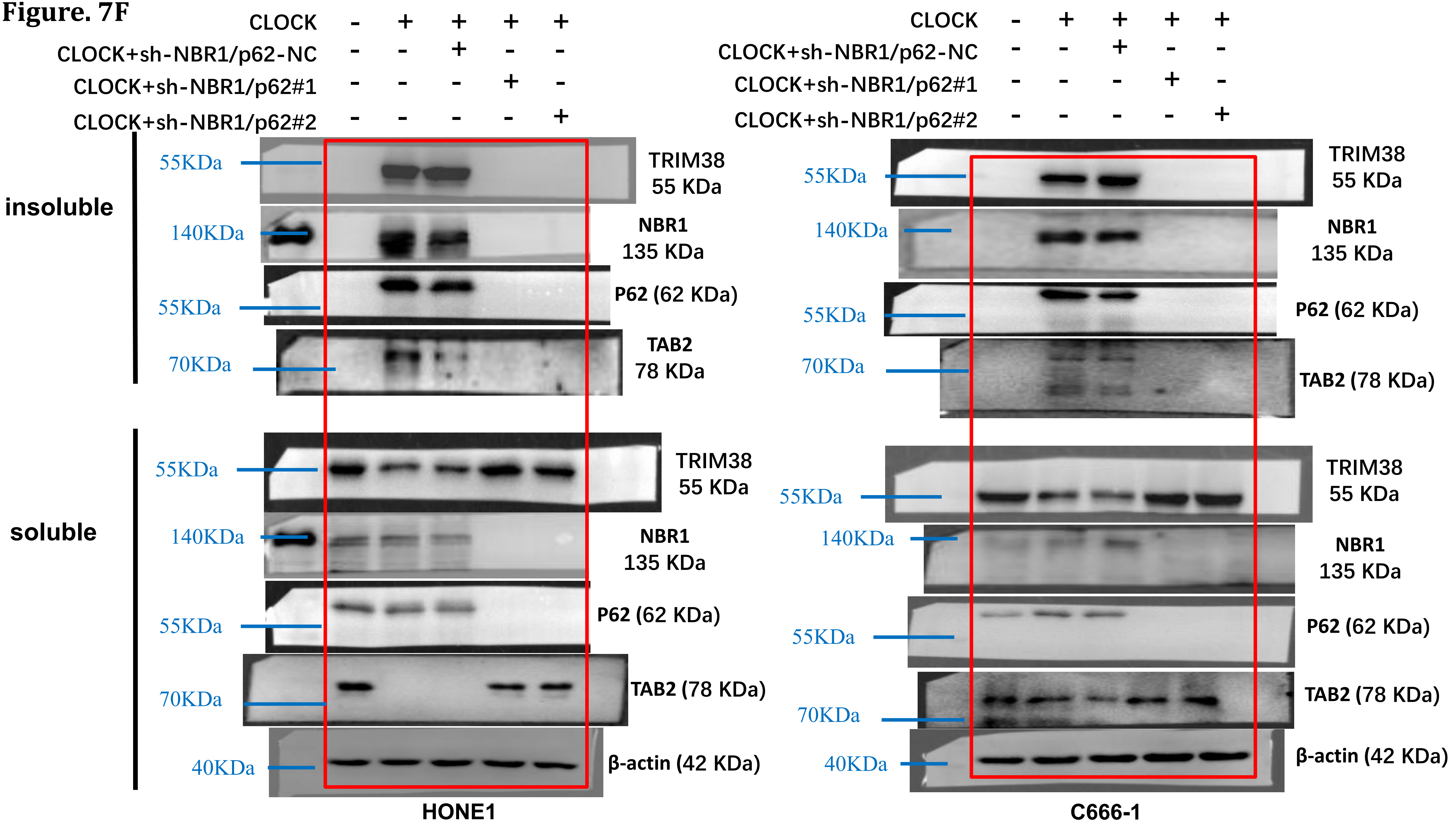

CLOCK - + + + +
CLOCK+sh-NBR1/p62-NC - - + - -
CLOCK+sh-NBR1/p62#1 - - - + -
CLOCK+sh-NBR1/p62#2 - - - - +
insoluble
soluble
55KDa
140KDa
70KDa
40KDa
TRIM38 55 KDa
NBR1 135 KDa
P62 (62 KDa)
TAB2 78 KDa
TAB2 (78 KDa)
β-actin (42 KDa)
HONE1
C666-1

Figure. 7G

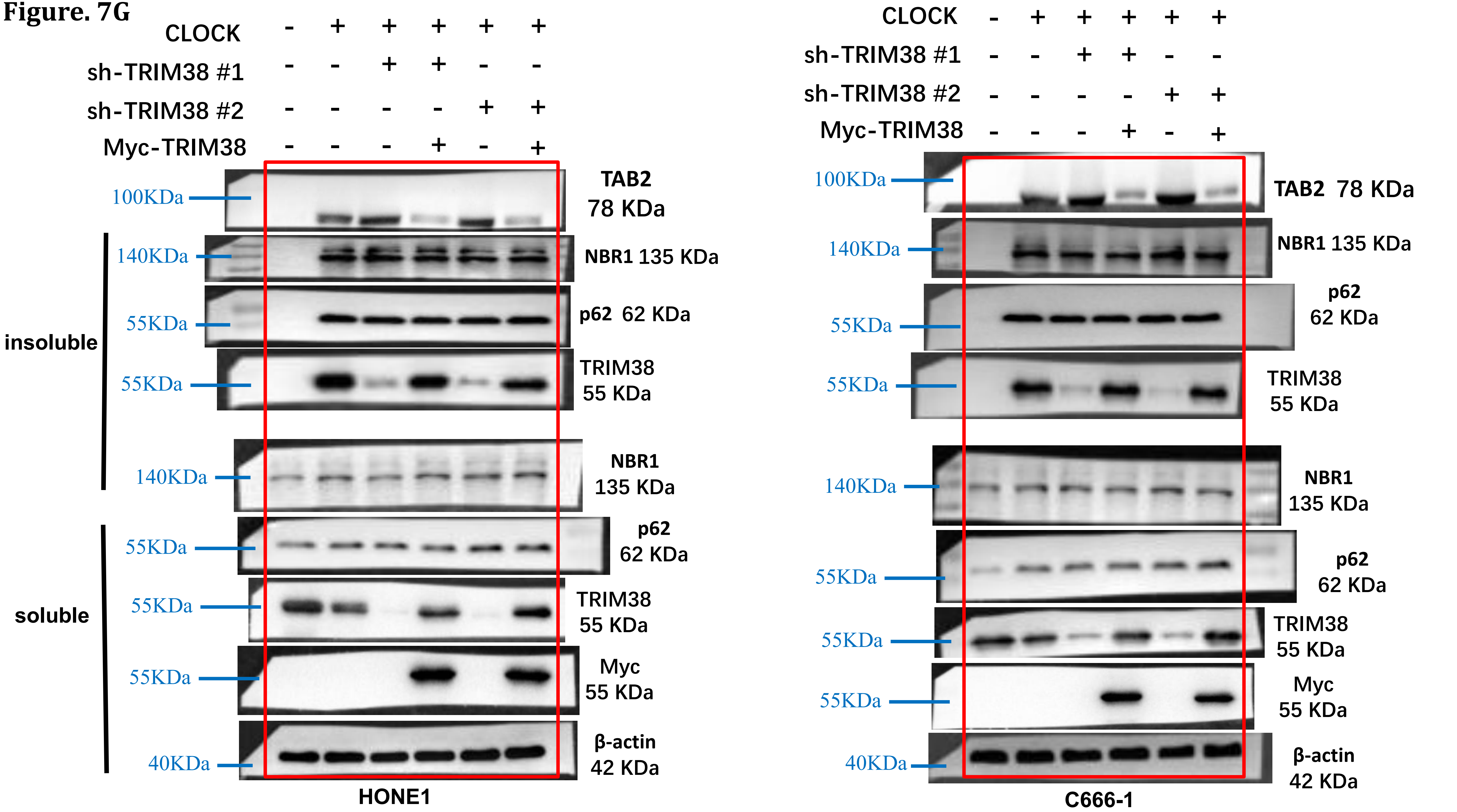

CLOCK - + + + + +
sh-TRIM38 #1 - - + + - -
sh-TRIM38 #2 - - - - + +
Myc-TRIM38 - - - + - +
100KDa
TAB2
78 KDa
140KDa
NBR1 135 KDa
55KDa
p62 62 KDa
55KDa
TRIM38
55 KDa
insoluble
140KDa
NBR1
135 KDa
55KDa
p62
62 KDa
55KDa
TRIM38
55 KDa
soluble
55KDa
Myc
55 KDa
40KDa
β-actin
42 KDa
HONE1
CLOCK - + + + + +
sh-TRIM38 #1 - - + + - -
sh-TRIM38 #2 - - - - + +
Myc-TRIM38 - - - + - +
100KDa
TAB2 78 KDa
140KDa
NBR1 135 KDa
55KDa
p62
62 KDa
55KDa
TRIM38
55 KDa
140KDa
NBR1
135 KDa
55KDa
p62
62 KDa
55KDa
TRIM38
55 KDa
55KDa
Myc
55 KDa
40KDa
β-actin
42 KDa
C666-1

Figure. 7H

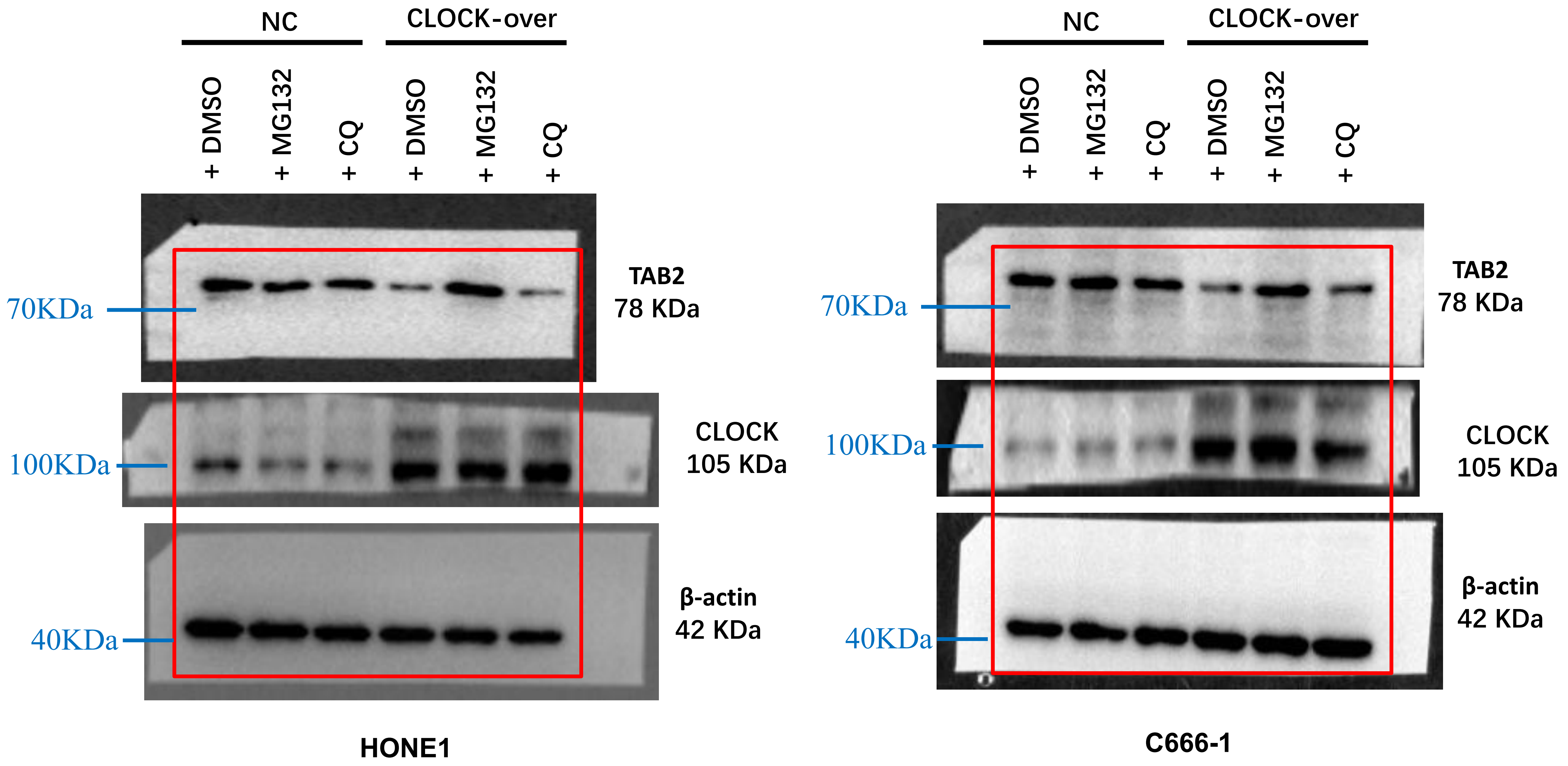

NC
CLOCK-over
+ DMSO
+ MG132
+ CQ
+ DMSO
+ MG132
+ CQ
70KDa
TAB2
78 KDa
100KDa
CLOCK
105 KDa
40KDa
β-actin
42 KDa
HONE1
NC
CLOCK-over
+ DMSO
+ MG132
+ CQ
+ DMSO
+ MG132
+ CQ
70KDa
TAB2
78 KDa
100KDa
CLOCK
105 KDa
40KDa
β-actin
42 KDa
C666-1

Figure. 7I

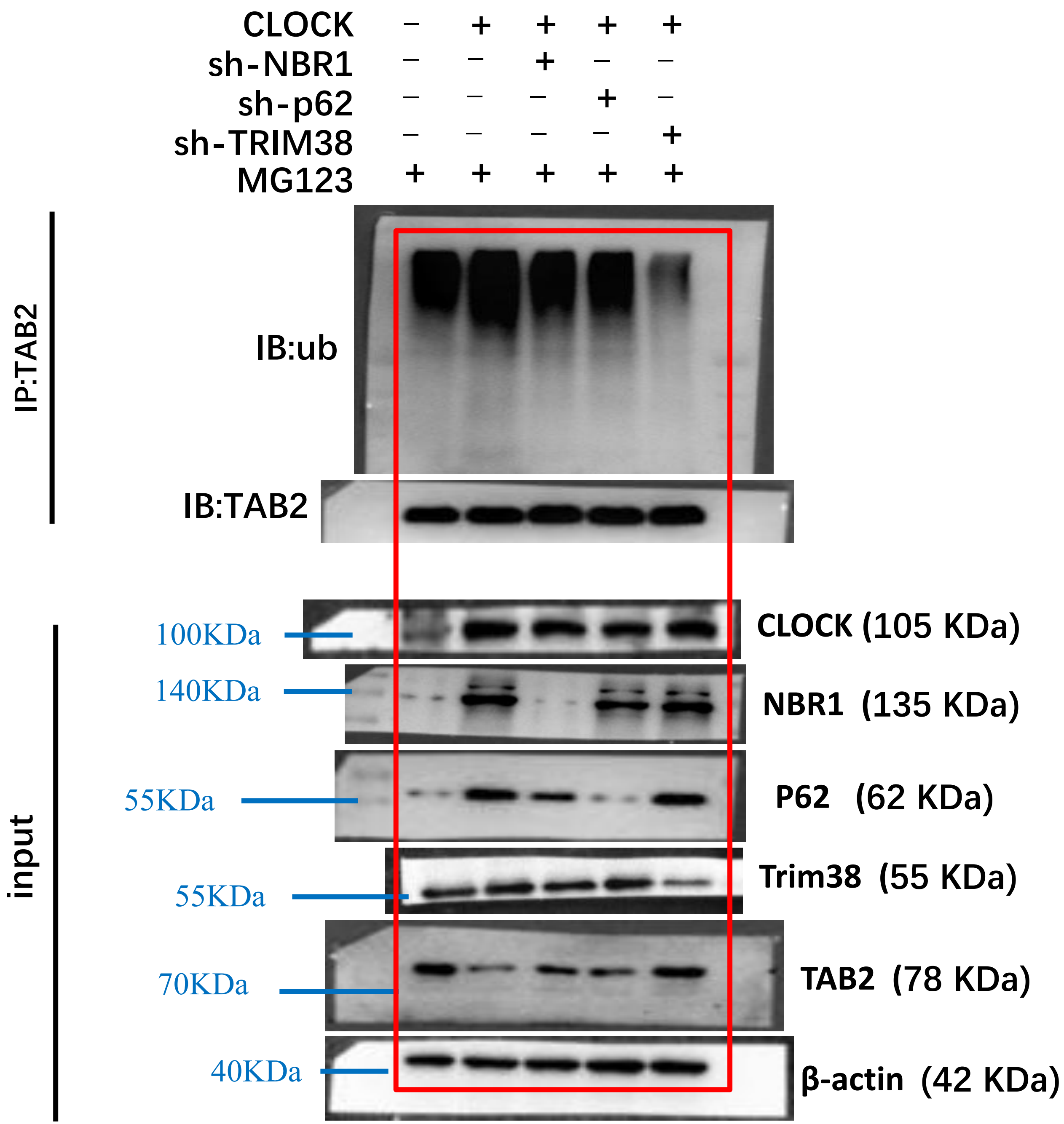

CLOCK − + + + +
sh-NBR1 − − + − −
sh-p62 − − − + −
sh-TRIM38 − − − − +
MG123 + + + + +
IP:TAB2
IB:ub
IB:TAB2
input
100KDa
140KDa
55KDa
55KDa
70KDa
40KDa
CLOCK (105 KDa)
NBR1 (135 KDa)
P62 (62 KDa)
Trim38 (55 KDa)
TAB2 (78 KDa)
β-actin (42 KDa)

Figure. 8D

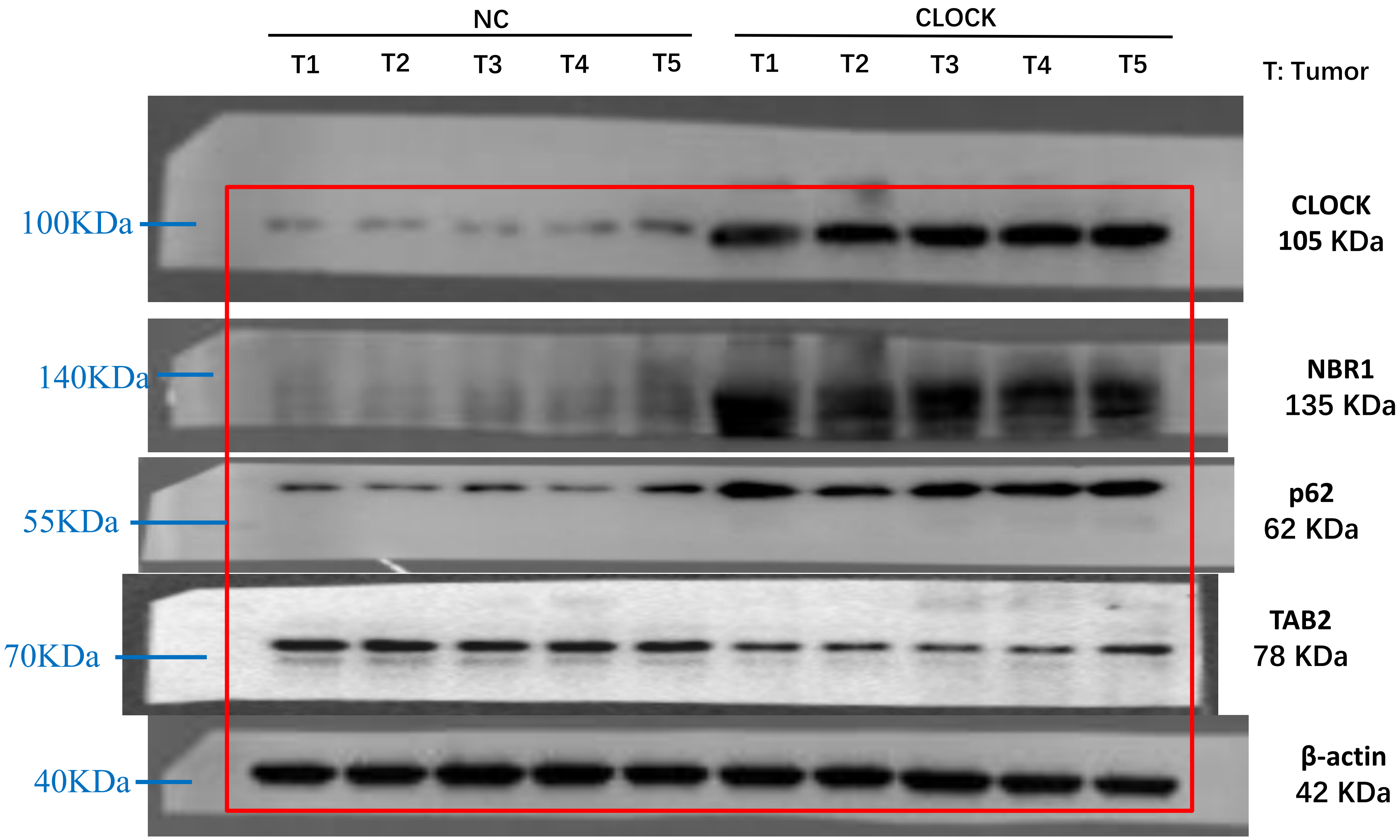
NC
CLOCK
T1
T2
T3
T4
T5
T1
T2
T3
T4
T5
T: Tumor
100KDa
CLOCK
105 KDa
140KDa
NBR1
135 KDa
55KDa
p62
62 KDa
70KDa
TAB2
78 KDa
40KDa
β-actin
42 KDa

Figure. 8E

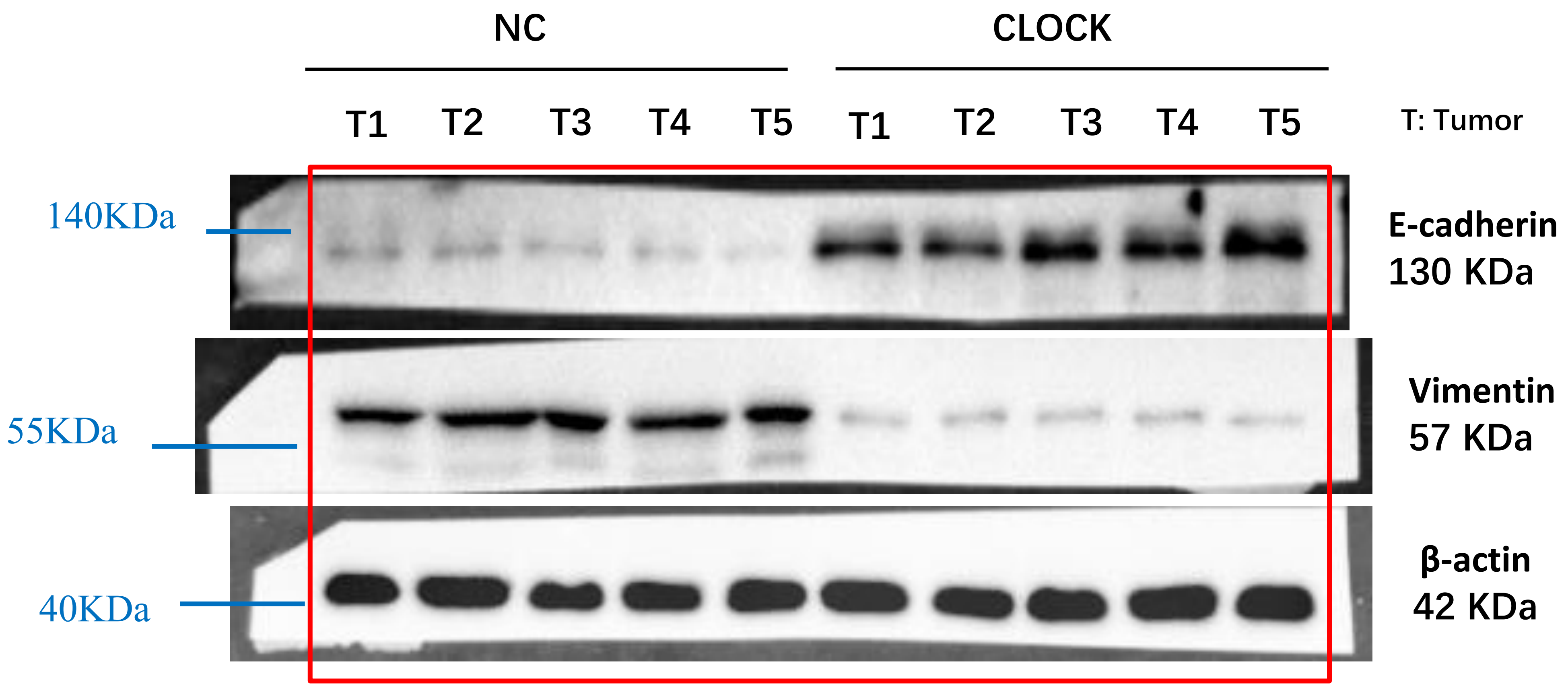
NC
CLOCK
T1
T2
T3
T4
T5
T1
T2
T3
T4
T5
T: Tumor
140KDa
E-cadherin
130 KDa
55KDa
Vimentin
57 KDa
40KDa
β-actin
42 KDa

Supplementary Figure S2A

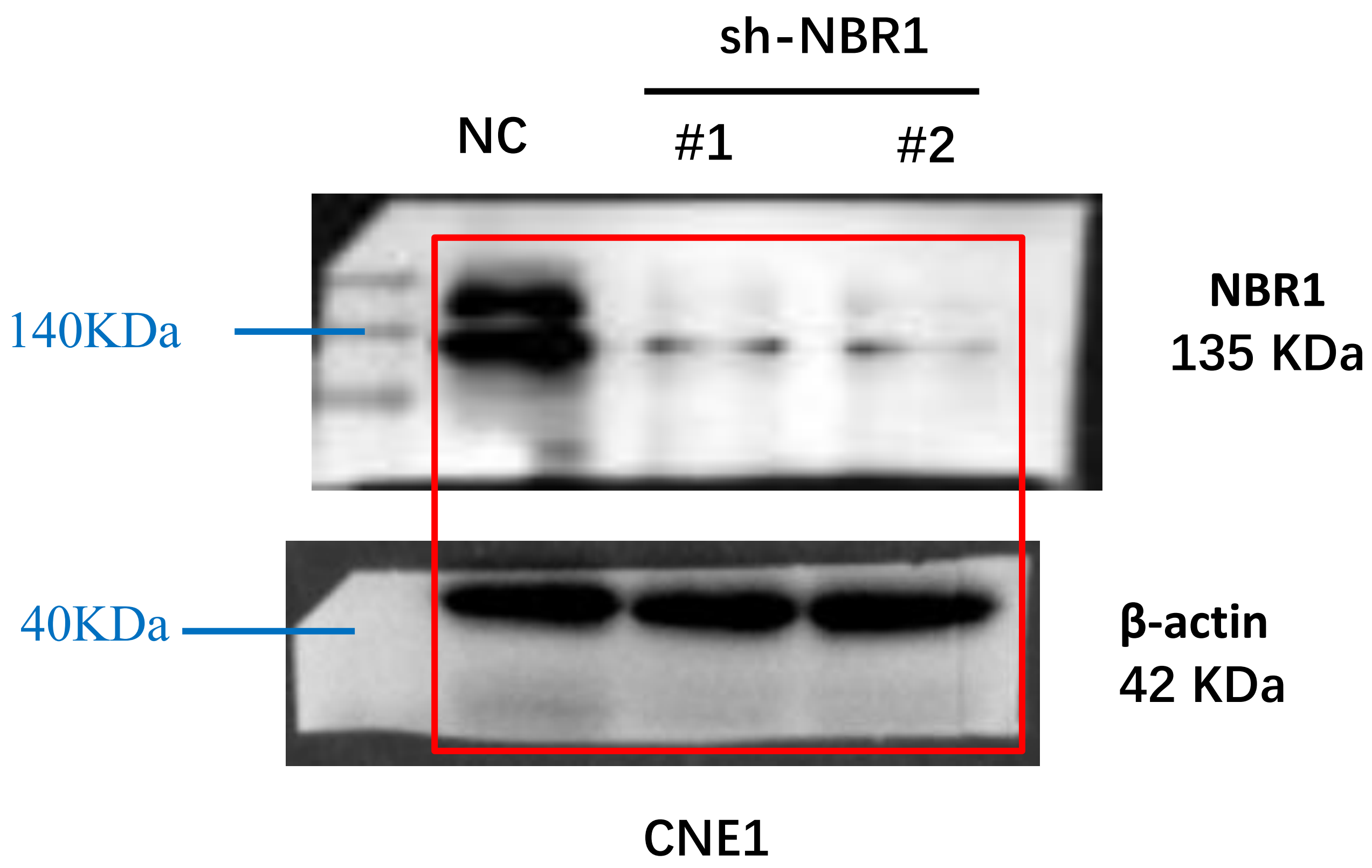

Supplementary Figure S4C

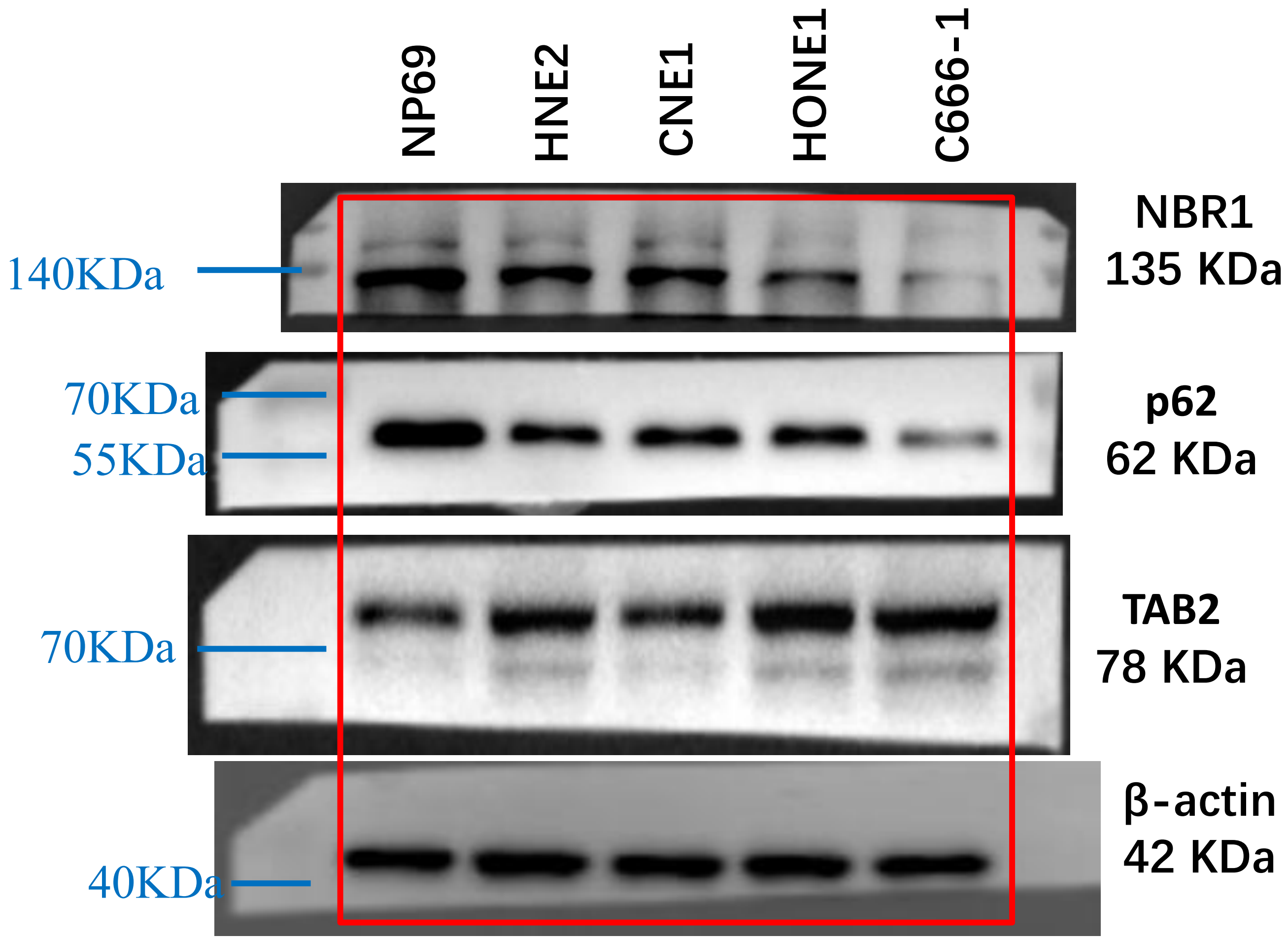

Supplementary Figure S4D

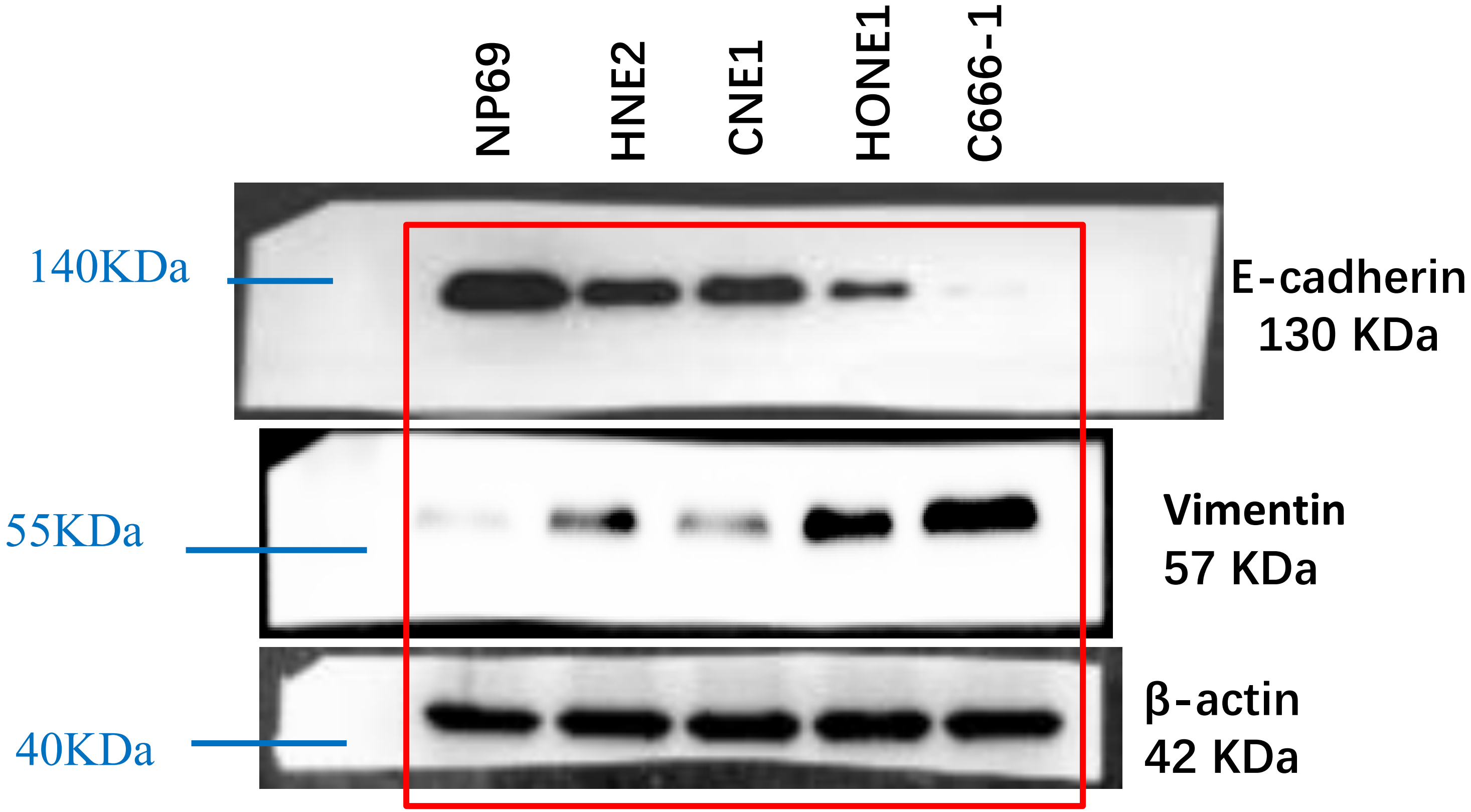

Supplementary Figure S5B

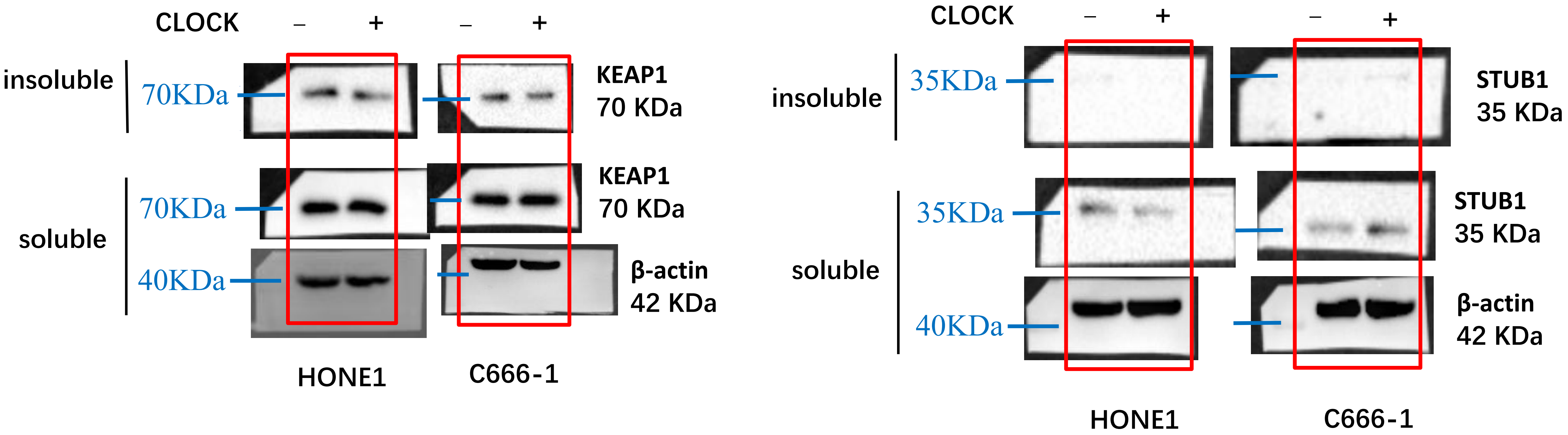

Supplementary Figure S6B

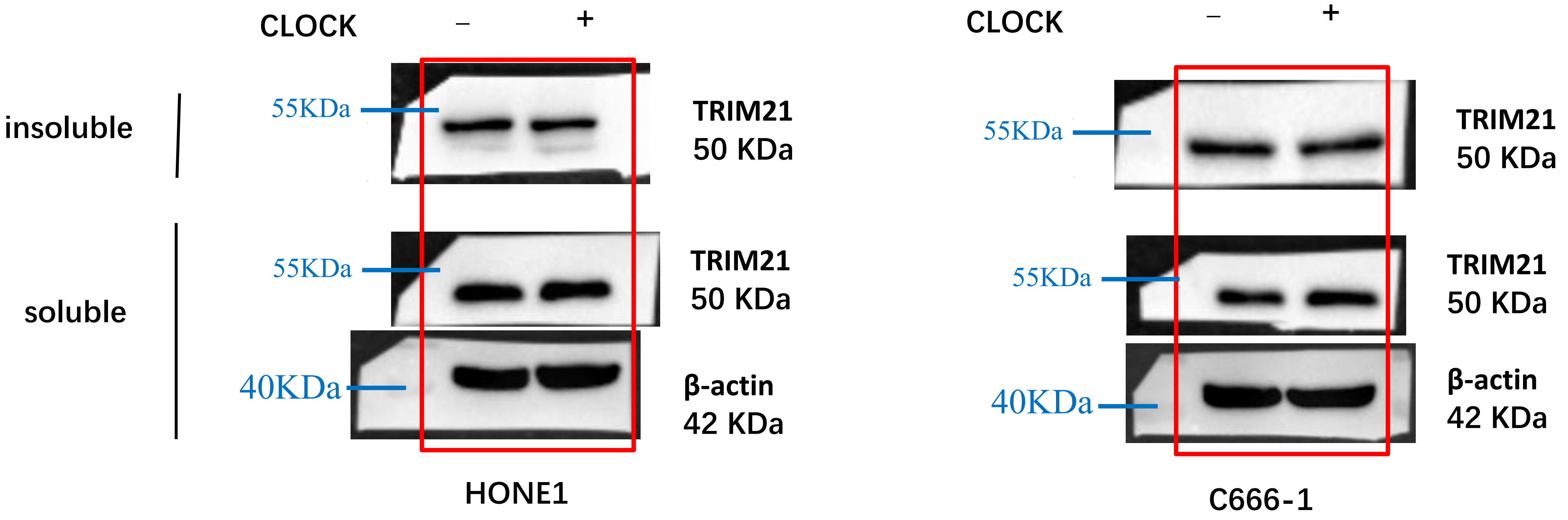

Supplementary Figure. S7

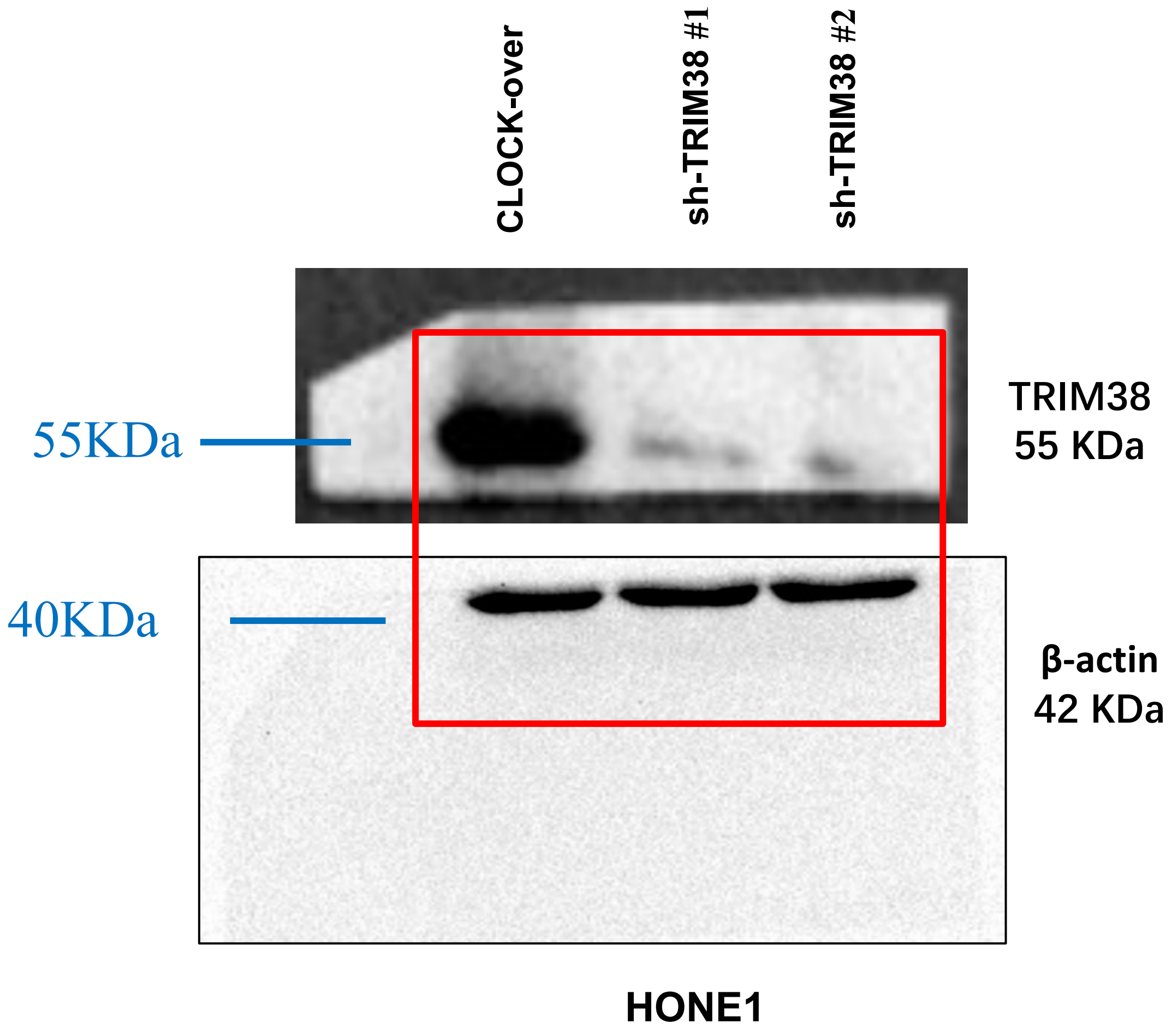

Supplement: Supplementary file 1 [file 41419_2026_8957_MOESM1_ESM.pdf]
