# Supplementary material for: Circadian gene CLOCK-mediated LLPS of NBR1 and p62/SQSTM1 inhibits proliferation and migration of nasopharyngeal carcinoma
Source: Cell Death Dis. 2026 Jun 10;17(1):734. doi: 10.1038/s41419-026-08957-x (PMC13478613; doi:10.1038/s41419-026-08957-x)
Supplement: Supplementary file 2 [file 41419_2026_8957_MOESM2_ESM.docx]

1. Supplementary materials and methods
   1.1. Bioinformatic analysis and protein-protein interaction network construction

Gene expression profiles and clinical information for head and neck squamous cell carcinoma (HNSCC) were retrieved from The Cancer Genome Atlas (TCGA-HNSC) database (<https://portal.gdc.cancer.gov/>), including 522 tumor tissues and 44 normal controls. After excluding cases with incomplete survival data, 449 tumor samples were retained for downstream survival analysis. Differential expression analysis between tumor and normal samples was performed using the R package DESeq2, with thresholds set at |log₂ fold change| ≥ 1 and an adjusted p-value (padj) < 0.05. Functional enrichment of differentially expressed genes (DEGs) was carried out via Gene Ontology (GO) analysis using the clusterProfiler package.

To assess the prognostic relevance of circadian rhythm–related genes, a curated set of 34 circadian genes was obtained from the KEGG pathway database (<https://www.genome.jp/kegg/>).

For the 449 tumor samples included in the survival analysis, patients were classified into high-expression and low-expression groups based on the median expression levels of each gene. Univariate Cox regression analysis was conducted using the R package survival to evaluate the association between gene expression levels and overall survival, with a statistical significance threshold set at p < 0.05. Genes that reached significance in the univariate analysis were further subjected to multivariate Cox regression analysis.

To analyze the expression patterns and prognostic value of the CLOCK gene in HNSCC, expression data were obtained from all 44 normal samples and 449 tumor samples, comparing expression levels between tumor and normal tissues. Based on the median expression level of CLOCK, the 449 tumor patients were classified into a high-expression group (n = 225) and a low-expression group (n = 224). Survival curves were plotted using the Kaplan–Meier method, and the log-rank test was applied to assess differences in overall survival between the two groups.

All data processing, statistical analyses, and result visualizations were performed in the R software environment (version 4.4.1).

Expression correlations between CLOCK and selected target genes were analyzed via the GEPIA web server (<http://gepia.cancer-pku.cn/>) using log₂-transformed transcripts per million (TPM) values from TCGA-HNSC (n = 500). Pearson correlation coefficients (R) and p-values were calculated. For GEO dataset GSE102349, log₂-transformed expression values for CLOCK, NBR1, ALDH9A1, and GAB2 were obtained from 113 NPC samples, and Spearman correlation analysis was conducted to assess their association.

To further explore the regulatory network of CLOCK, protein–protein interaction (PPI) networks for NBR1 were constructed using BioGRID, GeneMANIA, STRING, and the Human Protein Atlas, with overlapping candidate genes visualized using Venny.

Finally, bioinformatic prediction of putative CLOCK-binding sites within the NBR1 3′UTR was performed using the JASPAR database (<http://jaspar.genereg.net/>), providing candidate regulatory elements that may mediate CLOCK-dependent transcriptional control of NBR1.

1.2. Antibodies and reagents

The following primary antibodies were used in this study: anti-GFP-Tag rabbit mAb (AB0045) from Abways Biological Technology; anti-Flag-Tag rabbit mAb (DB14351), TAB2 Recombinant Rabbit mAb (db16524), and anti-E-Cadherin (DGR11268) rabbit mAb from Diagbio; anti-P62 mouse mAb (F2531) and anti-P62 rabbit mAb (F0106) from Selleck; anti-NBR1 mouse mAb (A32800) from Nature Biosciences; anti-TRIM38 rabbit antibody (O00635) and anti-TRIM21 rabbit antibody (P19474) from CUSABIO Technology LLC; anti-Myc-Tag rabbit mAb (R1208-1) and anti-β-actin mouse mAb (EM21002) from Huaan Biological Technology; anti-CLOCK mouse mAb (sc-271603) from Santa Cruz Biotechnology (California, USA); anti-Vimentin XP® rabbit mAb (D21H3, #5741) from Cell Signaling Technology; anti-KEAP1 rabbit polyclonal antibody (D223425) from Beijing Biodragon Immunotechnologies Co., Ltd; and rabbit anti-STUB1 (A11751) from ABclonal (Wuhan, China). Secondary antibodies included HRP-conjugated anti-mouse IgG (SC-2005), HRP-conjugated anti-rabbit IgG (SC-2004) from Santa Cruz Biotechnology; and Alexa Fluor 594-conjugated anti-rabbit IgG (HA1122) from Huaan Biological Technology.

Additional reagents included Lipomax transfection reagent (P/N32012) from SUDGEN Biological Technology; Phanta Max Super-Fidelity DNA Polymerase (P505-d1) from Vazyme Biotech Co., Ltd.; Cocktail（HY-K0010) from MCE; pre-stained protein marker ([C610015](https://store.sangon.com/productDetail?productInfo.code=C610015)) from Sangon Biotech Co., Ltd.; protein A/G magnetic beads (L-1004A) from Biolinkedin Biotechnology Co., Ltd.; co-immunoprecipitation lysis buffer (PC105) from Epizyme Biotech Co., Ltd.; Cell Counting Kit-8 from DOJINDO (Cat. CK04, Kyushu, Japan); RevertAid First Strand cDNA Synthesis Kit (K1622) from Invitrogen, Carlsbad, CA, USA; TRIzol® reagent (15596026) from Ambion® by Life Technologies, USA; DAPI (abs47047616) from Absin Biological Technology; MG-132 (M7449), and Chloroquine diphosphate (C6628) from Sigma-Aldrich, St. Louis, MO, USA；1,6-Hexanediol (99.5%) from Macklin, Shanghai, China; dual luciferase reporter gene assay kit (RG027) from Beyotime; and DpnI enzyme (15052ES50) from Yeasen Biotech Co., Ltd.

1.3. Fluorescence recovery after photobleaching (FRAP) assay

HONE1 cells were seeded onto 35 mm glass-bottom culture dishes and cultured for approximately 12 h until reaching 50–60% confluence. Cells were transfected using Lipomax transfection reagent. For the experimental group, cells were co-transfected with plvx-CLOCK, Flag-NBR1, and GFP-P62 plasmids, whereas the control group was co-transfected with empty plvx vector, Flag-NBR1, and GFP-P62. Forty-eight hours after transfection, live-cell FRAP analysis was performed using a Leica TCS SP8 confocal microscope. Photobleaching was performed using a 488-nm laser at 90% maximum power for five cycles, followed by monitoring fluorescence recovery for 300 s. GFP fluorescence was detected using a 63× oil-immersion objective. FRAP recovery curves were generated for each bleached condensate, and the average recovery time and standard deviation (SD) were calculated.

1.4. Detergent-soluble and detergent-insoluble fractionation

Cells were seeded in 6 cm culture dishes and grown to approximately 80% confluence. For protein fractionation, cells were washed twice with ice-cold PBS and lysed on ice for 20 min in Triton X-100 lysis buffer (50 mM Tris-HCl, pH 7.5; 150 mM NaCl; 1% Triton X-100) supplemented with a protease inhibitor cocktail. Lysates were centrifuged at 13,000 rpm for 15 min at 4°C to separate the detergent-soluble fraction (supernatant) from the insoluble fraction (pellet). Protein concentrations of the soluble fractions were quantified using a BCA protein assay kit. The pellet was washed once with Triton buffer, resuspended in 5× SDS-PAGE loading buffer, and boiled for 10 min at 95°C. Both soluble and insoluble fractions were subjected to SDS-PAGE, transferred onto PVDF membranes, and analyzed by Western blotting using antibodies against p62, NBR1, TRIM38, and TAB2, with β-actin serving as the loading control.

1.5. Ubiquitination assay

HONE1 or HONE1-CLOCK cells were seeded in 10-cm culture dishes and cultured for approximately 12 h until reaching 70%–90% confluence. Cells were transfected with the indicated plasmids using Lipomax transfection reagent. After 6 h, the medium was replaced with fresh medium containing 3% fetal bovine serum. Forty-eight hours post-transfection, cells were harvested and lysed in immunoprecipitation lysis buffer supplemented with 10% protease inhibitor cocktail for 2 h at 4°C. MG132 (20 μM) was added to the culture medium 8 h prior to cell collection.

Cell lysates were centrifuged at 12,000 rpm for 5 min at 4°C, and the supernatants were transferred to new tubes. Protein concentrations were determined using the BCA assay. For pre-clearing, 2 mg of total protein was incubated with 20 μL of protein A/G magnetic beads at 4°C for 1 h. The beads were then removed using a magnetic stand, and the supernatants were transferred to fresh tubes and incubated with 2 μg of anti-TAB2 antibody at 4°C for 12–15 h to form immune complexes. Subsequently, 30 μL of protein A/G magnetic beads were added and incubated at 4°C for an additional 2–3 h. The protein–antibody–bead complexes were washed five times with ice-cold PBS, resuspended in 30 μL of 5× loading buffer, and boiled for 10 min. The samples were then subjected to Western blot analysis.

1.6. Proteasome and lysosome inhibition assay

To determine the degradation pathway of target proteins, HONE1 and C666-1 cells at 80–90% confluence were divided into experimental and control groups. Experimental cells were treated with complete culture medium containing either 20 μM MG132, a reversible proteasome inhibitor that blocks the proteolytic activity of the 26S proteasome complex, or 30 μM chloroquine (CQ), an inhibitor of lysosomal acidification and autophagy. Control cells received medium supplemented with an equivalent volume of DMSO. All cells were incubated at 37°C with 5% CO₂ for 8 h. Following treatment, the culture medium was removed, and cells were washed three times with pre-cooled PBS. Cells were then lysed on ice for 30 min in RIPA buffer containing 1% protease inhibitor cocktail. Lysates were collected using a cell scraper and centrifuged at 13,000 rpm for 15 min at 4°C. The supernatant was collected for protein quantification using the BCA assay, followed by Western blot analysis to assess protein expression levels.

1.7. Immunohistochemical analysis

Tumor tissue sections from routine diagnostic biopsies at Xiang-Ya Hospital of Central South University were fixed in 4% formaldehyde, embedded in paraffin, and sectioned. This study was approved by the Ethics Committee of Xiang-Ya Hospital, Central South University. Written informed consent was obtained from all patients prior to biopsy and sample collection. Archived human tissue specimens and corresponding clinical information were used exclusively for research purposes in accordance with institutional regulations and the Declaration of Helsinki. A total of 46 samples were analyzed, including 23 tumor tissues and 23 adjacent normal tissues. Sections were baked to ensure adherence, deparaffinized using a dewaxing agent (Solarbio, China), and rehydrated through graded ethanol. Antigen retrieval was performed in citrate buffer using microwave heating. Sections were permeabilized with 0.2% Triton X-100, and endogenous peroxidase activity was blocked with 3% hydrogen peroxide. Non-specific binding was prevented by incubation with normal goat serum. Primary antibodies against CLOCK, NBR1, p62, and Ki-67 were applied overnight at 4°C, followed by incubation with the appropriate secondary antibody. Immunoreactivity was visualized using DAB (Zsgbbio, China), counterstained with hematoxylin, dehydrated, and mounted. Immunohistochemical staining was semi-quantitatively evaluated using a histoscore system (range 0–12), which was used for comparative analysis between normal and tumor tissues. For descriptive purposes in clinicopathological tables, histoscores were additionally converted into percentages of the maximum possible score (12). Clinical information of NPC patients, including age, gender, TNM staging, pathological diagnosis, and EBV status determined by EBER in situ hybridization, is summarized in Supplementary Table S3. Clinical characteristics of nasopharyngitis cases used as normal controls are provided in Supplementary Table S4.

2. Supplementary figure legends

Supplementary Figure S1. Scatter plots showing the correlation between log₂(CLOCK) and log₂(NBR1), log₂(ALDH9A1), and log₂(GAB2) expression in 113 NPC samples from GEO (GSE102349). Spearman correlation analysis revealed a statistically significant positive association between CLOCK and NBR1 (ρ = 0.186, P = 0.0485) (A). In contrast, ALDH9A1 (ρ = 0.16, P = 0.079) (B) and GAB2 (ρ = 0.03, P = 0.75) (C) showed no statistically significant correlations.

Supplementary Figure S2. NBR1 knockdown promotes NPC cell proliferation and migration. (A) qRT-PCR and Western blot analyses were performed to confirm efficient knockdown of NBR1 in stably transfected CNE1 cells. (B) The CCK-8 assay was performed to assess cell viability in CNE1-NC and CNE1-sh-NBR1 cells at days 0, 1, 2, and 3. (C) Colony formation assays were conducted to evaluate clonogenic capacity of CNE1-NC and CNE1-sh-NBR1 cells, with colonies fixed, stained, and counted after 15 days of culture. (D, E) Wound healing and transwell migration assays were performed to assess the migratory ability of CNE1 cells following NBR1 knockdown. Data are presented as mean ± SD from three independent experiments. *, and ** indicate p < 0.05, and p < 0.01, respectively.

Supplementary Figure S3. Bioinformatics-based identification of p62 as a potential NBR1-associated target and its transcriptional validation. (A) PPI networks of NBR1-associated proteins were constructed using the BioGRID, GeneMANIA, STRING, and Human Protein Atlas databases. (B) A Venn diagram illustrating the overlap of NBR1-interacting genes identified across the four databases. (C) The mRNA expression level of p62 was analyzed by RT–qPCR in HONE1 and C666-1 cells with or without CLOCK overexpression, as well as in CNE1 and HNE2 cells with or without CLOCK knockdown. Data are presented as mean ± SD from three independent experiments. *ns* indicates no significant difference. PPI, protein–protein interaction.

Supplementary Figure S4. Expression profiles of NBR1 and p62 in NPC cell lines and their association with migratory potential. (A, B) qRT–PCR was performed to quantify NBR1 and p62 mRNA expression levels in NPC cell lines (HNE2, CNE1, HONE1, and C666-1) and the normal nasopharyngeal epithelial cell line NP69. (C, D) Western blot analyses were performed to examine protein expression of NBR1, p62, and TAB2 (C), as well as the EMT markers E-cadherin and Vimentin (D), in NPC cell lines and NP69 cells. (E, F) Wound healing and transwell migration assays were conducted to assess the migratory capacity of CNE1 and HONE1 cells. Wound closure was monitored at 0 and 48 h (E). For the transwell migration assay, cells were fixed and stained with crystal violet at 48 h (F). Data are presented as mean ± SD from three independent experiments. *, **, and *** indicate p < 0.05, p < 0.01, and p < 0.001, respectively; ns indicates no significant difference.

Supplementary Figure S5. Bioinformatic identification and experimental validation of shared NBR1/p62-interacting proteins. (A) A Venn diagram illustrating the overlap between NBR1- and p62-interacting proteins retrieved from the STRING database, with KEAP1 and STUB1 highlighted among the 136 shared candidates. (B) Western blot analyses of detergent-soluble and detergent-insoluble fractions were performed to assess KEAP1 (left) and STUB1 (right) protein levels in HONE1 and C666-1 cells following CLOCK overexpression.

Supplementary Figure S6. Bioinformatics analysis of p62-interacting proteins and experimental validation of TRIM21 expression. (A) A PPI network of p62-interacting proteins was constructed based on BioGRID data. (B) Western blot analysis of detergent-soluble and detergent-insoluble fractions was performed to evaluate TRIM21 protein expression in HONE1 and C666-1 cells following CLOCK overexpression. PPI, Protein-protein interaction.

Supplementary Figure S7. Validation of TRIM38 knockdown efficiency in NPC cells. Western blot analysis was performed to validate the efficiency of TRIM38 knockdown mediated by two independent shRNAs (sh-TRIM38#1 and sh-TRIM38#2) in NPC cells.

Supplementary Table S1. q RT-PCR primers

Supplementary Table S2. Primer sequences for cloning and mutagenesis

Supplementary Table S3. Clinicopathological characteristics of NPC patients

Supplementary Table S4. Clinical characteristics of nasopharyngitis cases
